# Massive NGS data analysis reveals hundreds of potential novel gene fusions in human cell lines

**DOI:** 10.1093/gigascience/giy062

**Published:** 2018-06-01

**Authors:** Silvia Gioiosa, Marco Bolis, Tiziano Flati, Annalisa Massini, Enrico Garattini, Giovanni Chillemi, Maddalena Fratelli, Tiziana Castrignanò

**Affiliations:** 1 SCAI-Super Computing Applications and Innovation Department, CINECA, Rome, Italy; 2 National Council of Research, CNR, Institute of Biomembranes, Bioenergetics and Molecular Biotechnologies, Bari, Italy; 3 Laboratory of Molecular Biology, IRCCS-Istituto di Ricerche Farmacologiche “Mario Negri,” Milano, Italy; 4 Computer Science Department, Sapienza University of Rome, Italy

**Keywords:** database, human gene fusions, malignant cell lines, NGS, gene fusion detection algorithms, chromosomal rearrangements, bioinformatics

## Abstract

**Background:**

Gene fusions derive from chromosomal rearrangements. The resulting chimeric transcripts are often endowed with oncogenic potential. Furthermore, they serve as diagnostic tools for the clinical classification of cancer subgroups with different prognosis and, in some cases, they can provide specific drug targets. To date, many efforts have been carried out to study gene fusion events occurring in tumor samples. In recent years, the availability of a comprehensive next-generation sequencing dataset for all existing human tumor cell lines has provided the opportunity to further investigate these data in order to identify novel and still uncharacterized gene fusion events.

**Results:**

In our work, we have extensively reanalyzed 935 paired-end RNA-sequencing experiments downloaded from the Cancer Cell Line Encyclopedia repository, aiming at addressing novel putative cell-line specific gene fusion events in human malignancies. The bioinformatics analysis has been performed by the execution of four gene fusion detection algorithms. The results have been further prioritized by running a Bayesian classifier that makes an *in silico* validation. The collection of fusion events supported by all of the predictive software results in a robust set of ∼1,700 *in silico* predicted novel candidates suitable for downstream analyses. Given the huge amount of data and information produced, computational results have been systematized in a database named LiGeA. The database can be browsed through a dynamic and interactive web portal, further integrated with validated data from other well-known repositories. Taking advantage of the intuitive query forms, the users can easily access, navigate, filter, and select the putative gene fusions for further validations and studies. They can also find suitable experimental models for a given fusion of interest.

**Conclusions:**

We believe that the LiGeA resource can represent not only the first compendium of both known and putative novel gene fusion events in the catalog of all of the human malignant cell lines but it can also become a handy starting point for wet-lab biologists who wish to investigate novel cancer biomarkers and specific drug targets.

## Background

Oncogenic gene fusion events result from chromosomal rearrangements that lead to the juxtaposition of two previously separated genes. The accidental joining of DNA of two genes can generate hybrid proteins. It can also result in the misregulation of the transcription of one gene by the cis-regulatory elements (promoters or enhancers) of another, sometimes resulting in the production of oncoproteins that bring the cell to a neoplastic transformation [1]. Not only can gene fusions have a strong oncogenic potential [2] but they can also serve as diagnostic tools for the clinical classification of cancer subgroups with different prognosis and, in some cases, they may provide specific drug targets [3]. For instance, the presence of the PLM-RARA fusion product is a specific hallmark of acute promyelocytic leukemia [4] and represents the first example of gene-fusion targeted therapy [5] that has changed the natural history of this disease. Hence, there are several reasons why studying gene fusions in cancer is very important. In recent years, next-generation sequencing (NGS) technologies have played an essential role in the understanding of the altered genetic pathways involved in human cancers. Today, most studies aiming at fusion discovery use NGS techniques followed by massive bioinformatics analyses. The greatest challenge of these sophisticated algorithms of prediction is the ability to discriminate between artifacts and the actual chromosomal rearrangements that occur [6]. Moreover, each gene fusion predicting software differs in terms of sensitivity and specificity. In the last decade, much effort has been made to catalog gene fusion events, thus resulting in a wide production of databases. At present, a dozen published databases regarding oncogenic fusion genes exists (see Table1 for a summary). Some of them (e.g., FusionCancer, ChiTaRS-3.1) collect *in silico* predictions of chimeric genes, obtained by analyzing publicly available datasets derived from heterogeneous sources in terms of experimental material (a mix of single-end and paired-end RNA-sequencing [RNA-seq] data, expressed sequence tags) or in terms of the data source (patients and cell lines). Others collect gene fusion events with experimental evidence manually curated from literature collections (e.g., Tumor Cancer Genome Atlas [TCGA], Mitelman, TICdb, COSMIC, ONGene). Here, we focused on the whole catalog of human malignant cell lines, thus obtaining a homogeneous input NGS dataset covering several human malignancies. We performed a massive bioinformatics analysis on 935 paired-end RNA-seq samples derived from 22 different tumor tissues and used a combination of the best-performing gene fusion-detecting algorithms. For ease of understanding, we define the predicted gene fusion event (pGFE) as the entity constituted by the gene fusion couple in a specific cell line and designate the consensus call-set (CCS) as the number of pGFEs supported by all the used algorithms. Starting from this assumption, we obtained 377 ,540 pGFEs, 2,521 of which belong to the CCS. Moreover, since not all of the pGFEs can give rise to oncogenic transformations, the use of a prioritization software is recommended in order to distinguish between real driver mutations and passenger mutations. Therefore, a robust Bayesian classifier has been used to perform an *in silico* validation of the results. Since one of the main purposes of this big data analysis is to encourage the re-use of our results in order to experimentally validate the *in silico* predictions, we set up a web portal to collect and systematize these data, LiGeA (cancer cell LInes Gene fusion portAl). It is possible to browse, search, and freely download all the results obtained and described within this article at the LiGeA repository web page available at [7]. To our knowledge, this represents the first compendium of both known and predicted novel gene fusion events in cell lines from 22 different human tumor types.

**Table 1:** State-of-the-art of databases reporting gene fusions

Database name	Short description
Tumor Fusion Gene Data Portal [8]	A collection of fusion genes in the TCGA samples
TICdb [9]	A collection of 1,374 fusion sequences extracted either from public databases or from published papers (last update: 2013)
chimerDB3.0 [10]	A catalog of fusion genes encompassing analysis of TCGA data and manual curations from the literature
COSMIC Cell Lines [11]	Gene fusions are manually curated from peer-reviewed publications. Currently, COSMIC includes information on fusions involved in solid tumors but not yet leukemias and lymphomas
Mitelman [1]	Reports hundreds of gene fusions associated with clinical reports but does not contain sequence data
ChiTaRs-3.1 [12]	A collection of 34,922 chimeric transcripts identified by expressed sequence tags and mRNAs from the GenBank, ChimerDB, dbCRID, TICdb, and Mitelman collections of cancer fusions for several organisms
FusionCancer [13]	Includes 591 samples, both single-end and paired-end RNA-seq, published on the sequence read archive database [14] between 2008 and 2014 covering 15 types of human cancers
ONGene [15]	Literature-derived database of oncogenes

## Data Description

### Methods

We analyzed 935 paired-end RNA-seq experiments available at the Cancer Cell Line Encyclopedia repository [16], for a total of 32 TB of input raw data. The analysis was carried out by using four somatic fusion gene detection algorithms: FusionCatcher [17], EricScript [18], Tophat-Fusion [19], and JAFFA [20]. The choice of the algorithms was driven by the assessment from Kumar et al. [21], who compared 12 methods for the fusion transcripts detection from RNA-seq data and identified these software programs as the ones with the highest positive prediction values. Furthermore, the chosen software programs differ in a variety of aspects and contain several layers of information in their output files, thus giving us the opportunity to collect and interconnect a wide set of complementary data for each pGFE. Following is a short description of each fusion detection tool, accompanied by the used versions and parameters.:

- **FusionCatcher (FC)**: FC is a Python-based algorithm. It executes a first mapping run with Bowtie v.1.2.0 [22] and then performs the gene fusion detection based on three aligners: Bowtie2 v.2.2.9 [23], BLAT v.36 [24], and STAR v.2.5.2b [25]. FC takes advantage of National Center for Biotechnology Information viral genomes (v. 2016-01-06) in order to detect exogenous virus material integration into the host genome. Moreover, the FC algorithm compares its own output with a set of published databases, thus providing a detailed list of truly positive and false-positive pGFEs candidates. In our analysis, we downloaded FC v. 0.99.5a and Ensembl genome annotation v.83 and used hg38/GRCh38 as the genome assembly version. The software was executed with default parameters, requiring 111, 620 central processing unit (CPU) core hours, 125 GB of random access memory (RAM), and 20 CPU to complete the execution on our input dataset. Overall, FC detected  25,251 pGFEs involving 8,659 genes.
- **Tophat-Fusion (TF)**: TF uses the TF-post function in order to create a filtered list of gene fusion candidates, starting from the output files obtained by running Tophat with the “–fusion-search” option [26]. The following commands were run subsequently:
  tophat -o $Sample.output/ -p 20 –fusion-search –keep-fasta-order –bowtie1 –no-coverage-search -r 160 –mate-std-dev 34 –max-intron-length 100000 –fusion-min-dist 100000 –fusion-anchor-length 13 $BOWTIE_INDEX/hg38 $Sample_1.fastq $Sample_2.fastq
  cd $Sample.output/
  tophat-fusion-post -p 20 –skip-blast $BOWTIE_INDEX/hg38

Tophat-2.0.12 and samtools 0.1.19 versions were used for this study. This algorithm took about  200,000 CPU core hours, 20 CPU, and 125 GB of RAM in order to complete its runs on the whole input dataset. TF produces several output files, but only the file named “results.txt,” representing the filtered list of predicted gene fusions, was used for subsequent analysis. The results encompassing “Chromosome M” have been manually discarded from the final results *in primis* because TF and JF were the only two of the four algorithms reporting them and because they represented *bona fide* false-positive outcomes. Overall, TF highlighted  28,146 pGFEs involving 9,492 genes.

- **JAFFA (JA)**: JAFFA (v. 0.9) is a multistep pipeline that takes raw RNA-seq reads and outputs a set of candidate fusion genes along with their cDNA breakpoint sequences. It relies on trimmomatic [27], samtools [28], BLAT [24], bowtie2, bpipe [29], and R software programs [30], as well as on gencode (v. 22), for the annotation and on the Mitelman database for flagging already known gene fusions. For the purpose of this analysis, we used the “Direct” mode pipeline, which is indicated for reads of 100 bp or longer. A total of   1,300,000 CPU core hours, 125 GB of RAM, and 20 CPU were required to successfully complete the analysis. The results encompassing “Chromosome M” have been manually discarded from the final results. Furthermore, only pGFEs supported by at least three spanning reads or flagged as “known” have been retained. Overall, after the filtering process, JA detected  53,400 pGFEs involving  12,256 genes.
- **EricScript (ES)**: ES is developed in R, perl, and bash scripts. It uses the BWA aligner [31] to perform the mapping on the transcriptome reference and samtools v. 0.1.19 to handle SAM/BAM files. Recalibration of the exon-junction reference is performed using BLAT. For the purposes of this project, we used BLAT v.36, R v.3.3.1, bedtools v. 2.24, and ES version 0.5.5. We obtained and built the Ensembl Database v. 84 [32] by using BWA software with the command:
  bwa index -a bwtsw allseq.fa. A total of  130,900 CPU core hours, 125 GB of RAM, and 20 CPU were required to successfully complete the analysis. We further filtered out ES final results by removing all the predictions for which the software was not able to predict an exact breakpoint position because such pGFEs could not even be experimentally validated. Second, as also applied to FC, TF, and JF results, we retained the pGFEs exhibiting at least three spanning reads over the gene fusion junction. Furthermore, we filtered out all pGFEs with an EricScore value less than 0.85. EricScore is a ranking parameter ranging from 0.5 to 1; greater values correspond to better predictions. Interestingly, by applying these filters, we filtered out almost two-thirds of the initial predictions from EricScript but, at the same time, the CCS did not reduce substantially, thus indicating that the choice of a consensus of predictions is a good strategy to remove false positives and obtain a reliable set of gene fusion candidates to be experimentally validated. Overall, after the filtering process, ES detected  293,220 pGFEs involving  14,740 genes.

### Data statistics and validation

**Figure 1:**
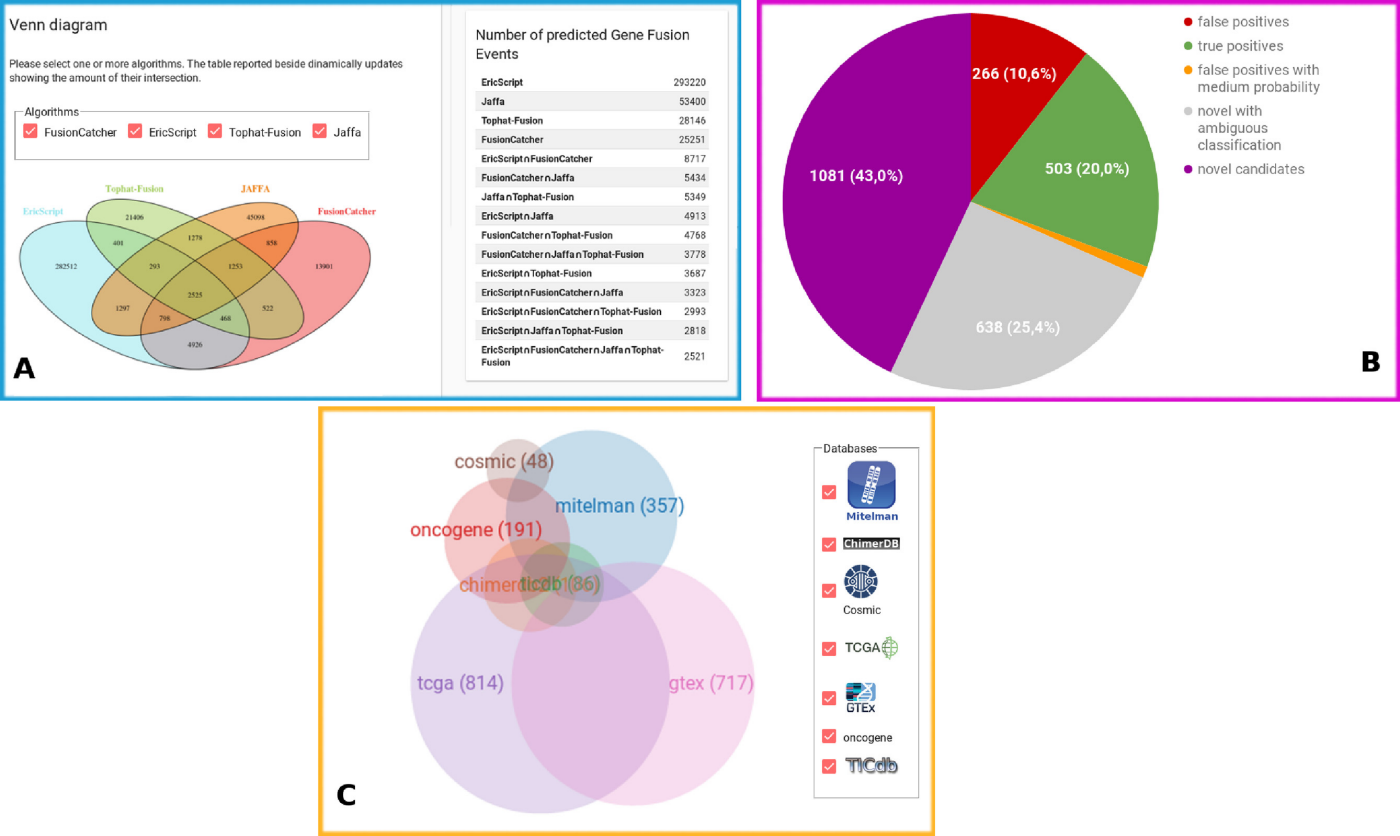
**(A)** Venn diagram showing the intersection of the pGFEs identified by the four algorithms. **(B)** Distribution of pGFEs in the CCS; 43% (purple) of the CCS has not been previously described in any other database or scientific publication; 10% (red) and 20% (green) of the CCS have been reported in databases from healthy/tumoral samples, thus representing the false-positive/true-positive subset of our analysis; 1% of the CCS (orange) reports tags that classify the pGFE as a false-positive couple with medium probability; 25% (gray) of the results represent novel pGFEs tagged with values that classify them as both false positives and true positives. **(C)** Venn diagram showing the intersection between the LiGeA CCS and other databases.

Overall, our extensive analysis results in a CCS of 2,521 pGFEs (Fig.1A) and, respectively, 2,828/9,258 pGFEs supported by exactly three/two methods. As the first validation of our analysis, 661 of the 719 (92%) genes known to be functionally implicated in cancer and collected under the COSMIC gene census are present in our final dataset. As a further validation of our results, about 1/5 of our CCS has already been published or is present in the following databases: chimerdb3, ONGene, COSMIC, TCGA, ticdb, and Mitelman (Fig.1C). Finally, only a small subset of the pGFEs (∼10% of data) present in the CCS has been recognized as false-positive predictions, thus supporting the idea that a combination of algorithms can be of great utility in order to increase the sensitivity and the specificity of the tests. It is worth mentioning that not only did our analysis confirm a large number of known gene fusion events, it also highlighted 1,719 novel putative pGFEs in the CCS that could undergo further downstream analysis (Fig.1B). Therefore, a further step of analysis was run with Oncofuse v.1.1.1 [33] in order to distinguish driver mutations (genomic abnormalities responsible for cancer) from passenger mutations (inert somatic mutations not implicated in carcinogenesis). Oncofuse is considered an *in silico* validation post-processing step that prioritizes the results obtained from each of the four algorithms. It assigns a functional prediction score to each putative fusion sequence breakpoint identified by the four software programs, thus hinting that pGFEs are worthy of being experimentally validated and studied. Oncofuse supports multiple input formats such as the output from TF and FC. In order to run it on the outputs from ES and JF, a short pre-processing step was executed on these data. As suggested in the Oncofuse manual, the accepted default input format is a tab-delimited file with lines containing 5΄ and 3΄ breakpoint positions. Therefore, these columns were extracted from ES and JF output files and redirected into Oncofuse-accepted input format. Oncofuse was run with default parameters using hg38 as the reference genome.

## Database Description

LiGeA is a database server based on graph-db technology (Neo4j). The portal stores all of the results obtained from each fusion gene predicting algorithm and the prioritization analysis outcome. This database contains not only a mere collection of *in silico* predictions, it has been integrated with other useful external resources in order to offer a carefully curated web compendium. Here is a short list of the added features:

- Whenever the gene fusion couple has already been experimentally validated and published, an extra column with the COSMIC icon is added to the results. By clicking on it, the user will be redirected to an external link containing a manually curated catalog of 212 literature-derived somatic mutations in cancer [34].
- Cancer Gene Census is a manually curated catalog of 719 genes for which mutations have been causally implicated in oncogenesis [35]. Whenever one of the two genes involved in the pGFE has already been described to be implicated in cancer, the gene is tagged with an icon. By clicking on it, an external link to the Cancer Gene Census is provided, showing a table of genes included within this category [36].
- A legend based on a colorful signature has been added to tag the FC predictions as "validated truly positive couples" (green circle), "validated false-positive couples" (red circle), "false-positive couples with medium probability" (orange circle), and "ambiguous signature" because it is tagged with both positive and negative values (gray circle).
- A functional prediction score obtained by extensively running the Oncofuse software is reported as an additional tag to the outputs from each algorithm.

The LiGeA portal is divided into several sections that allow a user-friendly navigation:

**Home:** On the homepage, the user is provided with a quick overview of the database. A global summary table reports a numeric recapitulation (e.g., the number of genes/transcripts/exons collected into the portal, the number of predicted proteins). Moreover, a histogram shows an abstract of the top 50 involved cell lines. By moving the cursor on the bars, a pop-up opens showing the cell line name and the corresponding number of unique fusion events predicted by all of the algorithms. Information about the algorithm predictions hosted into the portal are supplied with an interactive Venn diagram linked to a dynamic table. Upon user selection of the algorithm/s of interest, both the diagram and the table refresh, thus showing the resulting number of intersections.

**Figure 2:**
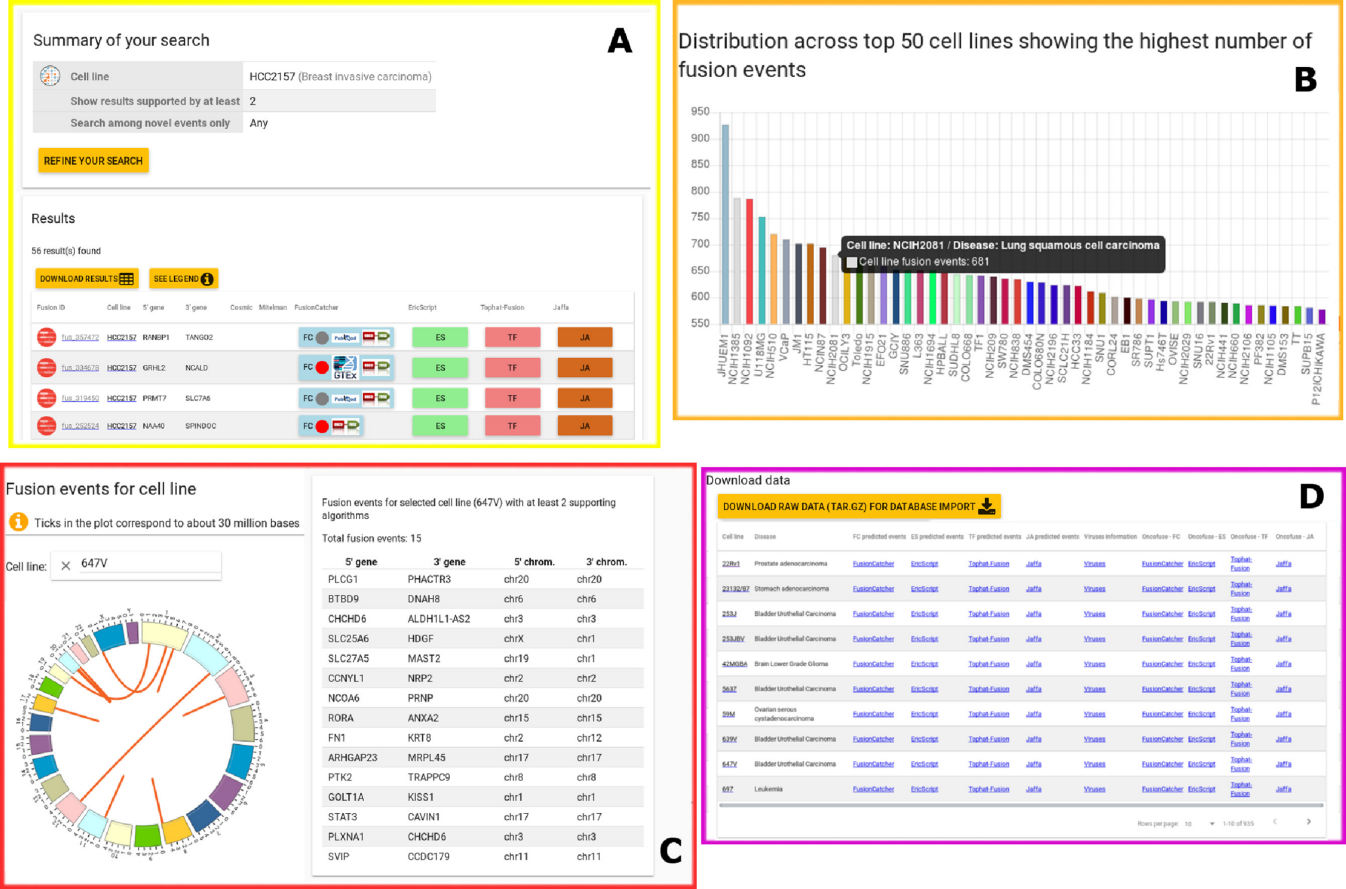
An overview of the LiGeA portal. **(A)** A "Search by Cell Line" example and the corresponding output. **(B)** An overview of the input dataset. **(C)** A circos diagram showing the graphical outcome of a "Query by Cell Line" and the corresponding related table. **(D)** An extract from the Download web page.

**Search:** This utility allows several searching options to browse and mine genomic fusion events stored in the LiGeA portal (see Table2 for an overview). All the resulting outputs are sorted by the number of algorithms supporting the fusion events, thus showing the most robust set of results on the top of the table. As an additional feature, when specifying the features of interest, it is also possible to choose the minimum number of predicting algorithms. Search results are presented in the form of a paginated table containing those fusion events that satisfy the query parameters; data can also be downloaded in tabular format. Furthermore, by clicking on a given fusion ID, it is possible to access the event-specific page on which relevant information is presented in greater detail (e.g., involved cell line, disease, and genes as well as links to external databases and resources). Two of the nine query forms ("search by fusion information" and "search by virus") are specific annotations-derived FC algorithms. Following is a short description of the provided searching utilities:

"Search by Disease": In this section, all the cell lines derived from the same disease have been grouped together. In this way, it is possible to navigate the gene fusions that putatively cause specific malignancies. The number of cell lines constituting the queried subset is shown next to the pathology name.
"Search by Cell Line": This module allows you to navigate the database by indicating a specific cell line name. It is possible to tune the results by showing only the novel predictions not yet described in any other database or publication (Fig. 2A).
"Search by Chromosome": This query can be performed by inserting one or two chromosomes involved in the fusion event. The cell line name can be indicated or not.
"Search by Gene": The user can select up to two gene names (Gene Symbol or ENSEMBL ID) and the "cell line" form can be either selected or not. The genes reported in the query form are black if they are involved in pGFE and gray if they are not.
"Search by Transcript": Since the same gene can give rise to different transcripts, it is be reasonable to query which of the transcripts produced by a specific gene are affected by a fusion event. This kind of query can be satisfied by inserting the Ensembl Transcript (ENST) IDs in the specific form.
"Search by Exon": Some of the queries allow you to go into more molecular detail. This search can be done by inserting one or two exon IDs involved in the fusion event. The cell line name can be indicated or not. In this way, it is possible to highlight the specific exons that turn out to be fused in the final result.
"Search by Fusion Information": The pGFEs may have different predicted effects. Indeed, depending on the location of the chromosomal break points, the resulting protein may be in-frame, out-of frame, truncated, and so on. Since the selectable values present in the fusion information form are specific to the FC algorithm, the result of this query returns a table without ES, JA, and TF data. We suggest reviewing the FC manual in order to obtain a full description of all tags.
"Search by Algorithm": This type of query is suitable for users who wish to navigate the outputs from specific software programs, choosing them individually or in combination. Indeed, it is known that some types of fusions, such as those involving immunoglobulins, can be detected by specific software [37].
"Search by Viruses": Additional useful information retrievable from the database relates to virus sequence integration into the host genome. This search utility is virus centered since it is possible to indicate or not the host cell line name. It is possible to select the virus name of interest (whether using GI ID or NC ID). Furthermore, a clickable link redirecting you to the virus genome is also shown on the right side of the table.

**Statistics:** This section allows you to visually inspect the results. The four submenus are organized as follows:

– "Cell Line Statistics": By choosing the cell line of interest, the resulting circular diagram shows all the chromosome couples involved in GFE predicted by at least two algorithms. The table on the right summarizes the resulting couples of the genes and chromosomes (Fig. 2C).
– "Chromosome Statistics": This page reports a dynamic pie chart showing the number of fusion events per human chromosome. By clicking on each slice of the pie, the related table automatically updates, showing a chromosome summary statistics. Furthermore, information about the number of inter- and intra-chromosomal rearrangements detected by each algorithm is also reported.
– "Disease Statistic"’: The Fusion Statistics pie chart was produced by grouping together the cell lines derived from the same human pathology, thus showing the total number of fusion events normalized by the number of cell lines composing a specific disease. The Virus Statistics panel shows the frequency of exogenous virus integration per human malignancy.
– "Gene Statistics": A word cloud diagram shows the most frequently recurring pGFEs supported by three methods.
– "Database Statistics": This subsection is composed of four panels. The first is related to data in the CCS (Fig.1B), the others relate only to FC and JA results. on this page, it is possible to get information about the number of pGFEs found in known databases (visualized as interactive Venn diagrams and in tabular fashion) and the distribution of predicted effects (histogram view).

**Dataset:** This page includes a description of the input dataset used for the analysis. Of the more than 1,000 samples available at the Broad Institute portal [16], we downloaded 935 PE RNA-seq samples in fastq format. The SE samples have been discarded since the software programs that were used required it. The histogram in this section shows the number of different cell lines derived from the same diseases (Fig.2B). Furthermore, starting from this section, it is possible to access web pages that resume cell line–specific details (e.g., COSMIC ID, drug resistance, and human disease).

**Downloads:** From this panel it is possible to download all the processed data described in this article (Fig.2D). Some of the files ("Summary Information" and "Viruses information") are specific products of the FusionCatcher algorithm.

**Table 2:** Example of possible queries on the LiGeA portal

Search by	Question	Query
Disease	What are the gene fusion events present in stomach adenocarcinoma cell lines?	Select "stomach adenocarcinoma" under the "disease" menu.
Cell line	What are the novel pGFEs affecting RH30(Sarcoma) cell line?	Select "RH30" under the "cell line" menu and check the box "show only novel results."
Chromosome	What are the most suitable fusion partners for chromosome 8?	Select "Chr8" either under the "5’ Chromosome" or the "3’ Chromosome" tab and leave the other forms blank.
Gene	How many human cell lines show the PML-RARA fusion event?	Select "PML" under the "5’ gene menu"; select "RARA" from the "3’ gene menu"; leave the "cell line" query form blank.
Fusion information	What are all the in-frame pGFEs in the Jurkat cell line?	Select "Jurkat" under the "cell line" menu; select "in-frame" under the "predicted effect" menu.
Fusion information	What are the known GFEs predicted to be in-frame in the Jurkat cell line?	Select "Jurkat" under the "cell line" menu; select '‘in-frame" under '‘predicted effect" menu; select "known" under the "fusion description" menu.
Algorithm	Show only those GFEs supported by FC and TF in RH30 cell line.	Select "RH30" under the "cell line" query form and check the boxes relative to FC and TF.
Viruses	Which cell lines are most affected by hepatitis C virus genome integration?	Select "hepatitis C virus" under the "virus" query form and leave the "cell line" query form blank.

## Availability of source code

- **Project name**: LiGeA: a comprehensive database of human gene fusion events
- **RRID:SCR_015940**
- **Project home page**: http://hpc-bioinformatics.cineca.it/fusion (GitHub project: https://github.com/tflati/fusion)
- **Operating system(s)**: Any
- **Programming language**: Python, JavaScript+HTML+CSS
- **Other requirements**: Django 1.10.5, Python 2.7.12, AngularJS 1.5.11
- **License**: GNU GPLv3

## Availability of supporting data

The datasets obtained and described within this article are freely downloadable at the LiGeA repository available at http://hpc-bioinformatics.cineca.it/fusion/downloads. Moreover, archival copies of processed files and the source code are available via the *GigaScience* database, GigaDB [38].

## Abbreviations

CCS: consensus call-set; CPU: central processing unit; ES: EricScript; FC: FusionCatcher; JA: JAFFA; LiGeA: cancer cell LInes GEne-fusions portAl; NGS: next-generation sequencing; pGFE: predicted gene fusion event; RAM: random access memory; RNA-seq: RNA sequencing; TCGA: Tumor Cancer Genome Atlas; TF: Tophat-Fusion.

## Competing interests

The authors declare that they have no competing interests.

## Funding

This work was supported by ELIXIR-IIB, CINECA, and Regione Lombardia. E.G. was supported by the Fondazione Italo Monzino and AIRC (award 17058) funding, awardee M.F..

M.F. was supported by ELIXIR-IIB-CINECA. S.G. was funded by ELIXIR-IIB, program name “Efficient implementation and distribution of HPC bioinformatics resources for Elixir scientific community,” (award 08/AR/2016-IBBE-BA). T.F. was funded by ELIXIR-IIB, program name “Efficient allocation of HPC bioinformatics resources through a federation of Galaxy web-based infrastructures” (award 05/AR/2016-IBBE-BA).

## Author contributions

T.C. and M.F. conceived and designed the work. All authors analyzed and interpreted data, wrote the manuscript, and approved the final manuscript.

## Supplementary Material

GIGA-D-17-00241_Original_Submission.pdf

GIGA-D-17-00241_Revision_1.pdf

GIGA-D-17-00241_Revision_2.pdf

GIGA-D-17-00241_Revision_3.pdf

ligea-chrome-audits-Reviewer_1_Report_(Original_Submission)-Attachment.jpg

Response_to_Reviewer_Comments_Original_Submission.pdf

Response_to_Reviewer_Comments_Revision_1.pdf

Response_to_Reviewer_Comments_Revision_2.pdf

Reviewer_1_Report_(Original_Submission) -- Mikhail Shugay 9/29/2017 Reviewed

Reviewer_1_Report_(Revision_1) -- Mikhail Shugay 1/15/2018 Reviewed

Reviewer_2_Report_(Original_Submission) -- Namshin Kim 10/1/2017 Reviewed

Reviewer_2_Report_(Revision_1) -- Namshin Kim 1/16/2018 Reviewed

Reviewer_3_Report_(Original_Submission) -- Daniel Nicorici 10/3/2017 Reviewed

Reviewer_3_Report_(Revision_1) -- Daniel Nicorici 1/29/2018 Reviewed
