# Supplementary material for: Massive NGS data analysis reveals hundreds of potential novel gene fusions in human cell lines
Source: Gigascience. 2018 Jun 1;7(10):giy062. doi: 10.1093/gigascience/giy062 (PMC6207142; doi:10.1093/gigascience/giy062)

# GigaScience

# Massive NGS Data Analysis Reveals Hundreds Of Potential Novel Gene Fusions in Human Cell Lines

--Manuscript Draft--

| | |
|---|---|
| **Manuscript Number:** | GIGA-D-17-00241 |
| **Full Title:** | Massive NGS Data Analysis Reveals Hundreds Of Potential Novel Gene Fusions in Human Cell Lines |
| **Article Type:** | Data Note |
| **Funding Information:** | |
| **Abstract:** | Background: Gene fusions derive from chromosomal rearrangements and the resulting chimeric transcripts are often endowed with oncogenic potential. Furthermore, they serve as diagnostic tools for the clinical classification of cancer subgroups with different prognosis and, in some cases, they can provide specific drug targets. So far, many efforts have been carried out to study gene fusion events occurring in tumor samples. In recent years, the availability of a comprehensive Next Generation Sequencing dataset for all the existing human tumor cell lines provides the opportunity to further investigate these data in order to identify novel and still uncharacterized gene fusion events.<br>Results: In our work, we have extensively reanalyzed 935 paired-end RNA-seq experiments downloaded from "The Cancer Cell Line Encyclopedia" repository, aiming at addressing novel cell-line specific gene fusion events. The bioinformatics analysis has been performed by the execution of three different gene fusion detection algorithms. The results have been further prioritized by running a bayesian classifier which makes an in silico validation. The collection of fusion events supported by all of the predictive softwares results in a robust set of ~ 2,000 in-silico predicted novel candidates suitable for downstream analyses. Given the huge amount of data produced, computational results have been collected in a database named LiGeA. The database can be browsed through a dynamical and interactive web portal, further integrated with validated data from other well known repositories. Taking advantage of the very intuitive query forms, the users can easily access, navigate, filter and select the putative gene fusions for further validations and studies. They can also find suitable experimental models for a given fusion of interest.<br>Conclusions: We believe that the LiGeA resource can represent not only the first compendium of both known and novel gene fusion events in the catalog of all of the human malignant cell lines, but it can also become a handy starting point for wet-lab biologists who wish to investigate novel cancer biomarkers and specific drug targets. |
| **Corresponding Author:** | Tiziana Castrignano', PhD<br>Cineca<br>Rome, Italy ITALY |
| **Corresponding Author Secondary Information:** | |
| **Corresponding Author's Institution:** | Cineca |
| **Corresponding Author's Secondary Institution:** | |
| **First Author:** | Silvia Gioiosa, PhD |
| **First Author Secondary Information:** | |
| **Order of Authors:** | Silvia Gioiosa, PhD |
| | Marco Bolis |
| | Tiziano Flati |
| | Annalisa Massini |
| | Enrico Garattini |
| | Giovanni Chillemi |
| | Maddalena Fratelli |

| | |
|---|---|
| | Tiziana Castrignano', PhD |
| **Order of Authors Secondary Information:** | |
| **Opposed Reviewers:** | |
| **Additional Information:** | |
| **Question** | **Response** |
| Are you submitting this manuscript to a special series or article collection? | No |
| **Experimental design and statistics**<br><br>Full details of the experimental design and statistical methods used should be given in the Methods section, as detailed in our Minimum Standards Reporting Checklist. Information essential to interpreting the data presented should be made available in the figure legends.<br><br>Have you included all the information requested in your manuscript? | Yes |
| **Resources**<br><br>A description of all resources used, including antibodies, cell lines, animals and software tools, with enough information to allow them to be uniquely identified, should be included in the Methods section. Authors are strongly encouraged to cite Research Resource Identifiers (RRIDs) for antibodies, model organisms and tools, where possible.<br><br>Have you included the information requested as detailed in our Minimum Standards Reporting Checklist? | Yes |
| **Availability of data and materials**<br><br>All datasets and code on which the conclusions of the paper rely must be either included in your submission or deposited in publicly available repositories (where available and ethically appropriate), referencing such data using a unique identifier in the references and in the “Availability of Data and Materials” section of your manuscript.<br><br>Have you have met the above requirement as detailed in our Minimum Standards Reporting Checklist? | Yes |

Powered by Editorial Manager® and ProduXion Manager® from Aries Systems Corporation

Manuscript
Click here to download Manuscript massive-ngs-data.pdf

*GigaScience*, 2017, 1–7

doi: xx.xxxx/xxxx
Manuscript in Preparation
Data Note

DATA NOTE

# Massive NGS Data Analysis Reveals Hundreds Of Potential Novel Gene Fusions in Human Cell Lines

Silvia Gioiosa[1,4,†], Marco Bolis[2,†], Tiziano Flati[1,4], Annalisa Massini[3], Enrico Garattini[2], Giovanni Chillemi[1], Maddalena Fratelli[2,*] and Tiziana Castrignanò[1,*]

[1]SCAI-Super Computing Applications and Innovation Department, CINECA, Rome, Italy, and [2]Laboratory of Molecular Biology, IRCCS-Istituto di Ricerche Farmacologiche "Mario Negri," Milano, Italy, and [3]Computer Science Department, Sapienza University of Rome, Italy, and [4]National Council of Research, CNR, Institute of Biomembranes, Bioenergetics and Molecular Biotechnologies, Bari, Italy.

[†]Contributed equally.
[*] To whom correspondence should be addressed: t.castrignano@cineca.it; maddalena@marionegri.it

## Abstract

**Background**: Gene fusions derive from chromosomal rearrangements and the resulting chimeric transcripts are often endowed with oncogenic potential. Furthermore, they serve as diagnostic tools for the clinical classification of cancer subgroups with different prognosis and, in some cases, they can provide specific drug targets. So far, many efforts have been carried out to study gene fusion events occurring in tumor samples. In recent years, the availability of a comprehensive Next Generation Sequencing dataset for all the existing human tumor cell lines provides the opportunity to further investigate these data in order to identify novel and still uncharacterized gene fusion events.

**Results**: In our work, we have extensively reanalyzed 935 paired-end RNA-seq experiments downloaded from "The Cancer Cell Line Encyclopedia" repository, aiming at addressing novel cell-line specific gene fusion events. The bioinformatics analysis has been performed by the execution of three different gene fusion detection algorithms. The results have been further prioritized by running a bayesian classifier which makes an *in silico* validation. The collection of fusion events supported by all of the predictive softwares results in a robust set of ~ 2,000 *in-silico* predicted novel candidates suitable for downstream analyses. Given the huge amount of data produced, computational results have been collected in a database named LiGeA. The database can be browsed through a dynamical and interactive web portal, further integrated with validated data from other well known repositories. Taking advantage of the very intuitive query forms, the users can easily access, navigate, filter and select the putative gene fusions for further validations and studies. They can also find suitable experimental models for a given fusion of interest.

**Conclusions**: We believe that the LiGeA resource can represent not only the first compendium of both known and novel gene fusion events in the catalog of all of the human malignant cell lines, but it can also become a handy starting point for wet-lab biologists who wish to investigate novel cancer biomarkers and specific drug targets.

**Key words**: Database; Human gene fusions; Cell Lines; NGS; Gene Fusion detection algorithms; Chromosomal rearrangements

**Compiled on:** September 22, 2017.
Draft manuscript prepared by the author.

**Key Points**

- A massive bioinformatics analysis conducted on Paired-End RNA-seq samples from 935 Human Cell Lines reveals a landscape of known and novel *in-silico* predicted gene fusion events;
- LiGeA Portal represents a user-friendly database for the visualization and interrogation of the results;
- LiGeA Portal is further integrated with information from other databases and with gene-fusion priotirization analysis, in order to address targeted experimental validations on a highly reliable set of candidate fusions.

## Background

Gene fusion events result from chromosomal rearrangements which lead to the juxtaposition of two previously separated genes. The accidental joining of DNA of two genes can generate hybrid proteins. It can also result in the misregulation of the transcription of one gene by the *cis-regulatory* elements (promoters or enhancers) of another, sometimes resulting in the production of oncoproteins that bring the cell to a neoplastic transformation (Mitelman et al.; 2007). Not only gene fusions can have a strong oncogenic potential (Mertens et al.; 2015), but they also serve as diagnostic tools for the clinical classification of cancer subgroups with different prognosis and, in some cases, they may provide specific drug targets (Serratì et al.; 2016). For instance, the presence of the PLM-RARA fusion product is a specific hallmark of acute promyelocytic leukemia (APL) (Borrow et al.; 1990) and represents the first example of gene-fusion targeted therapy (Nervi et al.; 1998) that has changed the natural history of this disease. Hence, there are several reasons why studying gene fusions in cancer is very important. In recent years, Next-Generation Sequencing (NGS) technologies have played an essential role in the understanding of the altered genetic pathways involved in human cancers. Nowadays, most of the studies aiming at fusion discovery use NGS techniques followed by massive bioinformatics analyses. The greatest challenge of these sophisticated algorithms of prediction is the ability to discriminate between artifacts and really occurring chromosomal rearrangements (Lou et al.; 2009). Moreover, each gene fusion predicting software differs in terms of sensitivity and specificity. For this reason, a combination of several tools can overcome these limits and define a reliable call-set of truly occurring chromosomal rearrangements (Mertens et al.; 2015). In the last decade, much effort has been done to catalog gene fusion events, thus resulting in a wide production of databases. At present, a dozen of published databases regarding oncogenic fusion genes exists (see table 1 for a summary). Some of them (e.g. FusionCancer, ChiTaRS-3.1) collect *in silico* predictions of chimeric genes, obtained analyzing publicly available datasets derived from heterogeneous sources either in terms of experimental material (a mix of Single-End and Paired-End RNA-seq data, ESTs) and in terms of data source (patients and cell lines). Some others collect gene fusion events with experimental evidences manually curated from literature collection (e.g. TCGA, Mitelman, TICdb, COSMIC, ONGene). In this work we focused on the whole catalog of Human Cell Lines, thus obtaining a homogeneous input NGS dataset covering several human malignancies. We exerted an extensive bioinformatics analysis of 935 paired-end RNA-seq samples derived from 22 different tumor tissues and used a combination of the best performing gene fusion-detecting algorithms. For ease of understanding, we define the predicted Gene Fusion Event (pGFE) as the entity constituted by the gene fusion couple in a specific cell line and designate the Consensus Call-set (CCS) as the number of pGFEs supported by all the used algorithms. Starting from this assumption, we obtained a total of 990,627 pGFE, 3,294 of which belonging to the CCS. Moreover, since not all the pGFEs can give rise to oncogenic transformations, the use of a priotirization software is recommended in order to distinguish between real driver mutations from passenger ones. Therefore, a robust Bayesian classifier has been used to perform an *in silico* validation of the results. Since one of the main purposes of our extensive big data analysis is encouraging the reuse of our results in order to experimentally validate the *in-silico* predictions, we set up a web portal collecting these data, LiGeA (cancer cell LInes Gene fusion portAl). It is possible to browse, search and freely download all the results obtained and described within this article at the LiGeA repository web page available at http://hpc-bioinformatics.cineca.it/fusion/. To our knowledge, our resource represents the first compendium of known and predicted novel gene fusion events in cell lines from 22 different human tumor types.

## Data Description

### Methods

We have analyzed 935 paired-end RNA-seq experiments available at the Cancer Cell Line Encyclopedia repository, for a total of 32 TB of input raw data. The analysis has been carried out by using three different somatic fusion gene detection algorithms: FusionCatcher (Nicorici et al.; 2014), EricScript (Benelli et al.; 2012) and Tophat-Fusion (Daehwan and Salzberg; 2011). Indeed, a recent assessment of methods for the fusion transcripts detection from RNA-Seq data, highlighted these three algorithms as the top performing ones using hg38 as the reference genome (Kumar et al.; 2016). Furthermore, these softwares give several layers of information in their output files, thus giving us the opportunity to collect and interconnect a wide set of additional data for each pGFE. Here is a short description of each fusion detection tool, accompanied by the versions and the used parameters.

- **FusionCatcher (FC)**: FC is a Python based algorithm. It executes a first mapping run with Bowtie v.1.2.0 (Langmead et al.; 2009) and then performs the Gene fusion detection basing on three different aligners: Bowtie2 v.2.2.9 (Langmead and Salzberg; 2012), BLAT v.36 (Kent; 2002) and STAR v.2.5.2b (Dobin et al.; 2013). FC takes advantage of NCBI Viral Genomes (v. 2016-01-06) in order to detect exogenous virus material integration into the host genome. Moreover, the FC algorithm compares its own output with a set of published databases, thus proving a detailed list of truly positive and false positive pGFE candidates. In our analysis we downloaded FC v. 0.99.5a and Ensembl genome annotation v.83 and used hg38/GRCh38 as genome assembly version. The software was executed with default parameters, requiring 111,620 CPU core hours, 125 GB of RAM and 20 CPUs to complete the execution on our input dataset. Overall, FC detected 49,032 pGFEs involving 11,157 genes. Interestingly, 978 pGFEs (68% of novel pGFEs in the CCS), have an 'in-

**Table 1.** State of the art of databases reporting gene fusions

| Database Name | URL | Short Description |
|---|---|---|
| Tumor Fusion Gene Data Portal | http://54.84.12.177/PanCanFusV2// | A collection of fusion genes in the Tumor Cancer Genome Atlas (TCGA) samples. |
| TICdb (Novo et al.; 2007) | http://www.unav.es/genetica/TICdb/ | A collection of 1,374 fusion sequences extracted either from public databases or from published papers (last update: 2013). |
| chimerDB3.0 (Lee et al.; 2017) | http://203.255.191.229:8080/chimerdbv31/mindex.cdb | A catalog of fusion genes encompassing analysis of TCGA data and manual curations from literature. |
| ONGene (Liu et al.; 2017) | http://ongene.bioinfo-minzhao.org/ | Literature-derived database of oncogenes |
| COSMIC Cell Lines | http://cancer.sanger.ac.uk/cell_lines | Gene fusions are manually curated from peer reviewed publications. Currently COSMIC includes information on fusions involved in solid tumors but not yet leukemias and lymphomas. |
| Mitelman (Mitelman et al.; 2007) | https://cgap.nci.nih.gov/Chromosomes/Mitelman | Reports hundreds of gene fusions associated with clinical reports but does not contain sequence data. |
| ChiTaRs-3.1 (Gorohovski et al.; 2017) | http://chitars.md.biu.ac.il/index.html | A collection of 34,922 chimeric transcripts identified by Expressed Sequence Tags (ESTs) and mRNAs from the GenBank, ChimerDB, dbCRID, TICdb and the Mitelman collection of cancer fusions for several organisms. |
| FusionCancer (Wang et al.; 2015) | http://donglab.ecnu.edu.cn/databases/FusionCancer/ | 591 samples, both single-end and paired-end RNA-seq, published on SRA (http://www.ncbi.nlm.nih.gov/sra) database between 2008 and 2014 covering 15 kinds of human cancers . |

frame' predicted effect and have never been described before, either in other databases or in the literature.

- **EricScript (ES)**: ES is developed in R (R Development Core Team; 2008), perl and bash scripts. It uses the BWA aligner (Li and Durbin; 2009) to perform the mapping on the transcriptome reference and samtools v. 0.1.19 (Li et al.; 2009) to handle with SAM/BAM files. Recalibration of the exon-junction reference is performed by using BLAT (Kent; 2002). For the purposes of this project, BLAT v.36 was downloaded at http://genome-test.cse.ucsc.edu/~kent/exe/linux/. Moreover, it was necessary to download R v.3.3.1 and a bedtools version greater than 2.20 (here we used v. 2.24). For this study, ES version 0.5.5 was obtained at https://sourceforge.net/projects/ericscript/files/. The Ensembl Database v. 84 was downloaded from https://docs.google.com/uc?id=0B9s__vuJPvIiUGt1SnFMZFg4TlE&export=download and built locally using BWA software with the command:

  ```
  bwa index -a bwtsw allseq.fa
  ```

  A total amount of 130,900 CPU core hours, 125 GB of RAM and 20 CPUs was required to successfully complete the analysis. ES final results were reported in 2 files. Only predicted gene fusion products present in 'file.results.filtered.csv' have been considered for subsequent analysis. Overall, ES detected 929,638 pGFEs involving 17,117 genes.
- **Tophat-Fusion (TF)**: TF uses the Tophat-fusion-post function in order to create a filtered list of gene fusion candidates, starting from the output files obtained running Tophat with the "-fusion-search" option (Trapnell et al.; 2009)." The following commands were run subsequently:

  ```
  tophat -o $Sample.output/ -p 20 -fusion-search -keep-fasta-order -bowtie1 -no-coverage-search -r 160 -mate-std-dev 34 -max-intron-length 100000 -fusion-min-dist 100000 -fusion-anchor-length 13 $BOWTIE_INDEX/hg38 $Sample_1.fastq $Sample_2.fastq
  ```

  ```
  cd $Sample.output/
  ```

  ```
  tophat-fusion-post -p 20 -skip-blast $BOWTIE_INDEX/hg38
  ```

  Tophat-2.0.12 and samtools 0.1.19 versions were used for this study. This algorithm turned out to be the slowest of the three ones, taking about 200,000 CPU core hours, 20 CPUs and 125 GB of RAM in order to complete its runs on the whole input dataset. TF produces several output files but only the file named "results.txt", representing the filtered list of predicted gene fusions, was used for subsequent analysis. The results encompassing "Chromosome M" have been manually discarded from the final results, *in primis* because TF was the only one of the three algorithms reporting it, secondly because they represented *bona-fide* false positive outcomes. Overall, TF highlighted 34,202 pGFEs involving 7,819 genes.

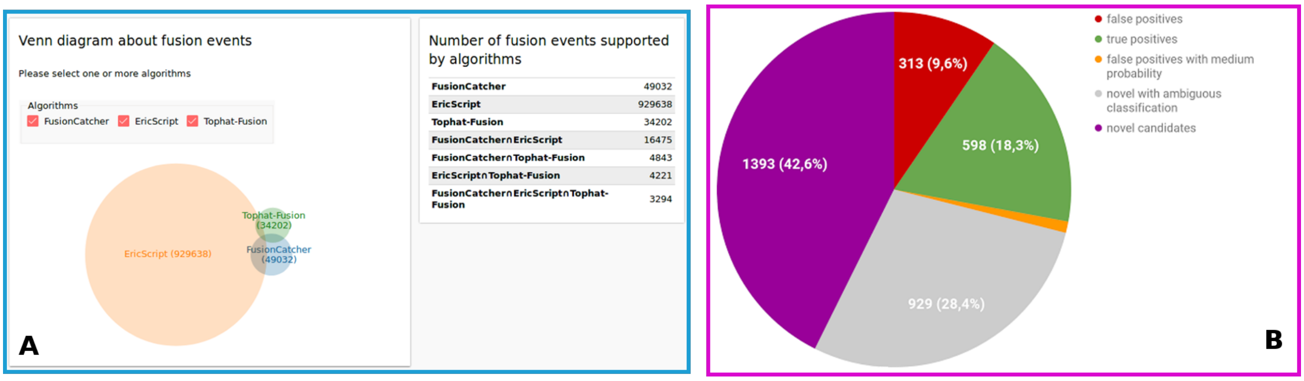


**Figure 1.** a) Venn diagram showing the intersection of the pGFEs identified by the three algorithms. b) Distribution of pGFEs in the Consensus Call-set: 42% (purple) of the CCS has not been previously described in any other database or scientific publication; 9.6% (red) and 18% of the CCS have been reported in databases from healthy/tumoral samples thus representing the false/true positive subset of our analysis; 1% of the CCS (orange) reports tags which classify the pGFE as a false positive couple with medium probability; 28.4% (grey) of the results represent novel pGFEs tagged with values which classify them as both false and true positives.

## Data Statistics and Validation

Overall, our extensive analysis predicted a CCS of 3,294 pGFEs and respectively 15,657/971,676 pGFEs supported by two/one methods (Fig. 1A). As a validation of our analysis, 95% of the genes known to be functionally implicated in cancer and collected under COSMIC gene census, are present in our final dataset. As a further validation of our results, about 1/5 of our CCS has already been published or is present in the following databases: chimerdb3; ONGene; COSMIC; tcga; ticdb. Finally, only a small subset of the pGFEs (~9% of data) present in the CCS have been recognized as false positive predictions, thus supporting the idea that a combination of algorithms can be of great utility in order to increase the sensitivity and the specificity of the tests. It is worth mentioning that, not only our analysis confirmed a large number of known fusion events, but it also highlighted 2,322 novel putative pGFEs in the CCS which could undergo further downstream analysis (Fig.1B). Therefore, a further step of analysis was run with Oncofuse v.1.1.1 (Shugay et al.; 2013) in order to distinguish driver mutations (genomic abnormalities responsible for cancer) from passenger mutations (inert somatic mutations not implicated in carcinogenesis). Oncofuse is considered an *in silico* validation post-processing step which prioritizes the results obtained from each of the three algorithms. It assigns a functional prediction score to each putative fusion sequence breakpoint identified by the three softwares thus hinting which pGFEs are worthy of being experimentally validated. Oncofuse supports multiple input formats such as the output from TF and FC. In order to run it also on the outputs from ES, a short pre-processing step was executed on these data. As suggested on Oncofuse manual, the accepted default input format is a tab-delimited file with lines containing 5' and 3' breakpoint positions. Therefore, these columns were extracted from ES output files and redirected into Oncofuse accepted input file. Oncofuse was run with default parameters using hg38 as the reference genome.

## Availability of supporting data and materials

The datasets obtained and described within this article are freely searchable and downloadable at the LiGeA repository available at `http://hpc-bioinformatics.cineca.it/fusion/downloads`.

## Database Description

LiGeA is a database server based on graph-db technology (Neo4j). The portal stores all of the results obtained from each fusion gene predicting algorithm and the priorit ization analysis outcome. Anyway, this database contains not only a mere collection of *in silico* predictions. Indeed, it has been integrated with other useful external resources in order to offer a carefully-curated web compendium. Here is a short list of the added features:

- Whenever the gene fusion couple has already been experimentally validated and published, an extra column with COSMIC icon is added to the results. By clicking on it, the user will be redirected to an external link containing a manually-curated catalog of 212 literature-derived somatic mutations in cancer (COSMIC; 2017a);
- Cancer Gene Census is a manually curated catalog of 616 genes for which mutations have been causally implicated in oncogenesis (Futreal et al.; 2004)). Whenever one of the two genes involved in the pGFE has been already described to be implicated in cancer, the gene is tagged with a "sanger" icon. By clicking on it, an external link to the Cancer Gene Census is provided showing a complete gene view (COSMIC; 2017b).
- A legend based on a colorful signature has been added to tag the FC predictions as 'validated truly positive couples' (green circle), 'validated false positive couples' (red circle), 'false positive couples with medium probability' (orange circle) and 'ambiguous signature' because tagged with both positive and negative values (grey circle) ;
- A functional prediction score obtained by extensively running the Oncofuse software, is reported as additional tag to each of the three algorithm outputs.

LiGeA portal is divided into several sections which allow a user-friendly navigation.

- **Home:** In the homepage, the user is provided with a quick overview of the database. A global summary table reports a numeric recapitulation (e.g. the number of genes/transcripts/exons collected into the portal; the number of predicted proteins and so on). Moreover, a histogram shows an abstract of the top 50 involved cell lines. By moving the cursor on the bars, a pop-up opens showing the cell line name and the corresponding number of the unique fusion events predicted by all the algorithms. Information about the algorithm predictions hosted into the portal are

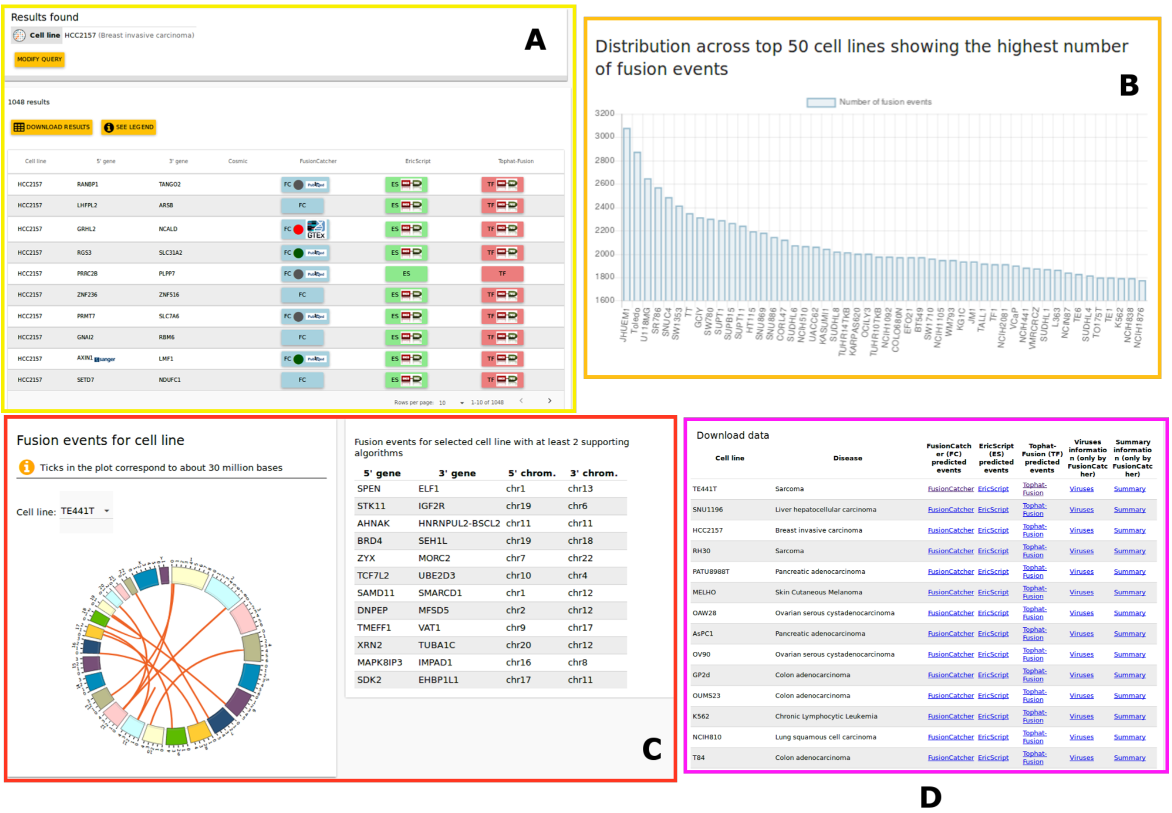


**Figure 2.** An overview of LiGeA portal. a) An example ’search by Disease’ and the corresponding output; b) An overview of the input dataset; c) A circos diagram showing the graphical outcome of a ’query by cell line’ and the corresponding related table; d) An extract from the ’download’ page.

supplied with an interactive Venn Diagram linked to a dynamical table. Upon user selection of the algorithm/s of interest, both the diagram and the table refresh thus showing the resulting number of intersections.

- **Search:** This utility allows several searching options to browse and mine genomic-fusion events stored in LiGeA portal (see table 2 for an overview). All the resulting outputs are sorted by the number of algorithms supporting the fusion events, thus showing on the top of the table the most robust set of results. As added feature, when specifying the features of interest, it is also possible to choose the number of predicting algorithms. The obtained results can be downloaded in a tabular format. Two out of nine of the query forms (’search by fusion information’ and ’search by virus’) are specific annotations derived FC algorithm. Here is a short description of the provided searching utilities.
- ’Search by Disease’: In this section, all the cell lines derived from the same disease have been grouped together. In this way, it is possible to navigate the gene fusions putatively causing specific malignancies. The number of the cell lines constituting the queried subset is shown besides the pathology name (Fig.2A).
- ’Search by Cell Line’: This module allows to navigate the database by indicating a specific cell line name. It is possible to tune the results by showing only the novel predictions not yet described in any other database or publication.
- ’Search by Chromosome’: This query can be performed by inserting one or two chromosomes involved in the fusion event. The cell line name can be either indicated or not.
- ’Search by Gene’: the user can select up to two gene names (Gene Symbol or ENSEMBL ID) and the ’cell line’ form can be either selected or not. The genes reported in the query form are black if they are involved in pGFE and gray if they are not.
- ’Search by Transcript’: Since the same gene can give rise to different transcripts, it could be reasonable to query which of the transcripts produced by a specific gene are affected by a fusion event. This kind of query can be satisfied by inserting the Ensembl Transcript (ENST) IDs in the specific form.
- ’Search by Exon’: Some of the queries allow to go much more into molecular detail. This search can be done by inserting one or two exon IDs involved in the fusion event. The cell line name can be either indicated or not. In this way it is possible to highlight the specific exons which turn out to be fused in the final result.
- ’Search by Fusion information’: The pGFEs may have different predicted effects. Indeed, depending on the location of the chromosomal break points, the resulting protein may be in-frame, out-of frame, truncated and so on. Since the selectable values present in the fusion information form are specific of FC algorithm, the result of this query returns a table without ES and TF data. We suggest to view this section of FC manual https://github.com/ndaniel/fusioncatcher/blob/master/doc/manual.md#62---output-data-output-data in order to obtain a full description of all of the tags.
- ’Search by Algorithm’: this type of query is suitable for users who wish to navigate the outputs from specific softwares, choosing them individually or in combination.
- ’Search by Viruses’: Another useful information retrievable from the database regards virus sequence integration into the host genome. This search utility is virus-centered since it is possible to indicate or not the host cell line name. It is possible to select the virus name of interest (whether using GI ID or NC ID). Furthermore, a clickable link redirecting to the virus genome is also shown on the right of the table.

- **Statistics:** this section allows a visual inspection of the re-

**Table 2.** Example of possible queries on LiGeA portal

| Search by | Question | Query |
|---|---|---|
| Disease | 'what are the fusion events present in stomach adenocarcinoma cell lines?' | Select 'stomach adenocarcinoma' under 'disease' menu |
| Cell Line | 'what are the novel putative pGFEs affecting RH30(Sarcoma) cell line?' | Select 'RH30' under the cell line menu and check the box 'show only novel results' |
| Chromosome | 'what are the most suitable fusion partners for chromosome 8?' | Select 'Chr8' either under the '5' Cromosome' or under the '3' Chromosome' tab and leave blank the other forms |
| Gene | 'how many human cell lines show the PML-RARA fusion event?' | Select 'PML' under the '5' gene menu'; Select 'RARA' from the '3' gene menu'; leave blank the 'Cell Line' query form; |
| Fusion information | 'what are all the pGFEs predicted to be in-frame in Jurkat cell line?' | select 'Jurkat' under 'Cell line' menu; Select 'in-frame' under 'predicted effect menu |
| Fusion information | 'what are the known GFE predicted to be in-frame in Jurkat cell line?' | Select 'Jurkat' under 'Cell line' menu; Select 'in-frame' under 'predicted effect menu; select 'cell_line' under 'Fusion description' menu; |
| Algorithm | 'show me only those GFE supported by FC and TF in RH30 cell line' | Select 'RH30' under 'Cell Line' query form and check the boxes relative to FC and TF; |
| Viruses | 'which cell lines are most affected by Hepatitis C virus genome integration?' | Select 'Hepatitis C virus' under 'Virus' query form and let blank the 'Cell line' query form; |

sults. The four sub-menus are organized as follows:

- 'Cell Line Statistics': by choosing the Cell Line of interest, the resulting circular diagram shows all the chromosome couples involved in GFE predicted by at least two algorithms. The table on the right summarizes the resulting couples of the genes and chromosomes (Fig. 2C).
- 'Chromosome Statistics': this page reports a dynamical pie-chart showing the number of fusion events per human chromosome; by clicking on each slice of the pie, the related table automatically updates showing a chromosome summary statistics. Furthermore, information about the number of inter- and intra-chromosomal rearrangements detected by each algorithm is also reported.
- 'Disease Statistics': The 'Fusion Statistics' pie-chart was produced by grouping together the cell lines derived from the same human pathology thus showing the total number of fusion events normalized by the number of cell lines composing a specific disease. The 'Virus statistics panel' shows the frequency of exogenous virus integration per human malignancy.
- 'Gene Statistics': A word cloud diagram showing the most recurring pGFEs supported by three methods.
- 'Database Statistics': This sub-section is composed by four panels, the first regarding data in the CCS (Fig. 1B), the others relating only to FC results. In this page it is possible to get information about the number of pGFEs found in known databases (visualized as interactive Venn diagrams and tabular fashion) and the distribution of predicted effects (histogram view).

- **Dataset:** This page is a description of the input dataset used for the analysis. Among the above 1000 samples available at Broad institute portal CCLE repository, we downloaded 935 PE RNA-seq in fastq format. The SE samples have been discarded since the used softwares required it. The histogram in this section shows the number of the different cell lines derived from the same diseases (Fig. 2B).

- **Downloads:** From this panel it is possible to download all the processed data described within this article (Fig. 2D). Some of the files ('Summary information' and 'Viruses information') are specific products of FusionCatcher algorithm.

## Declarations

### List of abbreviations

LiGeA: cancer cell LInes GEne-fusions portAl; pGFE: predicted Gene Fusion Event; NGS: Next Generation Sequencing; TCGA: Tumor Cancer Genome Atlas; SRA : Sequence Read Archive; APL: acute promielocytic leukemia; CCS: Consensus Call-Set FC: FusionCatcher; ES: EricScript; TF: Tophat-Fusion;

### Consent for publication

'Not applicable'

### Competing Interests

The authors declare that they have no competing interests.

## Funding

This work was supported by ELIXIR-IIB, CINECA and Regione Lombardia.

## Author's Contributions

TC and MF conceived and designed the work. All authors analyzed, interpreted data, wrote the manuscript and approved the final manuscript.

## Acknowledgements

We acknowledge Andrea Micco for his useful tests on the first prototype of the system. We acknowledge the CINECA and the Regione Lombardia award under the LISA initiative 2016-2018, for the availability of high performance computing resources and support.

## References

Benelli, M., Pescucci, C., Marseglia, G., Severgnini, M., Torricelli, F. and Magi, A. (2012). Discovering chimeric transcripts in paired-end rna-seq data by using ericscript, *Bioinformatics* **28**(24): 3232–3239.
**URL:** *+ http://dx.doi.org/10.1093/bioinformatics/bts617*

Borrow, J., Goddard, A., Sheer, D. and Solomon, E. (1990). Molecular analysis of acute promyelocytic leukemia breakpoint cluster region on chromosome 17, *Science* **249**(4976): 1577–1580.
**URL:** *http://science.sciencemag.org/content/249/4976/1577*

COSMIC (2017a). Cosmic database-wellcome trust sanger institute @ONLINE.
**URL:** *http://cancer.sanger.ac.uk/cosmic*

COSMIC (2017b). Cosmic gene census - wellcome trust sanger institute @ONLINE.
**URL:** *http://cancer.sanger.ac.uk/census*

Daehwan, K. and Salzberg, S. (2011). Tophat-fusion: An algorithm for discovery of novel fusion transcripts, *Genome Biology* **12**(8).

Dobin, A., Davis, C. A., Schlesinger, F., Drenkow, J., Zaleski, C., Jha, S., Batut, P., Chaisson, M. and Gingeras, T. R. (2013). Star: ultrafast universal rna-seq aligner, *Bioinformatics* **29**(1): 15–21.
**URL:** *+ http://dx.doi.org/10.1093/bioinformatics/bts635*

Futreal, P. A., Coin, L., Marshall, M., Down, T., Hubbard, T., Wooster, R., Rahman, N. and Stratton, M. R. (2004). A census of human cancer genes., *Nature reviews Cancer* **4**(3): 177–183.

Gorohovski, A., Tagore, S., Palande, V., Malka, A., Raviv-Shay, D. and Frenkel-Morgenstern, M. (2017). Chitars-3.1—the enhanced chimeric transcripts and rna-seq database matched with protein–protein interactions, *Nucleic Acids Research* **45**(D1): D790–D795.
**URL:** *+ http://dx.doi.org/10.1093/nar/gkw1127*

Kent, W. (2002). Blat—the blast-like alignment tool., *Genome Research* **12**(4): 656–664.

Kumar, S., Vo, A. D., Qin, F. and Li, H. (2016). Comparative assessment of methods for the fusion transcripts detection from rna-seq data, *Nature Scientific Reports* **6**.

Langmead, B. and Salzberg, S. (2012). Fast gapped-read alignment with bowtie 2., *Nature methods* **9**(4): 357–359.

Langmead, B., Trapnell, C., Pop, M. and Salzberg, S. L. (2009). Ultrafast and memory-efficient alignment of short dna sequences to the human genome, *Genome Biology* **10**(3): R25.
**URL:** *https://doi.org/10.1186/gb-2009-10-3-r25*

Lee, M., Lee, K., Yu, N., Jang, I., Choi, I., Kim, P., Jang, Y. E., Kim, B., Kim, S., Lee, B., Kang, J. and Lee, S. (2017). Chimerdb 3.0: an enhanced database for fusion genes from cancer transcriptome and literature data mining, *Nucleic Acids Research* **45**(D1): D784–D789.
**URL:** *+ http://dx.doi.org/10.1093/nar/gkw1083*

Li, H. and Durbin, R. (2009). Fast and accurate short read alignment with burrows–wheeler transform, *Bioinformatics* **25**(14): 1754–1760.
**URL:** *+ http://dx.doi.org/10.1093/bioinformatics/btp324*

Li, H., Handsaker, B., Wysoker, A., Fennell, T., Ruan, J., Homer, N., Marth, G., Abecasis, G. and Durbin, R. (2009). The sequence alignment/map format and samtools, *Bioinformatics* **25**(16): 2078–2079.
**URL:** *http://dx.doi.org/10.1093/bioinformatics/btp352*

Liu, Y., Sun, J. and Zhao, M. (2017). Ongene: A literature-based database for human oncogenes, *Journal of Genetics and Genomics* **44**(2): 119 – 121.
**URL:** *http://www.sciencedirect.com/science/article/pii/S1673852716302053*

Lou, D. I., Hussmann, J. A., McBee, R. M., Acevedo, A., Andino, R., Press, W. H. and Sawyer, S. L. (2009). High-throughput dna sequencing errors are reduced by orders of magnitude using circle sequencing, *Proceedings of the National Academy of Sciences of the United States of America* **110**(49): 19872–19877.

Mertens, F., Johansson, B., Fioretos, T. and Mitelman, F. (2015). The emerging complexity of gene fusions in cancer, *Nat Rev Cancer* **15**(6).

Mitelman, F., Johansson, B. and Mertens, F. (2007). "the impact of translocations and gene fusions on cancer causation", *Nat Rav Cancer* **7**(4): 233 – 245.

Nervi, C., Ferrara, F. F., Fanelli, M., Rippo, M. R., Tomassini, B., Ferrucci, P. F., Ruthardt, M., Gelmetti, V., Gambacorti-Passerini, C., Diverio, D., Grignani, F., Pelicci, P. G. and Testi, R. (1998). Caspases mediate retinoic acid–induced degradation of the acute promyelocytic leukemia pml/rar$\alpha$ fusion protein, *Blood* **92**(7): 2244–2251.
**URL:** *http://www.bloodjournal.org/content/92/7/2244*

Nicorici, D., Satalan, M., Edgren, H., Kangaspeska, S., Murumagi, A., Kallioniemi, O., Virtanen, S. and Kilkku, O. (2014). Fusioncatcher - a tool for finding somatic fusion genes in paired-end rna-sequencing data, *bioRxiv* .
**URL:** *http://www.biorxiv.org/content/early/2014/11/19/011650*

Novo, F., de Mendíbil, I. and Vizmanos, J. (2007). Ticdb: a collection of gene-mapped translocation breakpoints in cancer., *BMC Genomics* **8**(33).

R Development Core Team (2008). *R: A Language and Environment for Statistical Computing*, R Foundation for Statistical Computing, Vienna, Austria. ISBN 3-900051-07-0.
**URL:** *http://www.R-project.org*

Serratì, S., De Summa, S., Pilato, B., Petriella, D., Lacalamita, R., Tommasi, S. and Pinto, R. (2016). Next-generation sequencing: advances and applications in cancer diagnosis., *OncoTargets and Therapy* **9**: 7355–7365.

Shugay, M., Ortiz de Mendíbil, I., Vizmanos, J. L. and Novo, F. J. (2013). Oncofuse: a computational framework for the prediction of the oncogenic potential of gene fusions, *Bioinformatics* **29**(20): 2539–2546.
**URL:** *+ http://dx.doi.org/10.1093/bioinformatics/btt445*

Trapnell, C., Pachter, L. and Salzberg, S. L. (2009). Tophat: discovering splice junctions with rna-seq, *Bioinformatics* **25**(9): 1105–1111.
**URL:** *+ http://dx.doi.org/10.1093/bioinformatics/btp120*

Wang, Y., Wu, N., Liu, J., Wu, Z. and Dong, D. (2015). Fusioncancer: A database of cancer fusion genes derived from rna-seq data, **10**: 131.

Click here to download Figure figura2.png

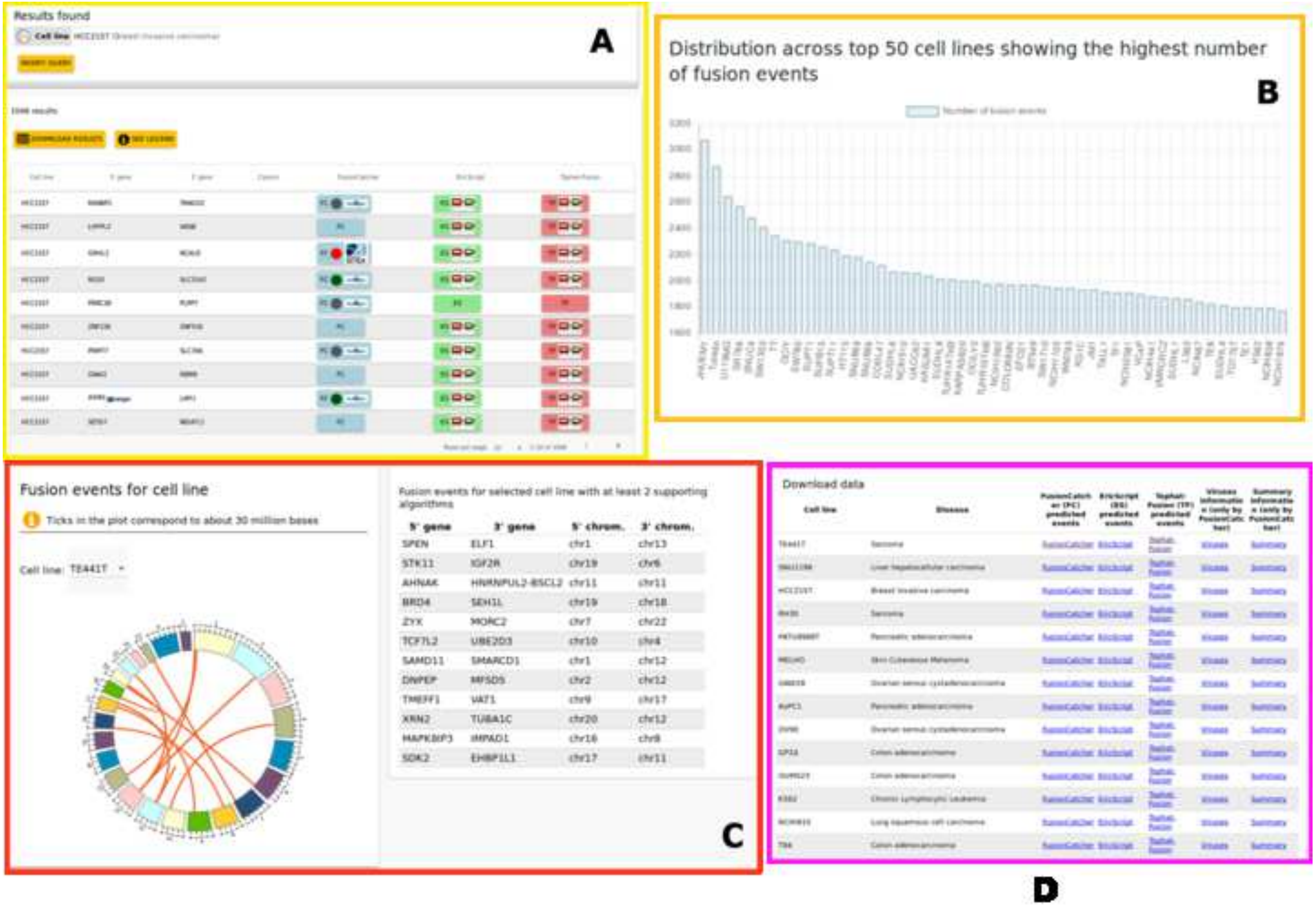

Click here to download Figure figura1.png

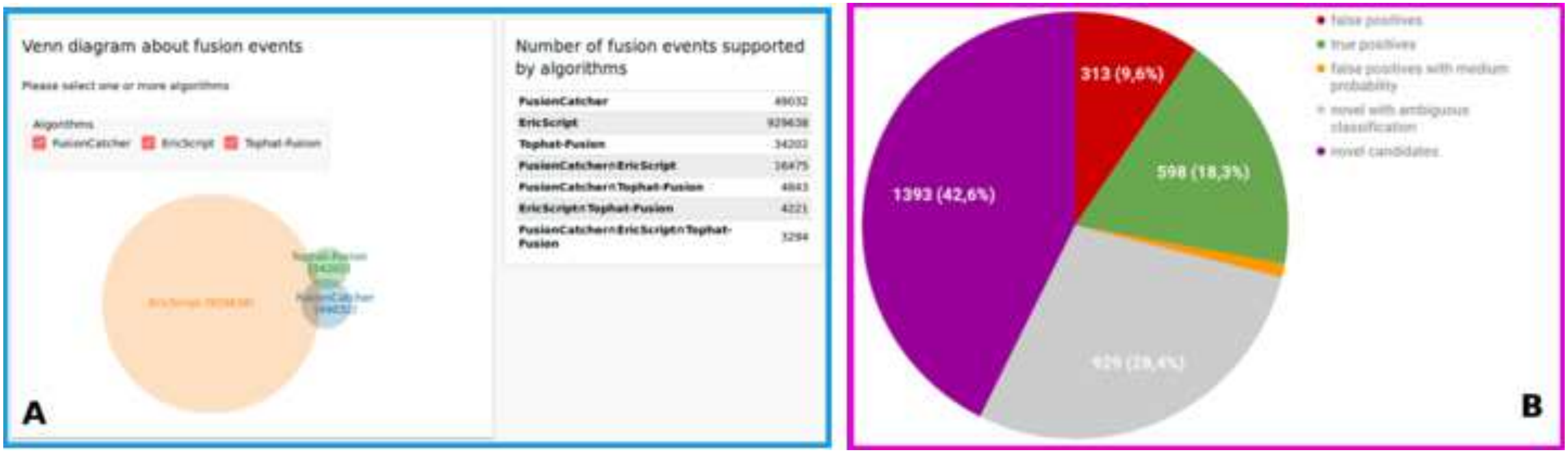

Supplement: GIGA-D-17-00241_Original_Submission.pdf [file giy062_giga-d-17-00241_original_submission.pdf]
