# Supplementary material for: Massive NGS data analysis reveals hundreds of potential novel gene fusions in human cell lines
Source: Gigascience. 2018 Jun 1;7(10):giy062. doi: 10.1093/gigascience/giy062 (PMC6207142; doi:10.1093/gigascience/giy062)
Supplement: GIGA-D-17-00241_Revision_1.pdf [file giy062_giga-d-17-00241_revision_1.pdf]

GigaScience

Massive NGS Data Analysis Reveals Hundreds Of Potential Novel Gene Fusions in Human Cell Lines

--Manuscript Draft--

**Manuscript Number:**	GIGA-D-17-00241R1
**Full Title:**	Massive NGS Data Analysis Reveals Hundreds Of Potential Novel Gene Fusions in Human Cell Lines
**Article Type:**	Data Note
**Funding Information:**	
**Abstract:**	Background: Gene fusions derive from chromosomal rearrangements and the resulting chimeric transcripts are often endowed with oncogenic potential. Furthermore, they serve as diagnostic tools for the clinical classification of cancer subgroups with different prognosis and, in some cases, they can provide specific drug targets. So far, many efforts have been carried out to study gene fusion events occurring in tumor samples. In recent years, the availability of a comprehensive Next Generation Sequencing dataset for all the existing human tumor cell lines has provided the opportunity to further investigate these data in order to identify novel and still uncharacterized gene fusion events.<br>Results: In our work, we have extensively reanalyzed 935 paired-end RNA-seq experiments downloaded from "The Cancer Cell Line Encyclopedia" repository, aiming at addressing novel putative cell-line specific gene fusion events. The bioinformatics analysis has been performed by the execution of three different gene fusion detection algorithms. The results have been further prioritized by running a bayesian classifier which makes an in silico validation. The collection of fusion events supported by all of the predictive softwares results in a robust set of ~ 2,000 in-silico predicted novel candidates suitable for downstream analyses. Given the huge amount of data produced, computational results have been collected in a database named LiGeA. The database can be browsed through a dynamical and interactive web portal, further integrated with validated data from other well known repositories. Taking advantage of the very intuitive query forms, the users can easily access, navigate, filter and select the putative gene fusions for further validations and studies. They can also find suitable experimental models for a given fusion of interest.<br>Conclusions: We believe that the LiGeA resource can represent not only the first compendium of both known and putative novel gene fusion events in the catalog of all of the human malignant cell lines, but it can also become a handy starting point for wet-lab biologists who wish to investigate novel cancer biomarkers and specific drug targets.
**Corresponding Author:**	Tiziana Castrignano', PhD<br>Cineca<br>Rome, Italy ITALY
**Corresponding Author Secondary Information:**	
**Corresponding Author's Institution:**	Cineca
**Corresponding Author's Secondary Institution:**	
**First Author:**	Silvia Gioiosa, PhD
**First Author Secondary Information:**	
**Order of Authors:**	Silvia Gioiosa, PhD
	Marco Bolis
	Tiziano Flati
	Annalisa Massini

Powered by Editorial Manager® and ProduXion Manager® from Aries Systems Corporation

	Enrico Garattini
	Giovanni Chillemi
	Maddalena Fratelli
	Tiziana Castrignano', PhD
**Order of Authors Secondary Information:**	
**Response to Reviewers:**	Response to Editor:<br>Q.1.: " the reviewers point out that the web tool is extremely slow - can this be improved? This is an important point from the user's perspective. Please note the performance metrics kindly provided by reviewer 1 (attached)."<br>A.1: We thank the editor and the reviewers for kindly providing this feedback about the web tool performance. We do also believe that this point is very crucial from user's perspective, therefore we greatly improved the speed of the web site by leveraging both the tools and metrics suggested by the reviewer as well as including new metrics, such as PageSpeed and gtmetrix. We included all the metrics results as attachments or direct hyperlinks. We would kindly invite you to clear the browser's cache before navigating the website.<br>Q.2: "reviewer 1 has some useful suggestions for additional data that should be linked, e.g. genomic coordinates. I feel this would go some way in also addressing the concerns of reviewer 2 that the tool did not present a sufficient advance in its present form."<br>A.2: Thanks to the suggestions of the reviewer, we have now included in our portal a great variety of links, both to external resources (e.g., Gene Cards, Cancerxgene, among others) as well as to internal brand-new pages, such as those dedicated to the description of cell lines (e.g., http://hpc-bioinformatics.cineca.it/fusion/cell_line/Detroit562/) and of fusion events (e.g., http://hpc-bioinformatics.cineca.it/fusion/fusion_event/fus_49998/). Not only do these additional web pages meet the requests from reviewer 1 but, at the same time, they also foster the integrability and the interlinking across other topic-related resources.<br>Q.3: "reviewer 2 points out that one of the tools, EricScript, finds many more fusion genes than the other two. Why? This needs to be thoroughly addressed and explained in the manuscript."<br>A3: We have described in great detail the filtering process of the outputs from EricScript both in the manuscript and in the response to reviewer 2. After the filtering process, the dataset size from ES results more balanced compared to the other two softwares. Currently, LiGeA reports a set of 293,244 ES predictions which is greatly reduced from the initial size.<br>Q.4: "Please add licence information about any new code to your manuscript, under the "Availability and Requirements" section: (see https://academic.oup.com/gigascience/pages/instructions_to_authors#Preparing Main Manuscript Text). We usually also host an archival copy ("snapshot") of new code in our GigaDB repository. In addition, please register any new software application / database in the SciCrunch.org database to receive a RRID (Research Resource Identification Initiative ID) number, and include this in your manuscript."<br>A.: As requested, we have added licence information an RRID number under the "Availability and Requirements" section.<br><br>Point by point response to reviewer 1:<br>Major issues:<br>Q.1: "I've tried the database using both Chrome and Safari browsers (on several high-end laptops) and found that it is extremely slow/laggy. I mean the overall interface responsiveness. E.g. Chrome audit metrics rate the performance as 18/100 (see attachment). The 'Gene pairs statistics' shows a loading screen for around 30 seconds and then fails showing generic Chrome crash tab. It seems that the situation improves a bit after browsing the web page for a while because of caching. The web portal performance should be definitely optimized. In my humble opinion (I'm not a professional web developer), it can be improved by switching from normal Angular bindings to one-time bindings for variables that will not be updated (https://docs.angularjs.org/guide/expression, One-time binding section)."<br>A.1: We have greatly improved the web portal performance. Now Chrome audit metrics rates the performance as 30/100 (instead of 18; inspect results by loading the files attached on https://googlechrome.github.io/lighthouse/viewer/). As an approximate

comparison, take into account, for example, that well-known sites (such as www.cnn.com) score a performance equal to 1 (inspect the corresponding attached file). Switching to Angular one-time bindings was not a feasible option for us, since the website is built on top of a powerful, general-purpose framework we have developed in-house which is able to display arbitrary content on-the-fly (i.e., it is not possible to predict which parts will change and which will not).
In order to improve the performance of the Fusion database, we profiled the website pages and focused on improving the speed of the search pages as well as of the overall content (e.g., Download page). In order to accomplish this, we have speeded up all the queries (i.e., reducing the server-side response times) by improving and simplifying the structure of the underlying database. Also, all dropdown menus which are associated with thousands of entries (e.g., those associated with cell lines, genes or transcripts) have been converted into autocomplete fields which show only the first n matching items, thus reducing loading time significantly.
We have also fixed the problems regarding the "Gene pair statistics" page and now the page loads instantly (http://hpc-bioinformatics.cineca.it/fusion/gene_statistics).
Also the Download page has been made much faster by means of pagination: in fact, we realized that most of the times the website looked laggy because too many items were displayed (e.g., 935 rows for the Download page).
Finally, now the portal loads in half of the time (5 seconds instead of 10, according to gtmetrix - see URLs at the bottom of the answer) and appears much more responsive when browsing through the pages (total page size around 1MB).
Also, if using PageSpeed as speed metrics, the quality of the new portal has increased from 43 to 65, demonstrating the neat improvement in terms of speed and response time (please, see attachments).
We believe that everything is now fixed and we invite the reviewer to clear the browser's cache before navigating the website in order to appreciate the added changes. Still, should some page require further curation, we will make sure to fix it and/or improve it.
Q.2:"LiGeA database can benefit from providing users with a table containing a generic LiGeA fusion id and fusion genomic coordinates. These ids should be linked to other tables containing additional information on fusions: 5' and 3' genes, cell line identifier, COSMIC ids, etc. The http://hpc-bioinformatics.cineca.it/fusion/downloads/ link can be fetched via wget, yet it contains lots of intermediate processing files and no README descriptions in subfolders. This will make the life easier for bioinformaticians by allowing them to download the plain-text database version and use it for downstream analysis and annotation of RNA-Seq results without spending significant time on parsing/assembling database files."
A.2: We thank reviewer 1 for the very useful suggestions. We have assigned a generic and linkable LiGeA fusion ID to any fusion event. By clicking on it, another web page opens and the user can view additional information e.g. involved cell line and human disease, supporting algorithm(s), involved genes and genomic coordinates among the others. For example, by clicking on this link (http://hpc-bioinformatics.cineca.it/fusion/fusion_event/fus_16084/), the user can have a look at specific information regarding the gene couple PML-RARA in NB4 cell line.
Moreover, we have integrated the "Dataset" section with dedicated web pages to each Cell line identifier (e.g. http://hpc-bioinformatics.cineca.it/fusion/cell_line/CCLE_019), resuming cell line specific details, such as linkable COSMIC id, original tissue, most affected chromosomes and integrated genomes from host viruses.
Finally, as suggested by reviewer 1, we have now organized the Download page by creating a whole tar.gz file containing all the final files, thus allowing the user to download the plain-text database version. As a plus, thanks to the pagination discussed above, we give the opportunity to download single files as well, without affecting the speed of this web page.

Minor issues:
Q.3:"The authors should compare the list of fusion events in LiGeA and previously described fusions from other datasets (those listed in Table#1, e.g. Mitelman database). Although there is some information in the Data Statistics and Validation section of the manuscript, an additional figure or table comparing LiGeA with existing databases should be added to the manuscript."
A.3: "There is an interactive panel on LiGeA portal, under the Database Statistics menu, named "Intersection of our database with existing databases". The interactive Venn Diagram shows how many pGFEs (predicted Gene Fusion Events) in the CCS

Powered by Editorial Manager® and ProduXion Manager® from Aries Systems Corporation

(Consensus call set) shown under LiGeA portal, are already present in other databases listed in Table#1 (e.g. chimerdb2, Cosmic, TCGA and so on). As suggested, we have added this figure to the manuscript (Fig. 1C).

Q.4: "The authors should comment on their specific choice of the fusion calling algorithms. Perhaps including additional fusion detection software such as STAR can yield more fusions/increase the confidence of existing fusion calls?"
The choice of the algorithms was driven by the paper from Kumar S. et al., (Nature, 2016), which compared twelve methods for the fusion transcripts detection from RNA-Seq data. In this paper, for each tool TOPSIS (Technique for Order of Preference by Similarity to Ideal Solution) scores were calculated by weighting four criteria i.e. sensitivity, time consumption (minutes), computational memory (RAM), and PPV (Positive Predictive Value). EricScript and FusionCatcher gained the best TOPSIS scores (0.93 and 0.87, respectively), followed by Bellerophontes and FusionMap (0.84 for both). Unfortunately, the latter two softwares presented a bug when trying to annotate gene fusions on hg38 human genome version. Therefore, we chose the Tophat-Fusion algorithm which was ranked immediately after (0.74). We have now added a more detailed explanation of the reasons leading to the specific choices of these tools, under the "Methods" section of the manuscript. As suggested by reviewer 1, we had a look at STAR but, to our knowledge, STAR is a read aligner and, moreover, FusionCatcher already relies on Bowtie, Blat, and STAR aligners to predict gene fusions. On the other hand, STAR-Fusion is a novel algorithm for fusion detection for which only a preprint abstract version of the paper is available at the moment (https://www.biorxiv.org/content/early/2017/03/24/120295). We would rather prefer to take advantage of peer-reviewed algorithms but we do not exclude to integrate results from other softwares in the future versions of the database.

Point by point response to reviewer2:
Q.1: "First, one of software is giving *too many* fusion events compared with the other two. It means either one of software is giving incorrect results. Whatever the results are, if they are giving more than 10 times bigger results than other software, it means it is not acceptable. There are a tons of software for this purpose - finding fusion genes. Authors need to be very careful when choosing some of them because detecting fusion events from RNA-Seq require very sophisticated optimization and filtering process as well as long calculation time. Authors need to do additional filtering steps for results from EricScript. Otherwise users will suspect something wrong with the final dataset."
A.1: We thank reviewer 2 for pointing out this very important issue.
It is true that tons of softwares exist for the purpose of finding fusion genes, but the choice of EricScript was driven by this very useful assessment (Kumar S. et al., Nature, 2015), which compared twelve methods for the fusion transcripts detection from RNA-Seq data indicating EricScript as the most performing one both in terms of sensibility and Positive Predictive Value.
Anyway, we agree with reviewer 2 that EricScript final results were too many compared to the other two. Therefore, as suggested, we did additional filtering steps for results from EricScript (initial dataset size: 929,638 predicted Gene Fusion Events - pGFEs).
First of all, we removed all the predictions for which the software was not able to predict an exact breakpoint position because such pGFEs could not even be experimentally validated (# of events passing the filter: 748,066).
Secondly, as already applied to FusionCatcher and Tophat Fusion results, we retained the pGFEs exhibiting at least 3 spanning reads over the gene fusion junction (# of events passing the filter: 486,174).
Furthermore, we filtered out all the pGFEs with Ericscore value less than 0.85. EricScore is a ranking parameter ranging from 0.5 to 1: greater values correspond to better predictions (# of events passing the filter: 293,244). Interestingly, by applying these filters, we filtered out almost 2/3 of the predictions from EricScript but, at the same time, the Consensus CallSet did not reduce substantially (from 3,294 to 2,926), thus indicating that the choice of a Consensus of predictions is a good strategy to obtain a reliable set of gene fusion candidates to be experimentally validated (please, see attached figure - also provided as supplementary material to the article). Currently, LiGeA reports a set of 293,244 ES predictions which is greatly reduced from the initial size. We are aware that this is still a high number as compared to the results of the other two algorithms and is probably bound to contain a higher number of false positives. However, the purpose of the present database is to provide scientists with a broad overview of the possible gene-fusion events in cancer cell lines. Depending on

its interests, each user will be able to decide whether to look for high-confidence, well established, already described fusions, or for low-confidence but potentially interesting and novel events. In the latter case, a further experimental validation will be obviously needed.

Q.2:"Second, p.4 line 18. Data Statistics and Validation section. Instead of overall statistics, 95% overlap with previously known cancer gene, please give how exactly it can detect experimentally validated fusion events from individual cell lines."
A.2: We thank reviewer 2 for this suggestion and we believe that this sentence was misunderstood and needed to be reformulated in the manuscript as follows: "As a validation of our analysis, 644 out of the 699 (92%) genes known to be functionally implicated in cancer and collected under COSMIC gene census (http://cancer.sanger.ac.uk/census), are present in our final dataset."
Furthermore, we give information about how exactly we can detect cell line-specific experimentally validated fusion events thanks to the colorful signature shown in LiGeA portal under the "Search for" tabs. Indeed, whenever a gene fusion couple has been already described as true positive whether in the literature or in other databases, a green circle is added to the gene fusion event. In this way, the user can choose events not tagged with the green circle and be addressed to further study the novel predictions only.

Q.3: "Finally, web-server is just showing calculation results from three software. If one browses that database, he/she can only get information which software is giving this results. But no novel intuitions or dataming from each content.
Thank you for our huge work, but readers need at least one scientific intuition or improvement from them. And, the database is very slow due to heavy use of javascript (I don't know exactly what WWW techniques are used). I think the database itself is not that big, and it could be improved. And, the database is very slow due to heavy use of javascript (I don't know exactly what WWW techniques are used). I think the database itself is not that big, and it could be improved."
A.3: Indeed, we feel that the LiGeA database Portal is not only a mere collection of calculation results. Its primary aim is indicating which putative fusion gene events could be experimentally validated and studied. About half of the Consensus Call Set is represented by fusion genes not yet described neither in the literature nor in other dedicated databases, therefore we believe that LiGeA could become a handy resource for many wet lab biologists who take advantage of cell lines in order to study human malignancies and oncogenic gene fusions. In addition, LiGeA is integrated with other useful external resources, thus allowing the extraction of further biologically meaningful information. For example:
Whenever the gene fusion couple has already been experimentally validated, an external link to COSMIC database (http://cancer.sanger.ac.uk/cosmic, a catalog of somatic mutations in cancer) is shown;
Whether one of the two genes involved in the fusion event has been already described to be causally implicated in cancer, an external link to the Cancer Gene Census is provided (http://cancer.sanger.ac.uk/census);
A colourful signature has been added to tag the FusionCatcher predictions as 'validated truly positive couple' (green circle), 'validated false positive couple' (red circle) and 'false positive couple with medium probability' (orange circles).
A functional prediction score (oncogenic potential, i.e. the probability of being 'driver' events in carcinogenesis) obtained by extensively running the Oncofuse software (http://www.unav.es/genetica/oncofuse.html), is reported as additional tag to each of the three kinds of results.
We agree that downstream analysis on this huge amount of data could hint further biological intuitions and it is for this reason that we have increased the data accessibility and fostered the easiness of data download by providing access also to the plain-text database version under the Download page and thus encouraging users to re-use our data.
Moreover, we would like to underline that the main focus of Giga Science is promoting reproducibility of analyses and big data dissemination, organization, understanding, and use. In particular, Data Notes "highlights and helps to contextualize exceptional datasets to encourage reuse… Data Notes focus on a particular dataset, and provide detailed methodology on data production, validation, and potential reuse. Supporting the FAIR Principles for scientific data management and stewardship that state that research data should be Findable, Accessible, Interoperable and Reusable."

	As regards the overall website speed, we have dramatically improved the loading and response time of the portal. Now the site loads very quickly (around 1 second) and, after improving the structure of the database and applying small fixes (e.g., pagination in the Download page, autocomplete fields in the search pages), browsing turned out to be much easier. We invite the reviewer to clear the browser's cache before navigating the website in order to appreciate the added changes.<br><br>Point by point response to reviewer 3:<br>Q.1:"On page 1, line 12, the "gene fusion events result from chromosomal rearrangements" should be changed to "oncogenic gene fusion events result from chromosomal rearrangements" because fusion genes occur also in healthy organisms. Fusion of genes is also one of the evolutionary mechanism for creating a new gene in a healthy organism. Not all fusion genes are oncogenic. For example, there are plenty of fusion genes known to exist in healthy people, like for example TTTY15-USP9Y, SLC45A3-ELK4, MSMB-NCOA4."<br>A.: We changed the sentence as suggested.<br><br>Q.2: "On page 1, lines 37-40, the text "Moreover, each gene fusion predictions differes...chromosomal rearrangements (Mertens et al.; 2015)" should be removed from the article because it is not correct. This is not correct because some fusion gene finder can call very well a certain type of fusion genes whilst all the other fusion caller will miss the and therefore the consensus here is not the best. For example, FusionCatcher is the only fusion finder which is able to call IGH fusions (see: https://doi.org/10.1182/blood-2016-12-758979)."<br>A.: We thank reviewer 3 for this suggestion and we agree that FusionCatcher is the only fusion finder able to call IGH fusions (named IGH@ by FusionCatcher annotation). Indeed, since we also believe that the consensus method is not necessarily the best choice, we give the opportunity to navigate the results from individual algorithms and by means of the dedicated "search by algorithm" function. Therefore we removed the sentence on page 1, lines 37-40 and we have added the reference cited by the reviewer to the article (https://doi.org/10.1182/blood-2016-12-758979).<br>Nevertheless, many other known gene fusion couples reported in literature (e.g. FGFR3-TACC3, PML-RARA, BCR-ABL1 just to cite some) have been correctly identified by all the softwares we used. Since the aim of LiGeA portal is addressing researchers to study and validate potential novel cell line-specific gene fusion events, we believe that researchers could benefit as well from choosing fusion candidates predicted from more than one algorithm. Choosing more "reliable" targets might help them in saving time and resources, speeding up the process of experimental validation and this is why we have built the Consensus Call Set. Anyway, we don't want to claim that one method is better than the other, since we believe that it is up to the wet lab biologists to choose the way they prefer to select the targets to validate and to study.<br><br>Q.3: "When searching for a gene using the http://hpc-bioinformatics.cineca.it/fusion/search_for_gene is very slow. This should be fixed. Also it is very important to list and show the fusion genes which are supported only by one fusion finder. For example the fusion DUX4-IGH is known to exist in NALM6 cell line which is one of the 935 cell lines but when looking for it in LiGeA database, it does not show up because the TOPHAT-fusion and EricScript are not able to find fusions which involve DUX4 gene or IGH gene."<br>A.3: We thank reviewer 3 for suggesting to improve the "search for gene" function and now it is definitely faster. We invite the reviewer to clear the browser's cache before navigating the website in order to appreciate the added changes.<br>Furthermore, we would like to point out that it is already possible to list and show the fusion genes supported only by one fusion finder. Whenever the user submits a query, it is possible to select the outputs from one, two or three algorithms by checking the "show results supported by at least n algorithms" menu. Even if the user does not use this facility, the results are simply paginated and sorted by those supported by more algorithms followed by those supported only by one.<br>Eventually, it is also possible to query the Database by the "Search by algorithm" function in order to choose one's own algorithm of election.
**Additional Information:**	

Powered by Editorial Manager® and ProduXion Manager® from Aries Systems Corporation

Question	Response
Are you submitting this manuscript to a special series or article collection?	No
**Experimental design and statistics**<br><br>Full details of the experimental design and statistical methods used should be given in the Methods section, as detailed in our Minimum Standards Reporting Checklist. Information essential to interpreting the data presented should be made available in the figure legends.<br><br>Have you included all the information requested in your manuscript?	Yes
**Resources**<br><br>A description of all resources used, including antibodies, cell lines, animals and software tools, with enough information to allow them to be uniquely identified, should be included in the Methods section. Authors are strongly encouraged to cite Research Resource Identifiers (RRIDs) for antibodies, model organisms and tools, where possible.<br><br>Have you included the information requested as detailed in our Minimum Standards Reporting Checklist?	Yes
**Availability of data and materials**<br><br>All datasets and code on which the conclusions of the paper rely must be either included in your submission or deposited in publicly available repositories (where available and ethically appropriate), referencing such data using a unique identifier in the references and in the "Availability of Data and Materials" section of your manuscript.<br><br>Have you have met the above requirement as detailed in our Minimum Standards Reporting Checklist?	Yes

Powered by Editorial Manager® and ProduXion Manager® from Aries Systems Corporation

Manuscript Click here to download Manuscript revision-LiGeA.pdf

OXFORD (GIGA)^n SCIENCE

GigaScience, 2017, 1–8

doi: xx.xxxx/xxxx
Manuscript in Preparation
Data Note

DATA NOTE

Massive NGS Data Analysis Reveals Hundreds Of Potential Novel Gene Fusions in Human Cell Lines

Silvia Gioiosa[1,4,†], Marco Bolis[2,†], Tiziano Flati[1,4], Annalisa Massini[3], Enrico Garattini[2], Giovanni Chillemi[1], Maddalena Fratelli[2,*] and Tiziana Castrignanò[1,*]

[1]SCAI-Super Computing Applications and Innovation Department, CINECA, Rome, Italy, and [2]Laboratory of Molecular Biology, IRCCS-Istituto di Ricerche Farmacologiche "Mario Negri," Milano, Italy, and [3]Computer Science Department, Sapienza University of Rome, Italy, and [4]National Council of Research, CNR, Institute of Biomembranes, Bioenergetics and Molecular Biotechnologies, Bari, Italy.

[†]Contributed equally.
[*] To whom correspondence should be addressed: t.castrignano@cineca.it; maddalena@marionegri.it

Abstract

Background: Gene fusions derive from chromosomal rearrangements and the resulting chimeric transcripts are often endowed with oncogenic potential. Furthermore, they serve as diagnostic tools for the clinical classification of cancer subgroups with different prognosis and, in some cases, they can provide specific drug targets. So far, many efforts have been carried out to study gene fusion events occurring in tumor samples. In recent years, the availability of a comprehensive Next Generation Sequencing dataset for all the existing human tumor cell lines has provided the opportunity to further investigate these data in order to identify novel and still uncharacterized gene fusion events.

Results: In our work, we have extensively reanalyzed 935 paired-end RNA-seq experiments downloaded from "The Cancer Cell Line Encyclopedia" repository, aiming at addressing novel putative cell-line specific gene fusion events. The bioinformatics analysis has been performed by the execution of three different gene fusion detection algorithms. The results have been further prioritized by running a bayesian classifier which makes an *in silico* validation. The collection of fusion events supported by all of the predictive softwares results in a robust set of ~ 2,000 *in-silico* predicted novel candidates suitable for downstream analyses. Given the huge amount of data produced, computational results have been collected in a database named LiGeA. The database can be browsed through a dynamical and interactive web portal, further integrated with validated data from other well known repositories. Taking advantage of the very intuitive query forms, the users can easily access, navigate, filter and select the putative gene fusions for further validations and studies. They can also find suitable experimental models for a given fusion of interest.

Conclusions: We believe that the LiGeA resource can represent not only the first compendium of both known and putative novel gene fusion events in the catalog of all of the human malignant cell lines, but it can also become a handy starting point for wet-lab biologists who wish to investigate novel cancer biomarkers and specific drug targets.

Key words: Database; Human gene fusions; Cell Lines; NGS; Gene Fusion detection algorithms; Chromosomal rearrangements; Bioinformatics

Compiled on: January 9, 2018.
Draft manuscript prepared by the author.

Key Points

- A massive bioinformatics analysis conducted on Paired-End RNA-seq samples from 935 human malignant Cell Lines reveals a landscape of known and novel *in-silico* predicted gene fusion events;
- LiGeA Portal represents a user-friendly database for the visualization and interrogation of the results;
- LiGeA Portal is further integrated with information from other databases and with gene-fusion priotirization analysis, in order to address targeted experimental validations on a highly reliable set of candidate gene fusions.

Background

Oncogenic gene fusion events result from chromosomal rearrangements which lead to the juxtaposition of two previously separated genes. The accidental joining of DNA of two genes can generate hybrid proteins. It can also result in the misregulation of the transcription of one gene by the *cis-regulatory* elements (promoters or enhancers) of another, sometimes resulting in the production of oncoproteins that bring the cell to a neoplastic transformation (Mitelman et al.; 2007). Not only gene fusions can have a strong oncogenic potential (Mertens et al.; 2015), but they also serve as diagnostic tools for the clinical classification of cancer subgroups with different prognosis and, in some cases, they may provide specific drug targets (Seratì et al.; 2016). For instance, the presence of the PLM-RARA fusion product is a specific hallmark of acute promyelocytic leukemia (APL) (Borrow et al.; 1990) and represents the first example of gene-fusion targeted therapy (Nervi et al.; 1998) that has changed the natural history of this disease. Hence, there are several reasons why studying gene fusions in cancer is very important. In recent years, Next-Generation Sequencing (NGS) technologies have played an essential role in the understanding of the altered genetic pathways involved in human cancers. Nowadays, most of the studies aiming at fusion discovery use NGS techniques followed by massive bioinformatics analyses. The greatest challenge of these sophisticated algorithms of prediction is the ability to discriminate between artifacts and really occurring chromosomal rearrangements (Lou et al.; 2009). Moreover, each gene fusion predicting software differs in terms of sensitivity and specificity. In the last decade, much effort has been done to catalog gene fusion events, thus resulting in a wide production of databases. At present, a dozen of published databases regarding oncogenic fusion genes exists (see table 1 for a summary). Some of them (e.g. FusionCancer, ChiTaRS-3.1) collect *in silico* predictions of chimeric genes, obtained analyzing publicly available datasets derived from heterogeneous sources either in terms of experimental material (a mix of Single-End and Paired-End RNA-seq data, ESTs) and in terms of data source (patients and cell lines). Some others collect gene fusion events with experimental evidences manually curated from literature collection (e.g. TCGA, Mitelman, TICdb, COSMIC, ONGene). In this work we focused on the whole catalog of Human malignant Cell Lines, thus obtaining a homogeneous input NGS dataset covering several human malignancies. We exerted an extensive bioinformatics analysis of 935 paired-end RNA-seq samples derived from 22 different tumor tissues and used a combination of the best performing gene fusion-detecting algorithms. For ease of understanding, we define the predicted Gene Fusion Event (pGFE) as the entity constituted by the gene fusion couple in a specific cell line and designate the Consensus Call-Set (CCS) as the number of pGFEs supported by all the used algorithms. Starting from this assumption, we obtained a total of 339,899 pGFEs, 2,926 of which belonging to the CCS. Moreover, since not all the pGFEs can give rise to oncogenic transformations, the use of a priotirization software is recommended in order to distinguish between real driver mutations from passenger ones. Therefore, a robust Bayesian classifier has been used to perform an *in silico* validation of the results. Since one of the main purposes of our extensive big data analysis is encouraging the reuse of our results in order to experimentally validate the *in-silico* predictions, we set up a web portal collecting these data, LiGeA (cancer cell LInes Gene fusion portAl). It is possible to browse, search and freely download all the results obtained and described within this article at the LiGeA repository web page available at `http://hpc-bioinformatics.cineca.it/fusion/`. To our knowledge, our resource represents the first compendium of both known and predicted novel gene fusion events in cell lines from 22 different human tumor types.

Data Description

Methods

We have analyzed 935 paired-end RNA-seq experiments available at the Cancer Cell Line Encyclopedia repository, for a total of 32 TB of input raw data. The analysis has been carried out by using three different somatic fusion gene detection algorithms: FusionCatcher (Nicorici et al.; 2014), EricScript (Benelli et al.; 2012) and Tophat-Fusion (Daehwan and Salzberg; 2011). The choice of the algorithms was driven by the assessment from Kumar S. et al. (Kumar et al.; 2016), which compared twelve methods for the fusion transcripts detection from RNA-Seq data weighting four criteria (i.e. sensitivity, time consumption, RAM, and Positive Predictive Value) and assigning a score from 0 to 1. EricScript and FusionCatcher gained the best scores (0.93 and 0.87, respectively), followed by Bellerophontes (Abate et al.; 2012) and FusionMap (Ge et al.; 2011) (0.84 for both). Unfortunately, the latter two softwares presented a problem when trying to annotate gene fusions on hg38 human genome version or due to software errors occurring in the handling of intermediate files. Therefore, we chose the Tophat-Fusion algorithm which was ranked immediately after (0.74). Furthermore, the chosen softwares contain several layers of information in their output files, thus giving us the opportunity to collect and interconnect a wide set of additional data for each pGFE. Here is a short description of each fusion detection tool, accompanied by the versions and the used parameters.

- **FusionCatcher (FC)**: FC is a Python based algorithm. It executes a first mapping run with Bowtie v.1.2.0 (Langmead et al.; 2009) and then performs the Gene fusion detection basing on three different aligners: Bowtie2 v.2.2.9 (Langmead and Salzberg; 2012), BLAT v.36 (Kent; 2002) and STAR v.2.5.2b (Dobin et al.; 2013). FC takes advantage of NCBI Viral Genomes (v. 2016-01-06) in order to detect exogenous virus material integration into the host genome. Moreover, the FC algorithm compares its own output with a set of published databases, thus proving a detailed list of truly positive and false positive pGFE candidates. In our analysis we

Table 1. State of the art of databases reporting gene fusions

Database Name	URL	Short Description
Tumor Fusion Gene Data Portal	http://54.84.12.177/PanCanFusV2//	A collection of fusion genes in the Tumor Cancer Genome Atlas (TCGA) samples.
TICdb (Novo et al.; 2007)	http://www.unav.es/genetica/TICdb/	A collection of 1,374 fusion sequences extracted either from public databases or from published papers (last update: 2013).
chimerDB3.0 (Lee et al.; 2017)	http://203.255.191.229:8080/chimerdbv31/mindex.cdb	A catalog of fusion genes encompassing analysis of TCGA data and manual curations from literature.
ONGene (Liu et al.; 2017)	http://ongene.bioinfo-minzhao.org/	Literature-derived database of oncogenes
COSMIC Cell Lines	http://cancer.sanger.ac.uk/cell_lines	Gene fusions are manually curated from peer reviewed publications. Currently COSMIC includes information on fusions involved in solid tumors but not yet leukemias and lymphomas.
Mitelman (Mitelman et al.; 2007)	https://cgap.nci.nih.gov/Chromosomes/Mitelman	Reports hundreds of gene fusions associated with clinical reports but does not contain sequence data.
ChiTaRs-3.1 (Gorohovski et al.; 2017)	http://chitars.md.biu.ac.il/index.html	A collection of 34,922 chimeric transcripts identified by Expressed Sequence Tags (ESTs) and mRNAs from the GenBank, ChimerDB, dbCRID, TICdb and the Mitelman collection of cancer fusions for several organisms.
FusionCancer (Wang et al.; 2015)	http://donglab.ecnu.edu.cn/databases/FusionCancer/	591 samples, both single-end and paired-end RNA-seq, published on SRA (http://www.ncbi.nlm.nih.gov/sra) database between 2008 and 2014 covering 15 kinds of human cancers .

downloaded FC v. 0.99.5a and Ensembl genome annotation v.83 and used hg38/GRCh38 as genome assembly version. The software was executed with default parameters, requiring 111,620 CPU core hours, 125 GB of RAM and 20 CPUs to complete the execution on our input dataset. Overall, FC detected 26,669 pGFEs involving 9,160 genes.

- **Tophat-Fusion (TF)**: TF uses the Tophat-fusion-post function in order to create a filtered list of gene fusion candidates, starting from the output files obtained running Tophat with the "–fusion-search" option (Trapnell et al.; 2009)." The following commands were run subsequently:

```
tophat –o $Sample.output/ –p 20 –fusion-search –keep-fasta-order –bowtie1 –no-coverage-search –r 160 –mate-std-dev 34 –max-intron-length 100000 –fusion-min-dist 100000 –fusion-anchor-length 13 $BOWTIE_INDEX/hg38 $Sample_1.fastq $Sample_2.fastq

cd $Sample.output/

tophat-fusion-post -p 20 –skip-blast $BOWTIE_INDEX/hg38
```

Tophat-2.0.12 and samtools 0.1.19 versions were used for this study. This algorithm turned out to be the slowest of the three ones, taking about 200,000 CPU core hours, 20 CPUs and 125 GB of RAM in order to complete its runs on the whole input dataset. TF produces several output files but only the file named "results.txt", representing the filtered list of predicted gene fusions, was used for subsequent analysis. The results encompassing "Chromosome M" have been manually discarded from the final results, *in primis* because TF was the only one of the three algorithms reporting it, secondly because they represented *bona-fide* false positive outcomes. Overall, TF highlighted 34,199 pGFEs involving 11,035 genes.

- **EricScript (ES)**: ES is developed in R (R Development Core Team; 2008), perl and bash scripts. It uses the BWA aligner (Li and Durbin; 2009) to perform the mapping on the transcriptome reference and samtools v. 0.1.19 (Li et al.; 2009) to handle with SAM/BAM files. Recalibration of the exon-junction reference is performed by using BLAT (Kent; 2002). For the purposes of this project, BLAT v.36 was downloaded at http://genome-test.cse.ucsc.edu/~kent/exe/linux/. Moreover, it was necessary to download R v.3.3.1 and a bedtools version greater than 2.20 (here we used v. 2.24). For this study, ES version 0.5.5 was obtained at https://sourceforge.net/projects/ericscript/files/. The Ensembl Database v. 84 was downloaded from https://docs.google.com/uc?id=0B9s__vuJPvIiUGt1SnFMZFg4T1E&export=download and built locally using BWA software with the command:

```
bwa index -a bwtsw allseq.fa
```

A total amount of 130,900 CPU core hours, 125 GB of RAM and 20 CPUs was required to successfully complete the analysis. We further filtered out ES final results by removing all the predictions for which the software was not able to predict an exact breakpoint position because such pGFEs could not even be experimentally validated. Secondly, as also applied to FusionCatcher and Tophat Fusion results, we re-

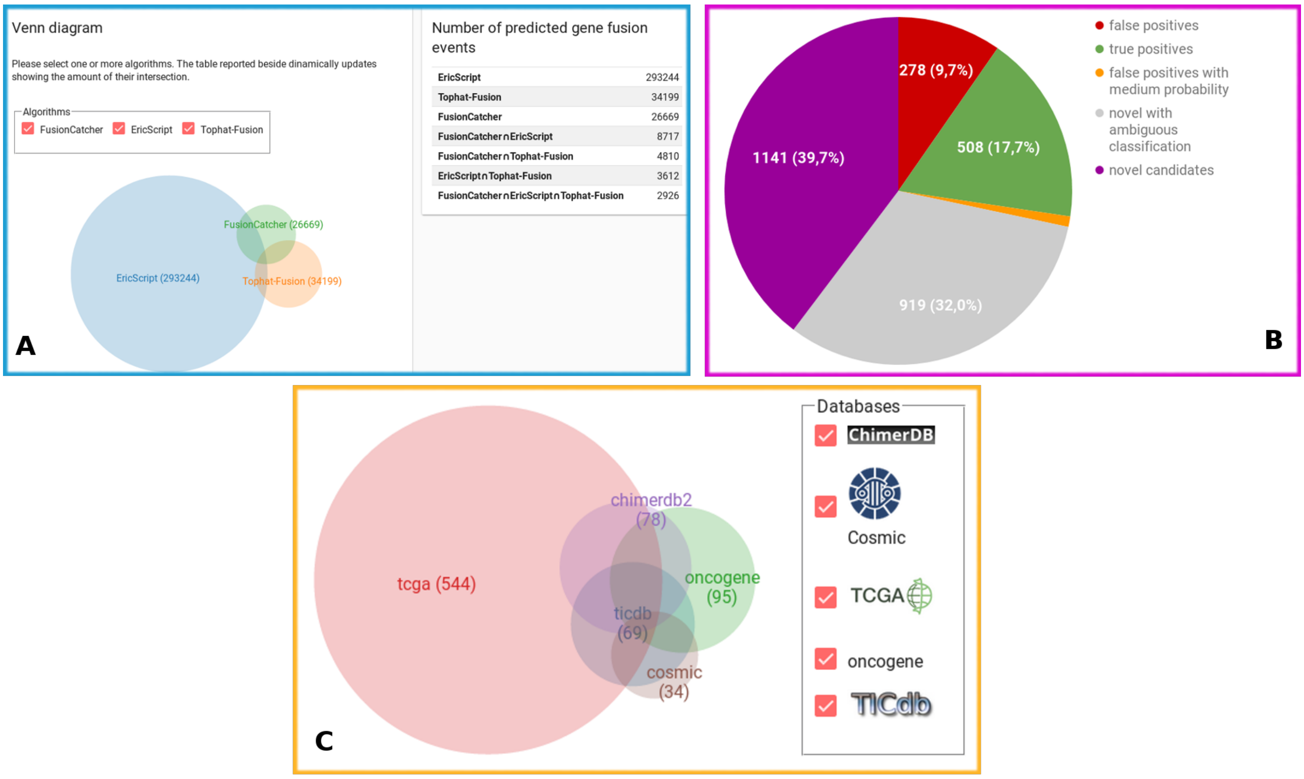

Figure 1. a) Venn diagram showing the intersection of the pGFEs identified by the three algorithms. b) Distribution of pGFEs in the Consensus Call-set: 39.7% (purple) of the CCS has not been previously described in any other database or scientific publication; 9.7% (red) and 17.7% (green) of the CCS have been reported in databases from healthy/tumoral samples thus representing the false/true positive subset of our analysis; 1% of the CCS (orange) reports tags which classify the pGFE as a false positive couple with medium probability; 32% (grey) of the results represent novel pGFEs tagged with values which classify them as both false and true positives. c) Venn diagram showing the intersection between the LiGeA CCS and other databases.

tained the pGFEs exhibiting at least 3 spanning reads over the gene fusion junction. Furthermore, we filtered out all the pGFEs with Ericscore value less than 0.85. EricScore is a ranking parameter ranging from 0.5 to 1: greater values correspond to better predictions. Interestingly, by applying these filters, we filtered out almost 2/3 of the initial predictions from EricScript but, at the same time, the CCS did not reduce substantially (from 3,294 to 2,926), thus indicating that the choice of a consensus of predictions is a good strategy to obtain a reliable set of gene fusion candidates to be experimentally validated (See supplementary Fig. 1). Overall, after the filtering process, ES detected 293,244 pGFEs involving 14,922 genes.

Data Statistics and Validation

Overall, our extensive analysis results in a CCS of 2,926 pGFEs and respectively 8,361/328,612 pGFEs supported by exactly two/one methods (Fig. 1A). As a first validation of our analysis, 644 out of the 699 (92%) genes known to be functionally implicated in cancer and collected under COSMIC gene census, are present in our final dataset. As a further validation of our results, about 1/4 of our CCS has already been published or is present in the following databases: chimerdb3; ONGene; COSMIC; tcga; ticdb (Fig. 1C). Finally, only a small subset of the pGFEs (~10% of data) present in the CCS have been recognized as false positive predictions, thus supporting the idea that a combination of algorithms can be of great utility in order to increase the sensitivity and the specificity of the tests. It is worth mentioning that, not only our analysis confirmed a large number of known gene fusion events, but it also highlighted 2,060 novel putative pGFEs in the CCS which could undergo further downstream analysis (Fig. 1B). Therefore, a further step of analysis was run with Oncofuse v.1.1.1 (Shugay et al.; 2013) in order to distinguish driver mutations (genomic abnormalities responsible for cancer) from passenger mutations (inert somatic mutations not implicated in carcinogenesis). Oncofuse is considered an *in silico* validation post-processing step which prioritizes the results obtained from each of the three algorithms. It assigns a functional prediction score to each putative fusion sequence breakpoint identified by the three softwares thus hinting which pGFEs are worthy of being experimentally validated and studied. Oncofuse supports multiple input formats such as the output from TF and FC. In order to run it also on the outputs from ES, a short pre-processing step was executed on these data. As suggested on Oncofuse manual, the accepted default input format is a tab-delimited file with lines containing 5' and 3' breakpoint positions. Therefore, these columns were extracted from ES output files and redirected into Oncofuse accepted input file. Oncofuse was run with default parameters using hg38 as the reference genome.

Availability of supporting data and materials

The datasets obtained and described within this article are freely searchable and downloadable at the LiGeA repository available at `http://hpc-bioinformatics.cineca.it/fusion/downloads`.

Database Description

LiGeA is a database server based on graph-db technology (Neo4j). The portal stores all of the results obtained from

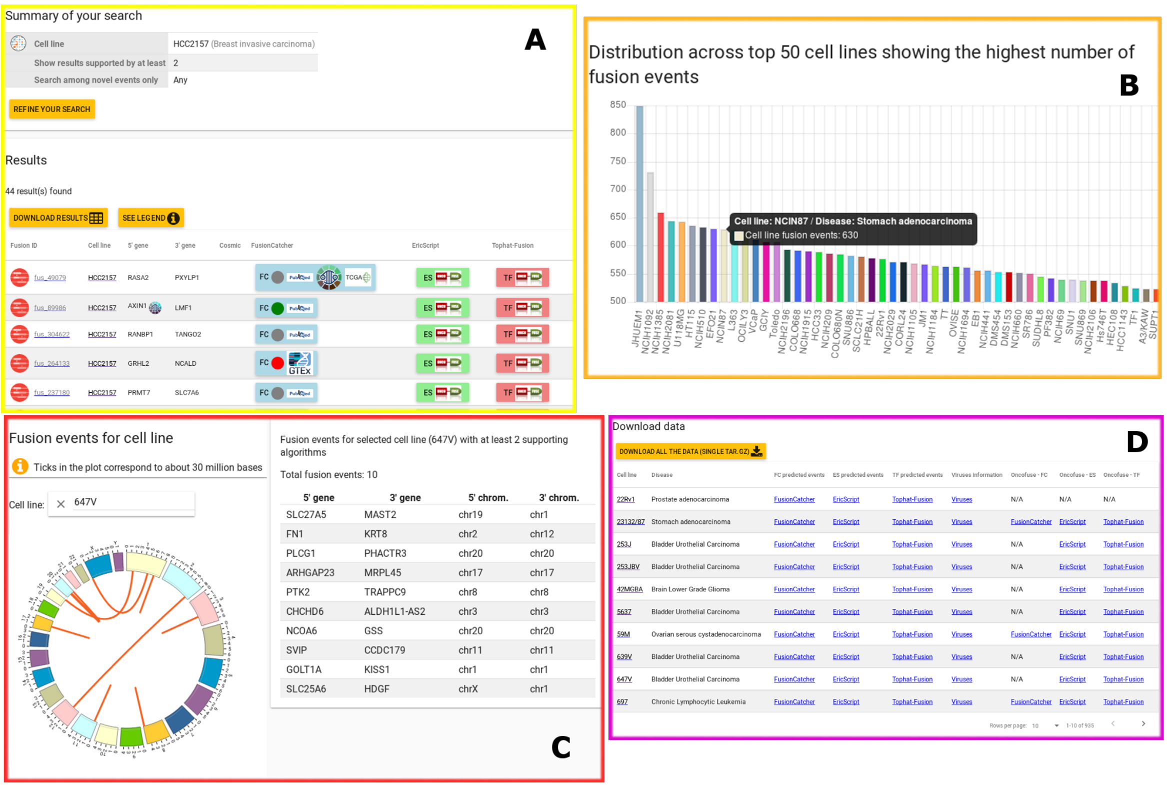

Figure 2. An overview of LiGeA portal. a) A 'Search by Cell line' example and the corresponding output; b) An overview of the input dataset; c) A circos diagram showing the graphical outcome of a 'Query by cell line' and the corresponding related table; d) An extract from the 'Download' web page.

each fusion gene predicting algorithm and the prioritization analysis outcome. Anyway, this database contains not only a mere collection of *in silico* predictions. Indeed, it has been integrated with other useful external resources in order to offer a carefully-curated web compendium. Here is a short list of the added features:

- Whenever the gene fusion couple has already been experimentally validated and published, an extra column with COSMIC icon is added to the results. By clicking on it, the user will be redirected to an external link containing a manually-curated catalog of 212 literature-derived somatic mutations in cancer (COSMIC; 2017a);
- Cancer Gene Census is a manually curated catalog of 699 genes for which mutations have been causally implicated in oncogenesis (Futreal et al.; 2004). Whenever one of the two genes involved in the pGFE has been already described to be implicated in cancer, the gene is tagged with an icon. By clicking on it, an external link to the Cancer Gene Census is provided showing a complete gene view (COSMIC; 2017b).
- A legend based on a colorful signature has been added to tag the FC predictions as 'validated truly positive couples' (green circle), 'validated false positive couples' (red circle), 'false positive couples with medium probability' (orange circle) and 'ambiguous signature' because tagged with both positive and negative values (grey circle) ;
- A functional prediction score obtained by extensively running the Oncofuse software, is reported as additional tag to each of the three algorithm outputs.

LiGeA portal is divided into several sections which allow a user-friendly navigation.

- **Home:** In the homepage, the user is provided with a quick overview of the database. A global summary table reports a numeric recapitulation (e.g. the number of genes/transcripts/exons collected into the portal; the number of predicted proteins and so on). Moreover, a histogram shows an abstract of the top 50 involved cell lines. By moving the cursor on the bars, a pop-up opens showing the cell line name and the corresponding number of the unique fusion events predicted by all the algorithms. Information about the algorithm predictions hosted into the portal are supplied with an interactive Venn Diagram linked to a dynamical table. Upon user selection of the algorithm/s of interest, both the diagram and the table refresh thus showing the resulting number of intersections.

- **Search:** This utility allows several searching options to browse and mine genomic-fusion events stored in LiGeA portal (see table 2 for an overview). All the resulting outputs are sorted by the number of algorithms supporting the fusion events, thus showing on the top of the table the most robust set of results. As additional feature, when specifying the features of interest, it is also possible to choose the minimum number of predicting algorithms. Search results are presented in the form of a paginated table containing those fusion events which satisfy the query parameters and data can also be downloaded in tabular format. Furthermore, by clicking on a given fusion ID, it is possible to access the event-specific page in which relevant information is presented in greater detail (e.g., involved cell line, disease, genes as well as links to external databases and resources). Two out of nine of the query forms ('search by fusion information' and 'search by virus') are specific annotations derived FC algorithm. Here is a short description of the provided searching utilities.
- 'Search by Disease': In this section, all the cell lines derived from the same disease have been grouped together. In this way, it is possible to navigate the gene fusions putatively

causing specific malignancies. The number of the cell lines constituting the queried subset is shown besides the pathology name.

- ’Search by Cell Line’: This module allows to navigate the database by indicating a specific cell line name. It is possible to tune the results by showing only the novel predictions not yet described in any other database or publication (Fig.2A).
- ’Search by Chromosome’: This query can be performed by inserting one or two chromosomes involved in the fusion event. The cell line name can be either indicated or not.
- ’Search by Gene’: the user can select up to two gene names (Gene Symbol or ENSEMBL ID) and the ’cell line’ form can be either selected or not. The genes reported in the query form are black if they are involved in pGFE and gray if they are not.
- ’Search by Transcript’: Since the same gene can give rise to different transcripts, it could be reasonable to query which of the transcripts produced by a specific gene are affected by a fusion event. This kind of query can be satisfied by inserting the Ensembl Transcript (ENST) IDs in the specific form.
- ’Search by Exon’: Some of the queries allow to go much more into molecular detail. This search can be done by inserting one or two exon IDs involved in the fusion event. The cell line name can be either indicated or not. In this way it is possible to highlight the specific exons which turn out to be fused in the final result.
- ’Search by Fusion information’: The pGFEs may have different predicted effects. Indeed, depending on the location of the chromosomal break points, the resulting protein may be in-frame, out-of frame, truncated and so on. Since the selectable values present in the fusion information form are specific of FC algorithm, the result of this query returns a table without ES and TF data. We suggest to view this section of FC manual https://github.com/ndaniel/fusioncatcher/blob/master/doc/manual.md#62---output-data-output-data in order to obtain a full description of all of the tags.
- ’Search by Algorithm’: this type of query is suitable for users who wish to navigate the outputs from specific softwares, choosing them individually or in combination. Indeed, it is known that some kind of fusions, such as those involving immunoglobulins, can be detected by specific softwares (Reshmi et al.; 2017).
- ’Search by Viruses’: Another useful information retrievable from the database regards virus sequence integration into the host genome. This search utility is virus-centered since it is possible to indicate or not the host cell line name. It is possible to select the virus name of interest (whether using GI ID or NC ID). Furthermore, a clickable link redirecting to the virus genome is also shown on the right of the table.

- **Statistics:** this section allows a visual inspection of the results. The four sub-menus are organized as follows:

  - ’Cell Line Statistics’: by choosing the Cell Line of interest, the resulting circular diagram shows all the chromosome couples involved in GFE predicted by at least two algorithms. The table on the right summarizes the resulting couples of the genes and chromosomes (Fig. 2C).
  - ’Chromosome Statistics’: this page reports a dynamical pie-chart showing the number of fusion events per human chromosome; by clicking on each slice of the pie, the related table automatically updates showing a chromosome summary statistics. Furthermore, information about the number of inter- and intra-chromosomal rearrangements detected by each algorithm is also reported.
  - ’Disease Statistics’: The ’Fusion Statistics’ pie-chart was produced by grouping together the cell lines derived from the same human pathology thus showing the total number of fusion events normalized by the number of cell lines composing a specific disease. The ’Virus statistics panel’ shows the frequency of exogenous virus integration per human malignancy.
  - ’Gene Statistics’: A word cloud diagram showing the most recurring pGFEs supported by three methods.
  - ’Database Statistics’: This sub-section is composed by four panels, the first regarding data in the CCS (Fig. 1B), the others relating only to FC results. In this page it is possible to get information about the number of pGFEs found in known databases (visualized as interactive Venn diagrams and tabular fashion) and the distribution of predicted effects (histogram view).

- **Dataset:** This page is a description of the input dataset used for the analysis. Among the above 1000 samples available at Broad institute portal CCLE repository, we downloaded 935 PE RNA-seq in fastq format. The SE samples have been discarded since the used softwares required it. The histogram in this section shows the number of the different cell lines derived from the same diseases (Fig. 2B). Furthermore, starting from this section, it is possible to access to web pages resuming cell-line specific details (e.g. COSMIC ID, drug resistance, human disease among others) .

- **Downloads:** From this panel it is possible to download all the processed data described within this article (Fig. 2D). Some of the files (’Summary information’ and ’Viruses information’) are specific products of FusionCatcher algorithm.

Availability and Requirements

- **Project name**: LiGeA: a comprehensive database of human gene fusion events
- **RRID**: SCR_015940
- **Project home page**: http://hpc-bioinformatics.cineca.it/fusion (GitHub project: https://github.com/tflati/fusion)
- **Operating system(s)**: Any
- **Programming language**: Python, JavaScript+HTML+CSS
- **Other requirements**: Django 1.10.5, Python 2.7.12, AngularJS 1.5.11
- **License**: GNU GPLv3

Declarations

List of abbreviations

LiGeA: cancer cell LInes GEne-fusions portAl; pGFE: predicted Gene Fusion Event; NGS: Next Generation Sequencing; TCGA: Tumor Cancer Genome Atlas; SRA : Sequence Read Archive; APL: acute promielocytic leukemia; CCS: Consensus Call-Set; FC: FusionCatcher; ES: EricScript; TF: Tophat-Fusion.

Consent for publication

‘Not applicable’

Competing Interests

The authors declare that they have no competing interests.

Table 2. Example of possible queries on LiGeA portal

Search by	Question	Query
Disease	’what are the fusion events present in stomach adenocarcinoma cell lines?’	Select ’stomach adenocarcinoma’ under ’disease’ menu
Cell Line	’what are the novel putative pGFEs affecting RH30(Sarcoma) cell line?’	Select ’RH30’ under the cell line menu and check the box ’show only novel results’
Chromosome	’what are the most suitable fusion partners for chromosome 8?’	Select ’Chr8’ either under the ’5’ Cromosome’ or under the ’3’ Chromosome’ tab and leave blank the other forms
Gene	’how many human cell lines show the PML-RARA fusion event?’	Select ’PML’ under the ’5’ gene menu’; Select ’RARA’ from the ’3’ gene menu’; leave blank the ’Cell Line’ query form;
Fusion information	’what are all the in-frame pGFEs in Jurkat cell line?’	select ’Jurkat’ under ’Cell line’ menu; Select ’in-frame’ under ’predicted effect menu
Fusion information	’what are the known GFEs predicted to be in-frame in Jurkat cell line?’	Select ’Jurkat’ under ’Cell line’ menu; Select ’in-frame’ under ’predicted effect menu; select ’known’ under ’Fusion description’ menu
Algorithm	’show only those GFEs supported by FC and TF in RH30 cell line’	Select ’RH30’ under ’Cell Line’ query form and check the boxes relative to FC and TF
Viruses	’which cell lines are most affected by Hepatitis C virus genome integration?’	Select ’Hepatitis C virus’ under ’Virus’ query form and let blank the ’Cell line’ query form

Funding

This work was supported by ELIXIR-IIB, CINECA and Regione Lombardia.

Author’s Contributions

TC and MF conceived and designed the work. All authors analyzed, interpreted data, wrote the manuscript and approved the final manuscript.

Acknowledgements

We acknowledge Andrea Micco for his useful tests on the first prototype of the system. We acknowledge the CINECA and the Regione Lombardia award under the LISA initiative 2016-2018, for the availability of high performance computing resources and support.

References

Abate, F., Acquaviva, A., Paciello, G., Foti, C., Ficarra, E., Ferrarini, A., Delledonne, M., Iacobucci, I., Soverini, S., Martinelli, G. and Macii, E. (2012). Bellerophontes: an rna-seq data analysis framework for chimeric transcripts discovery based on accurate fusion model, *Bioinformatics* **28**(16): 2114–2121.
URL: *http://dx.doi.org/10.1093/bioinformatics/bts334*

Benelli, M., Pescucci, C., Marseglia, G., Severgnini, M., Torricelli, F. and Magi, A. (2012). Discovering chimeric transcripts in paired-end rna-seq data by using ericscript, *Bioinformatics* **28**(24): 3232–3239.
URL: *http://dx.doi.org/10.1093/bioinformatics/bts617*

Borrow, J., Goddard, A., Sheer, D. and Solomon, E. (1990). Molecular analysis of acute promyelocytic leukemia breakpoint cluster region on chromosome 17, *Science* **249**(4976): 1577–1580.
URL: *http://science.sciencemag.org/content/249/4976/1577*

COSMIC (2017a). Cosmic database-wellcome trust sanger institute @ONLINE.
URL: *http://cancer.sanger.ac.uk/cosmic*

COSMIC (2017b). Cosmic gene census - wellcome trust sanger institute @ONLINE.
URL: *http://cancer.sanger.ac.uk/census*

Daehwan, K. and Salzberg, S. (2011). Tophat-fusion: An algorithm for discovery of novel fusion transcripts, *Genome Biology* **12**(8).

Dobin, A., Davis, C. A., Schlesinger, F., Drenkow, J., Zaleski, C., Jha, S., Batut, P., Chaisson, M. and Gingeras, T. R. (2013). Star: ultrafast universal rna-seq aligner, *Bioinformatics* **29**(1): 15–21.
URL: *http://dx.doi.org/10.1093/bioinformatics/bts635*

Futreal, P. A., Coin, L., Marshall, M., Down, T., Hubbard, T., Wooster, R., Rahman, N. and Stratton, M. R. (2004). A census of human cancer genes., *Nature reviews Cancer* **4**(3): 177–183.

Ge, H., Liu, K., Juan, T., Fang, F., Newman, M. and Hoeck, W. (2011). Fusionmap: detecting fusion genes from next-generation sequencing data at base-pair resolution, *Bioinformatics* **27**(14): 1922–1928.
URL: *http://dx.doi.org/10.1093/bioinformatics/btr310*

Gorohovski, A., Tagore, S., Palande, V., Malka, A., Raviv-Shay, D. and Frenkel-Morgenstern, M. (2017). Chitars-3.1—the enhanced chimeric transcripts and rna-seq database matched with protein-protein interactions, *Nucleic Acids Re-*

search **45**(D1): D790–D795.
URL: *+ http://dx.doi.org/10.1093/nar/gkw1127*

Kent, W. (2002). Blat—the blast-like alignment tool., *Genome Research* **12**(4): 656–664.

Kumar, S., Vo, A. D., Qin, F. and Li, H. (2016). Comparative assessment of methods for the fusion transcripts detection from rna-seq data, *Nature Scientific Reports* **6**.

Langmead, B. and Salzberg, S. (2012). Fast gapped-read alignment with bowtie 2., *Nature methods* **9**(4): 357–359.

Langmead, B., Trapnell, C., Pop, M. and Salzberg, S. L. (2009). Ultrafast and memory-efficient alignment of short dna sequences to the human genome, *Genome Biology* **10**(3): R25.
URL: *https://doi.org/10.1186/gb-2009-10-3-r25*

Lee, M., Lee, K., Yu, N., Jang, I., Choi, I., Kim, P., Jang, Y. E., Kim, B., Kim, S., Lee, B., Kang, J. and Lee, S. (2017). Chimerdb 3.0: an enhanced database for fusion genes from cancer transcriptome and literature data mining, *Nucleic Acids Research* **45**(D1): D784–D789.
URL: *+ http://dx.doi.org/10.1093/nar/gkw1083*

Li, H. and Durbin, R. (2009). Fast and accurate short read alignment with burrows–wheeler transform, *Bioinformatics* **25**(14): 1754–1760.
URL: *+ http://dx.doi.org/10.1093/bioinformatics/btp324*

Li, H., Handsaker, B., Wysoker, A., Fennell, T., Ruan, J., Homer, N., Marth, G., Abecasis, G. and Durbin, R. (2009). The sequence alignment/map format and samtools, *Bioinformatics* **25**(16): 2078–2079.
URL: *http://dx.doi.org/10.1093/bioinformatics/btp352*

Liu, Y., Sun, J. and Zhao, M. (2017). Ongene: A literature-based database for human oncogenes, *Journal of Genetics and Genomics* **44**(2): 119 – 121.
URL: *http://www.sciencedirect.com/science/article/pii/S1673852716302053*

Lou, D. I., Hussmann, J. A., McBee, R. M., Acevedo, A., Andino, R., Press, W. H. and Sawyer, S. L. (2009). High-throughput dna sequencing errors are reduced by orders of magnitude using circle sequencing, *Proceedings of the National Academy of Sciences of the United States of America* **110**(49): 19872–19877.

Mertens, F., Johansson, B., Fioretos, T. and Mitelman, F. (2015). The emerging complexity of gene fusions in cancer, *Nat Rev Cancer* **15**(6).

Mitelman, F., Johansson, B. and Mertens, F. (2007). "the impact of translocations and gene fusions on cancer causation", *Nat Rav Cancer* **7**(4): 233 – 245.

Nervi, C., Ferrara, F. F., Fanelli, M., Rippo, M. R., Tomassini, B., Ferrucci, P. F., Ruthardt, M., Gelmetti, V., Gambacorti-Passerini, C., Diverio, D., Grignani, F., Pelicci, P. G. and Testi, R. (1998). Caspases mediate retinoic acid-induced degradation of the acute promyelocytic leukemia pml/rarα fusion protein, *Blood* **92**(7): 2244–2251.
URL: *http://www.bloodjournal.org/content/92/7/2244*

Nicorici, D., Satalan, M., Edgren, H., Kangaspeska, S., Murumagi, A., Kallioniemi, O., Virtanen, S. and Kilkku, O. (2014). Fusioncatcher - a tool for finding somatic fusion genes in paired-end rna-sequencing data, *bioRxiv* .
URL: *http://www.biorxiv.org/content/early/2014/11/19/011650*

Novo, F., de Mendíbil, I. and Vizmanos, J. (2007). Ticdb: a collection of gene-mapped translocation breakpoints in cancer., *BMC Genomics* **8**(33).

R Development Core Team (2008). *R: A Language and Environment for Statistical Computing*, R Foundation for Statistical Computing, Vienna, Austria. ISBN 3-900051-07-0.
URL: *http://www.R-project.org*

Reshmi, S. C., Harvey, R. C., Roberts, K. G., Stonerock, E., Smith, A., Jenkins, H., Chen, I.-M., Valentine, M., Liu, Y., Li, Y., Shao, Y., Easton, J., Payne-Turner, D., Gu, Z., Tran, T. H., Nguyen, J. V., Devidas, M., Dai, Y., Heerema, N. A., Carroll, A. J., Raetz, E. A., Borowitz, M. J., Wood, B. L., Angiolillo, A. L., Burke, M. J., Salzer, W. L., Zweidler-McKay, P. A., Rabin, K. R., Carroll, W. L., Zhang, J., Loh, M. L., Mullighan, C. G., Willman, C. L., Gastier-Foster, J. M. and Hunger, S. P. (2017). Targetable kinase gene fusions in high-risk b-all: a study from the children's oncology group, *Blood* **129**(25): 3352–3361.
URL: *http://www.bloodjournal.org/content/129/25/3352*

Serratì, S., De Summa, S., Pilato, B., Petriella, D., Lacalamita, R., Tommasi, S. and Pinto, R. (2016). Next-generation sequencing: advances and applications in cancer diagnosis., *OncoTargets and Therapy* **9**: 7355–7365.

Shugay, M., Ortiz de Mendíbil, I., Vizmanos, J. L. and Novo, F. J. (2013). Oncofuse: a computational framework for the prediction of the oncogenic potential of gene fusions, *Bioinformatics* **29**(20): 2539–2546.
URL: *+ http://dx.doi.org/10.1093/bioinformatics/btt445*

Trapnell, C., Pachter, L. and Salzberg, S. L. (2009). Tophat: discovering splice junctions with rna-seq, *Bioinformatics* **25**(9): 1105–1111.
URL: *+ http://dx.doi.org/10.1093/bioinformatics/btp120*

Wang, Y., Wu, N., Liu, J., Wu, Z. and Dong, D. (2015). Fusioncancer: A database of cancer fusion genes derived from rna-seq data, **10**: 131.

Figure1 Click here to download Figure final.figure1.png

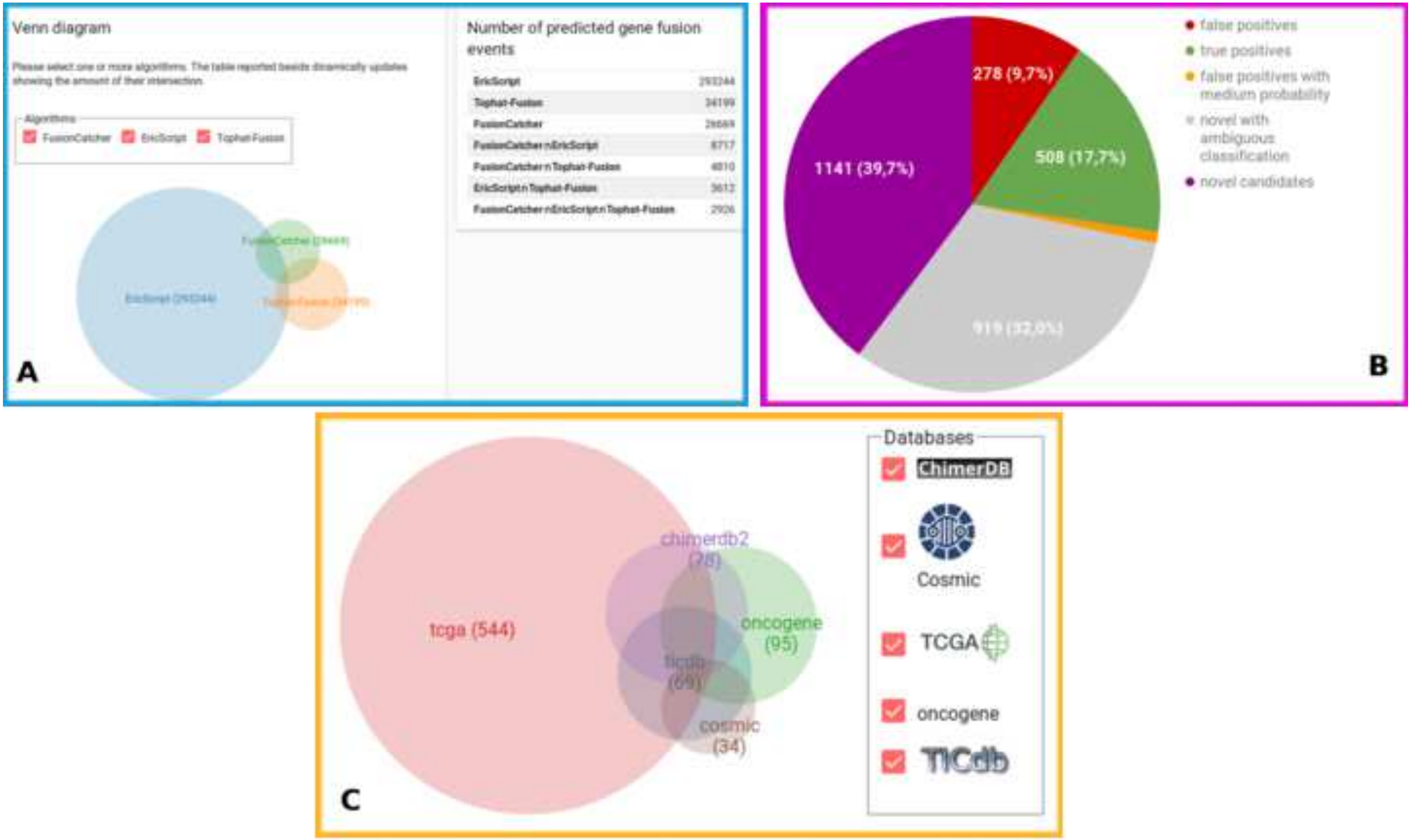

Figure2

Click here to download Figure final.Figura2.png

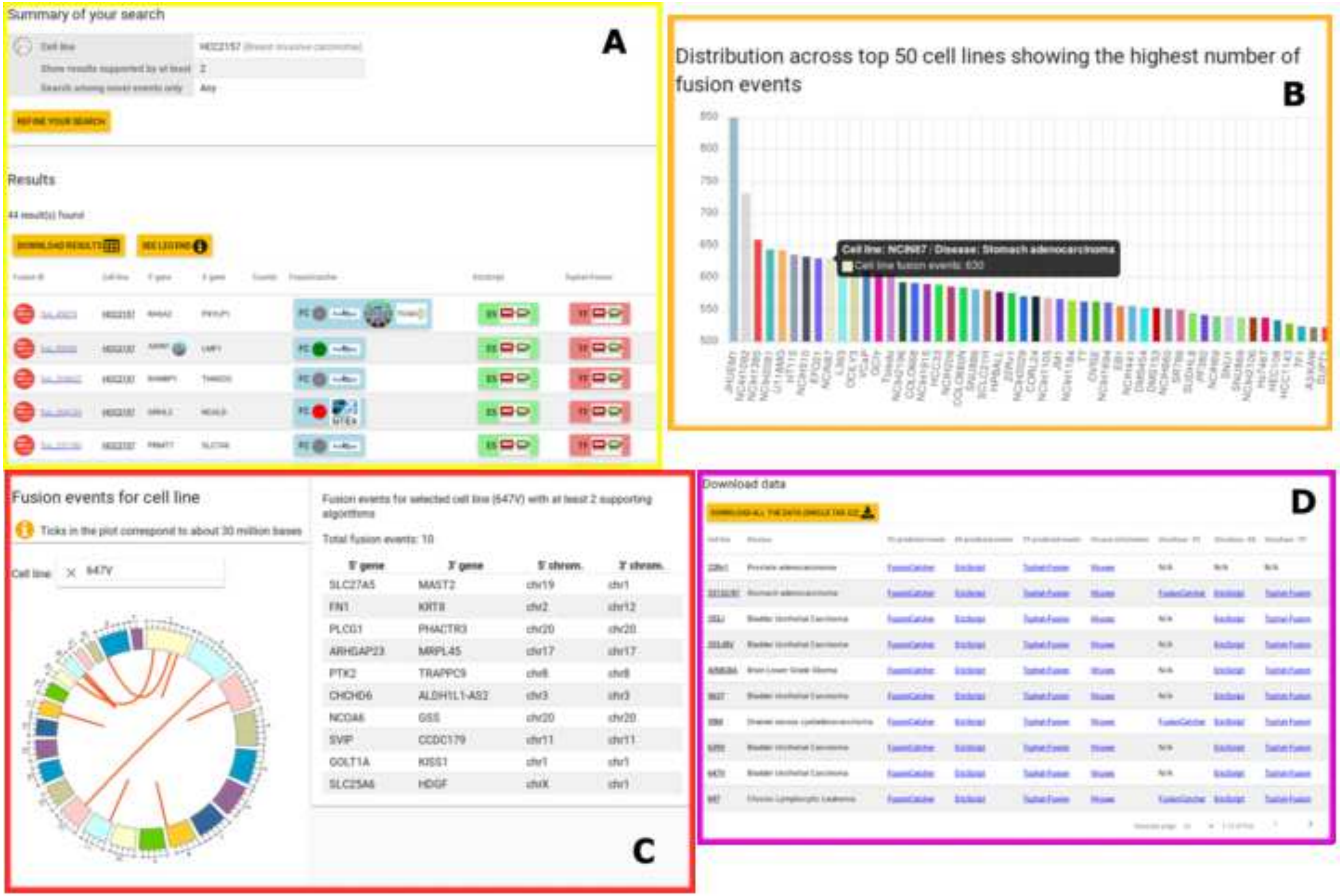

Supplementary figure 1 - This histogram shows the cumulative distribution on pGFEs identified by ES upon selecting different

Click here to access/download
Supplementary Material
Suppl.fig.1.png

Attached metrics for reviewers- GT metrix report before revision

Click here to access/download
Supplementary Material
GTmetrix-report- before.pdf

Attached metrics for reviewers- GT metrix report after revision

Click here to access/download
Supplementary Material
GTmetrix-report - after.pdf

Attached metrics for reviewers - page speed insights

Click here to access/download
Supplementary Material
PageSpeed Insights - before.pdf

Attached metrics for reviewers- page speed insights

Click here to access/download

Supplementary Material

PageSpeed Insights - after.pdf
