# Supplementary material for: Massive NGS data analysis reveals hundreds of potential novel gene fusions in human cell lines
Source: Gigascience. 2018 Jun 1;7(10):giy062. doi: 10.1093/gigascience/giy062 (PMC6207142; doi:10.1093/gigascience/giy062)

GigaScience

Massive NGS Data Analysis Reveals Hundreds Of Potential Novel Gene Fusions in Human Cell Lines

--Manuscript Draft--

**Manuscript Number:**	GIGA-D-17-00241R2
**Full Title:**	Massive NGS Data Analysis Reveals Hundreds Of Potential Novel Gene Fusions in Human Cell Lines
**Article Type:**	Data Note
**Funding Information:**	
**Abstract:**	Background: Gene fusions derive from chromosomal rearrangements and the resulting chimeric transcripts are often<br>endowed with oncogenic potential. Furthermore, they serve as diagnostic tools for the clinical classification of cancer<br>subgroups with different prognosis and, in some cases, they can provide specific drug targets. So far, many efforts have<br>been carried out to study gene fusion events occurring in tumor samples. In recent years, the availability of a<br>comprehensive Next Generation Sequencing dataset for all the existing human tumor cell lines has provided the<br>opportunity to further investigate these data in order to identify novel and still uncharacterized gene fusion events.<br>Results: In our work, we have extensively reanalyzed 935 paired-end RNA-seq experiments downloaded from "The Cancer<br>Cell Line Encyclopedia" repository, aiming at addressing novel putative cell-line specific gene fusion events in human<br>malignancies. The bioinformatics analysis has been performed by the execution of four different gene fusion detection<br>algorithms. The results have been further prioritized by running a bayesian classifier which makes an in silico validation.<br>The collection of fusion events supported by all of the predictive softwares results in a robust set of ~ 1,700 in-silico<br>predicted novel candidates suitable for downstream analyses. Given the huge amount of data and information produced,<br>computational results have been systematized in a database named LiGeA. The database can be browsed through a<br>dynamical and interactive web portal, further integrated with validated data from other well known repositories. Taking<br>advantage of the intuitive query forms, the users can easily access, navigate, filter and select the putative gene fusions for<br>further validations and studies. They can also find suitable experimental models for a given fusion of interest.<br>Conclusions: We believe that the LiGeA resource can represent not only the first compendium of both known and putative<br>novel gene fusion events in the catalog of all of the human malignant cell lines, but it can also become a handy starting<br>point for wet-lab biologists who wish to investigate novel cancer biomarkers and specific drug targets.
**Corresponding Author:**	Tiziana Castrignano', PhD<br>Cineca<br>Rome, Italy ITALY
**Corresponding Author Secondary Information:**	
**Corresponding Author's Institution:**	Cineca
**Corresponding Author's Secondary Institution:**	
**First Author:**	Silvia Gioiosa, PhD

Powered by Editorial Manager® and ProduXion Manager® from Aries Systems Corporation

**First Author Secondary Information:**	
**Order of Authors:**	Silvia Gioiosa, PhD
	Marco Bolis
	Tiziano Flati
	Annalisa Massini
	Enrico Garattini
	Giovanni Chillemi
	Maddalena Fratelli
	Tiziana Castrignano', PhD
**Order of Authors Secondary Information:**	
**Response to Reviewers:**	Dear Editor,<br>we have taken in great consideration the points raised during the process of revision and we have added a fourth algorithm and satisfied the requests from the reviewers.<br>We would kindly invite you and the reviewers to clear the browser's cache before navigating the website in order to appreciate the changes.<br><br>#####################Point by point answers to Reviewer#1:<br><br>RE A.1: I've checked new version of the web portal and confirm that the performance has improved substantially.<br>Answer RE A.1: Thank you.<br><br>RE A.2: Most recently published databases provide an option to download the full database as a single table. This is what was originally meant. Right now the authors provide the option to download raw results of Tophat, EricScript and FusionCatcher software, each in its own format, as a single gzipped archive with subfolders. While its helpful, it is not directly related to the database (otherwise it means that the database is just a collection of computational results without any systematization). It is also not that convenient for bioinformaticians, as stressed in my original question. So the authors should provide the users the ability to download the compiled database as a single plain-text table for the downstream analysis.<br>Answer RE A.2: The whole portal leverages on graph-based technologies (i.e., Neo4j, in our case) for resolving and executing all the queries raised by the portal's user. The database is not just a collection of computational results: the original raw data (given by the four algorithms along with a substantial amount of annotations and enrichment data) has been parsed, extracted and converted into a format suitable for graph-based data storage and representation. On the one hand, the most technical one probably, the systematization has consisted in the extraction of information and the subsequent formalization into a set of textual files representing the nodes and the relationships of the graph database, but on the other hand it implied parsing, filtering and analyzing the data in order to give it a sense and a value from a biological point of view (e.g., deciding to discard chromosome M or establishing interlinking with external resources, etc.).<br>In our previous release, the reason why we decided to make all the raw-data available for download was to allow the user to re-shape the same data into a different form, thus enabling a potential reuse of data (e.g., storage into SQL-like database, simple command-line post-processing or even further filtering and checking).<br>As regards the possibility to have a single plain-text file to import (as it was possible with SQL-like database), this is not possible anymore, to our knowledge. Despite this, Neo4j provides a tool for importing an existing database, starting from a set of plain-text CSV files. To this end, we now provide a tar.gz with i) a directory containing all the CSV files containing the systematized information and ii) a script for importing this data into Neo4j (this assumes you have Neo4j installed somewhere in your file system and only requires the Neo4j base directory).<br><br>RE A.4:<br>"> The choice of the algorithms was driven by the paper from Kumar S. et al., (Nature, 2016), which compared twelve methods for the fusion transcripts detection from RNA-

Seq data. In this paper, for each tool TOPSIS (Technique for Order of Preference by Similarity to Ideal Solution) scores were calculated by weighting four criteria i.e. sensitivity, time consumption (minutes), computational memory (RAM), and PPV (Positive Predictive Value). EricScript and FusionCatcher gained the best TOPSIS scores (0.93 and 0.87, respectively), followed by Bellerophontes and FusionMap (0.84 for both)."

I don't see how time/RAM consumption are relevant for selection of a software to pre-compute fusions for the database.

"> On the other hand, STAR-Fusion is a novel algorithm for fusion detection for which only a preprint abstract version of the paper is available at the moment (https://www.biorxiv.org/content/early/2017/03/24/120295). We would rather prefer to take advantage of peer-reviewed algorithms but we do not exclude to integrate results from other softwares in the future versions of the database."

As stated in the original paper, "STAR can discover non-canonical splices and chimeric (fusion) transcripts, and is also capable of mapping full-length RNA sequences." So the fusion detection was implemented in it from the beginning and STAR-Fusion is an extension/update of the method originally published in a peer-reviewed paper.

"> The choice of the algorithms was driven by the paper from Kumar S. et al., (Nature, 2016), which compared twelve methods for the fusion transcripts detection from RNA-Seq data. In this paper, for each tool TOPSIS (Technique for Order of Preference by Similarity to Ideal Solution) scores were calculated by weighting four criteria i.e. sensitivity, time consumption (minutes), computational memory (RAM), and PPV (Positive Predictive Value). EricScript and FusionCatcher gained the best TOPSIS scores (0.93 and 0.87, respectively), followed by Bellerophontes and FusionMap (0.84 for both). Unfortunately, the latter two softwares presented a bug when trying to annotate gene fusions on hg38 human genome version. Therefore, we chose the Tophat-Fusion algorithm which was ranked immediately after (0.74)."

Unfortunately, I find the arguments used by authors for not including results from other software tools not very convincing.

I still believe that authors should include results of additional software tools, at least for the following reasons:

1) It will demonstrate that the database can be updated in a reasonable time and will be kept up-to-date. If it is problematic to include additional software tools in the database at this stage (e.g. due to database architecture limitations), I doubt that the future database updates will be feasible.

2) Current software choice is based on just a single paper describing software comparison. Another study by Liu et al NAR 2015 (https://www.ncbi.nlm.nih.gov/pmc/articles/PMC4797269/pdf/gkv1234.pdf) gives a different ranking: SOAPfuse, FusionCatcher, JAFFA, EricScript, ... with TopHat-Fusion ranked 9th.

3) Figure 1A shows that there is little convergence/agreement between software tools. It is also not clear what choice of the software tool combination will yield optimal database search results, so the users should be provided with as many options to perform the filtering as possible.

Answer RE A.4.: We understand the reviewer's point of view when he asserts that time and RAM consumption are not strictly relevant for the selection of the softwares to use; indeed these two criteria had been previously solely considered in order to evaluate the feasibility of the tools for this big data analysis. On the other hand, the Positive Prediction Values and sensitivity had had a greater weight on the choice of the softwares.

Nevertheless, we would like to point out that Kumar et al., in the paper published on Nature in 2016, also underline that: "On the basis of Positive Prediction Values (PPV), the tools can be ordered as follows: EricScript (100%) = FusionCatcher (100%) = TopHat-Fusion (100%) > JAFFA (95.6%)". Since the main aim of LiGEA is to address researchers to validate high-confidence in-silico predicted gene fusions, we think that the Positive Predictive Value is the best fitting criterion for the choice of the algorithms to use and we are glad to see that ES, FC and TF have an excellent PPV. Therefore, we added this further argumentation to the manuscript.

We thank the reviewer for bringing to our attention the assessment performed by Liu at al. (NAR, 2015).

Since JAFFA is ranked at the 2nd position in the assessment by Kumar et al. (Nature, 2016) and, at the same time, it is also ranked 3rd in the paper by Liu et al. (NAR 2015)

Powered by Editorial Manager® and ProduXion Manager® from Aries Systems Corporation

	we have decided to integrate this fourth software to analyze, construct and systematize data into our LiGeA portal.<br>The detailed new statistics and Consensus CallSet have been reported in the revised version of the manuscript. Overall, the inclusion of a fourth software has brought to a very small reduction of the Consensus Call (~ 11%) passing from 2,828 to 2,521 extremely highly confident predictions. Not only this is a very good result indicating that the CCS is very robust, but it also provides a key for navigating the database, suggesting that also those predictions supported by at least three methods have a very good level of confidence.<br>We would like to underline that STAR is not assessed neither in Kumar's either in Liu's assessment, therefore making it difficult to justify its inclusion in our methodology. Notably, FusionCatcher relies on three aligners, including STAR, to increase the accuracy of alignment and fusion breakpoint detection. This means that the choice of STAR as the fourth software would have probably added several redundant results to our analysis, thus losing the important goal underlined both by the editor and the reviewer about increasing the accuracy of the Consensus Call Set.<br>As regards STAR-Fusion, we downloaded and used the pre-built indexes provided with the software and we tried to run it on a sub-sample of our dataset. Unfortunately, while STAR-fusion correctly ran and produced correct outputs when performed on the test files provided by the authors of the software, the output files were completely empty when running STAR-fusion on the cell lines' fastq files. It seems this is not the first time it happens, according to other open issues we found on forums dedicated to STAR-Fusion, probably due to a problem related to plug and play version of the genome resource lib released by the authors (https://github.com/STAR-Fusion/STAR-Fusion/issues/2).<br>Unfortunately, at the state of the art, STAR-Fusion does not seem to be very robust, therefore making it impossible for us to use since, also considering the importance of the topic, we want to provide highly reliable predictions.<br>It took less than two months, most of which spent running the algorithm, to seamlessly integrate the results from a fourth software, thus demonstrating that future updates of the database would not be problematic at all. We will thus be glad to integrate the results from STAR-Fusion as soon as it will be published and released in a more stable version.<br><br><br>####################Point by point answers to Reviewer#2:<br>Major Points:<br>1. First, in "Search by" tab, when I tried to input ALK gene in 3' partner, it is taking some time to load gene list and then select it. People usually get user's input and then search their database to return the results. Please consider what would be better to get user input.<br>A1. We have slightly modified the behaviour of our search pages. Now all the autocomplete fields accept any text which has been typed in (regardless of the fact that the gene list has been already loaded or not). Also, as before, they also keep listing the matching candidates known to the system.<br><br>2. In Statistics page, please provide with color legends on colorful pie chart. (now it gives mouse-over legends).<br>A2. The legend has been changed as suggested.<br><br>Minor points:<br>3. All the gene symbols are upper characters. for example, C9ORF66 gene, it is actually C9orf66 according to HGNC. I think authors did for their conveniences, but I think the gene symbols should follow HGNC nomenclature. Please compare and update your gene symbol according to latest version of HGNC and their aliases. It will be better to give official gene symbol and aliases. For example, official symbol of well-known HER2 gene is ERBB2. People tends to assume that all gene symbols are upper characters, but NOT.<br>A3. Thank you for pointing out this important issue. Now the gene symbols follow the latest HGNC nomenclature.<br><br>4. In each link, please give "EXACT" links rather than main page. for example, I noticed that a link for ALK gene is http://cancer.sanger.ac.uk/cosmic/census but it SHOULD be pointing to http://cancer.sanger.ac.uk/cosmic/gene/analysis?ln=ALK as

	well as genecards http://www.genecards.org/cgi-bin/carddisp.pl?gene=ALK&keywords=ALK<br>Please find all links and give their EXACT url links, NOT top page.<br>A4.: The gene ALK points to two different pages under COSMIC because ALK is a Cosmic Gene and, at the same time, it also belongs to the subset of the 719 genes encompassed in Cosmic Gene Census list. Therefore, this link http://cancer.sanger.ac.uk/cosmic/gene/analysis?ln=ALK brings to the specific web page dedicated to ALK, while this one http://cancer.sanger.ac.uk/cosmic/census points to the page containing the table with all the Census genes (this is not a top page). Nevertheless, we noticed that this information was not described very clearly in the manuscript, therefore we rewrote some sentences in the paragraph dedicated to Cosmic Gene Census. Furthermore, we downloaded and updated the newly released list (from 699 genes during the previous round of revision to 719 genes at the current date) and computed the new statistics.<br><br>5. In fusion gene info, you used NPM1#ALK but in each info page, you used NPM1/ALK. Please use same separator.<br>A5. Thank you for pointing out this discrepancy. Now we use always the same separator.<br><br>6. In each search page, can you delete mouse-over message "this fusion event is supported by...." I don't think this long message is giving valuable information. It is already under "FusionCatcher", "EricScript" and "Tophat-fusion". And I noticed that each icons are linked to same page, not even pubmed icon. I thought pubmed Icon can lead me to pubmed but NOT.<br>A6: As suggested, we removed the mouse-over message:"this fusion event is supported by…". As regards the icons, they are a graphical representation of the systematization of the results, this is explained in the legend posed upon the table of results. On the other hand, each predicted gene fusion event has a unique ID, pointing to a dedicated webpage where these icons are linked to the respective external resources.<br><br>7. Please give thorough examination on each page again, menu, and etc. I thought that you have answered all questions but I can still found many errors.<br>A7: We had another round of examination over each page. Thank you.<br><br>##########################Answer to Reviewer#3:<br>An useful article and database of fusion genes in human cancer cell lines for research community.<br>A: Thank you.
**Additional Information:**	
**Question**	**Response**
Are you submitting this manuscript to a special series or article collection?	No
**Experimental design and statistics**<br><br>Full details of the experimental design and statistical methods used should be given in the Methods section, as detailed in our Minimum Standards Reporting Checklist. Information essential to interpreting the data presented should be made available in the figure legends.<br><br>Have you included all the information requested in your manuscript?	Yes

Powered by Editorial Manager® and ProduXion Manager® from Aries Systems Corporation

**Resources**<br><br>A description of all resources used, including antibodies, cell lines, animals and software tools, with enough information to allow them to be uniquely identified, should be included in the Methods section. Authors are strongly encouraged to cite Research Resource Identifiers (RRIDs) for antibodies, model organisms and tools, where possible.<br><br>Have you included the information requested as detailed in our Minimum Standards Reporting Checklist?	Yes
**Availability of data and materials**<br><br>All datasets and code on which the conclusions of the paper rely must be either included in your submission or deposited in publicly available repositories (where available and ethically appropriate), referencing such data using a unique identifier in the references and in the "Availability of Data and Materials" section of your manuscript.<br><br>Have you have met the above requirement as detailed in our Minimum Standards Reporting Checklist?	Yes

Manuscript

Click here to download Manuscript 2nd-Revision_LiGeA.pdf

GigaScience, 2017, 1–8

doi: xx.xxxx/xxxx
Manuscript in Preparation
Data Note

DATA NOTE

Massive NGS Data Analysis Reveals Hundreds Of Potential Novel Gene Fusions in Human Cell Lines

Silvia Gioiosa[1,4,†], Marco Bolis[2,†], Tiziano Flati[1,4], Annalisa Massini[3], Enrico Garattini[2], Giovanni Chillemi[1], Maddalena Fratelli[2,*] and Tiziana Castrignanò[1,*]

[1]SCAI-Super Computing Applications and Innovation Department, CINECA, Rome, Italy, and [2]Laboratory of Molecular Biology, IRCCS-Istituto di Ricerche Farmacologiche “Mario Negri,” Milano, Italy, and [3]Computer Science Department, Sapienza University of Rome, Italy, and [4]National Council of Research, CNR, Institute of Biomembranes, Bioenergetics and Molecular Biotechnologies, Bari, Italy.

[†]Contributed equally.
[*] To whom correspondence should be addressed: t.castrignano@cineca.it; maddalena.fratelli@marionegri.it

Abstract

Background: Gene fusions derive from chromosomal rearrangements and the resulting chimeric transcripts are often endowed with oncogenic potential. Furthermore, they serve as diagnostic tools for the clinical classification of cancer subgroups with different prognosis and, in some cases, they can provide specific drug targets. So far, many efforts have been carried out to study gene fusion events occurring in tumor samples. In recent years, the availability of a comprehensive Next Generation Sequencing dataset for all the existing human tumor cell lines has provided the opportunity to further investigate these data in order to identify novel and still uncharacterized gene fusion events.

Results: In our work, we have extensively reanalyzed 935 paired-end RNA-seq experiments downloaded from "The Cancer Cell Line Encyclopedia" repository, aiming at addressing novel putative cell-line specific gene fusion events in human malignancies. The bioinformatics analysis has been performed by the execution of four different gene fusion detection algorithms. The results have been further prioritized by running a bayesian classifier which makes an *in silico* validation. The collection of fusion events supported by all of the predictive softwares results in a robust set of ~ 1,700 *in-silico* predicted novel candidates suitable for downstream analyses. Given the huge amount of data and information produced, computational results have been systematized in a database named LiGeA. The database can be browsed through a dynamical and interactive web portal, further integrated with validated data from other well known repositories. Taking advantage of the intuitive query forms, the users can easily access, navigate, filter and select the putative gene fusions for further validations and studies. They can also find suitable experimental models for a given fusion of interest.

Conclusions: We believe that the LiGeA resource can represent not only the first compendium of both known and putative novel gene fusion events in the catalog of all of the human malignant cell lines, but it can also become a handy starting point for wet-lab biologists who wish to investigate novel cancer biomarkers and specific drug targets.

Key words: Database; Human gene fusions; Malignant Cell Lines; NGS; Gene Fusion detection algorithms; Chromosomal rearrangements; Bioinformatics

Compiled on: March 29, 2018.
Draft manuscript prepared by the author.

Key Points

- A massive bioinformatics analysis conducted on Paired-End RNA-seq samples from 935 human malignant Cell Lines reveals a landscape of known and novel *in-silico* predicted gene fusion events;
- LiGeA Portal represents a user-friendly database for the systematization, visualization and interrogation of the results;
- LiGeA Portal is further integrated with information from other databases and with gene-fusion priotirization analysis, in order to address targeted experimental validations on a highly reliable set of candidate gene fusions.

Background

Oncogenic gene fusion events result from chromosomal rearrangements which lead to the juxtaposition of two previously separated genes. The accidental joining of DNA of two genes can generate hybrid proteins. It can also result in the misregulation of the transcription of one gene by the *cis-regulatory* elements (promoters or enhancers) of another, sometimes resulting in the production of oncoproteins that bring the cell to a neoplastic transformation (Mitelman et al.; 2007). Not only gene fusions can have a strong oncogenic potential (Mertens et al.; 2015), but they also serve as diagnostic tools for the clinical classification of cancer subgroups with different prognosis and, in some cases, they may provide specific drug targets (Seratì et al.; 2016). For instance, the presence of the PLM-RARA fusion product is a specific hallmark of acute promyelocytic leukemia (APL) (Borrow et al.; 1990) and represents the first example of gene-fusion targeted therapy (Nervi et al.; 1998) that has changed the natural history of this disease. Hence, there are several reasons why studying gene fusions in cancer is very important. In recent years, Next-Generation Sequencing (NGS) technologies have played an essential role in the understanding of the altered genetic pathways involved in human cancers. Nowadays, most of the studies aiming at fusion discovery use NGS techniques followed by massive bioinformatics analyses. The greatest challenge of these sophisticated algorithms of prediction is the ability to discriminate between artifacts and really occurring chromosomal rearrangements (Lou et al.; 2009). Moreover, each gene fusion predicting software differs in terms of sensitivity and specificity. In the last decade, much effort has been done to catalog gene fusion events, thus resulting in a wide production of databases. At present, a dozen of published databases regarding oncogenic fusion genes exists (see table 1 for a summary). Some of them (e.g. FusionCancer, ChiTaRS-3.1) collect *in silico* predictions of chimeric genes, obtained analyzing publicly available datasets derived from heterogeneous sources either in terms of experimental material (a mix of Single-End and Paired-End RNA-seq data, ESTs) and in terms of data source (patients and cell lines). Some others collect gene fusion events with experimental evidences manually curated from literature collection (e.g. TCGA, Mitelman, TICdb, COSMIC, ONGene). In this work we focused on the whole catalog of Human malignant Cell Lines, thus obtaining a homogeneous input NGS dataset covering several human malignancies. We exerted a massive bioinformatics analysis on 935 paired-end RNA-seq samples derived from 22 different tumor tissues and used a combination of the best performing gene fusion-detecting algorithms. For ease of understanding, we define the predicted Gene Fusion Event (pGFE) as the entity constituted by the gene fusion couple in a specific cell line and designate the Consensus Call-Set (CCS) as the number of pGFEs supported by all the used algorithms. Starting from this assumption, we obtained a total of 377,540 pGFEs, 2,521 of which belonging to the CCS. Moreover, since not all the pGFEs can give rise to oncogenic transformations, the use of a priotirization software is recommended in order to distinguish between real driver mutations from passenger ones. Therefore, a robust Bayesian classifier has been used to perform an *in silico* validation of the results. Since one of the main purposes of this big data analysis is encouraging the reuse of our results in order to experimentally validate the *in-silico* predictions, we set up a web portal collecting and systematizing these data, LiGeA (cancer cell LInes Gene fusion portAl). It is possible to browse, search and freely download all the results obtained and described within this article at the LiGeA repository web page available at http://hpc-bioinformatics.cineca.it/fusion/. To our knowledge, our resource represents the first compendium of both known and predicted novel gene fusion events in cell lines from 22 different human tumor types.

Data Description

Methods

We have analyzed 935 paired-end RNA-seq experiments available at the Cancer Cell Line Encyclopedia repository, for a total of 32 TB of input raw data. The analysis has been carried out by using four different somatic fusion gene detection algorithms: FusionCatcher (Nicorici et al.; 2014), EricScript (Benelli et al.; 2012), Tophat-Fusion (Daehwan and Salzberg; 2011) and JAFFA (Davidson et al.; 2015). The choice of the algorithms was driven by the assessment from Kumar S. et al. (Kumar et al.; 2016), which compared twelve methods for the fusion transcripts detection from RNA-Seq data and identified these softwares as the ones with the highest Positive Prediction Values. Furthermore, the chosen softwares differ in a variety of aspects and contain several layers of information in their output files, thus giving us the opportunity to collect and interconnect a wide set of complementary data for each pGFE. Here is a short description of each fusion detection tool, accompanied by the used versions and parameters.

- **FusionCatcher (FC)**: FC is a Python based algorithm. It executes a first mapping run with Bowtie v.1.2.0 (Langmead et al.; 2009) and then performs the Gene fusion detection basing on three different aligners: Bowtie2 v.2.2.9 (Langmead and Salzberg; 2012), BLAT v.36 (Kent; 2002) and STAR v.2.5.2b (Dobin et al.; 2013). FC takes advantage of NCBI Viral Genomes (v. 2016-01-06) in order to detect exogenous virus material integration into the host genome. Moreover, the FC algorithm compares its own output with a set of published databases, thus proving a detailed list of truly positive and false positive pGFEs candidates. In our analysis we downloaded FC v. 0.99.5a and Ensembl genome annotation v.83 and used hg38/GRCh38 as genome assembly version. The software was executed with default parameters, requiring 111,620 CPU core hours, 125 GB of RAM and 20 CPUs to complete the execution on our input dataset. Overall, FC detected 25,251 pGFEs involving 8,659 genes.
- **Tophat-Fusion (TF)**: TF uses the Tophat-fusion-post function in order to create a filtered list of gene fusion can-

Table 1. State of the art of databases reporting gene fusions

Database Name	URL	Short Description
Tumor Fusion Gene Data Portal	http://54.84.12.177/PanCanFusV2//	A collection of fusion genes in the Tumor Cancer Genome Atlas (TCGA) samples.
TICdb (Novo et al.; 2007)	http://www.unav.es/genetica/TICdb/	A collection of 1,374 fusion sequences extracted either from public databases or from published papers (last update: 2013).
chimerDB3.0 (Lee et al.; 2017)	http://203.255.191.229:8080/chimerdbv31/mindex.cdb	A catalog of fusion genes encompassing analysis of TCGA data and manual curations from literature.
ONGene (Liu et al.; 2017)	http://ongene.bioinfo-minzhao.org/	Literature-derived database of oncogenes
COSMIC Cell Lines	http://cancer.sanger.ac.uk/cell_lines	Gene fusions are manually curated from peer reviewed publications. Currently COSMIC includes information on fusions involved in solid tumors but not yet leukemias and lymphomas.
Mitelman (Mitelman et al.; 2007)	https://cgap.nci.nih.gov/Chromosomes/Mitelman	Reports hundreds of gene fusions associated with clinical reports but does not contain sequence data.
ChiTaRs-3.1 (Gorohovski et al.; 2017)	http://chitars.md.biu.ac.il/index.html	A collection of 34,922 chimeric transcripts identified by Expressed Sequence Tags (ESTs) and mRNAs from the GenBank, ChimerDB, dbCRID, TICdb and the Mitelman collection of cancer fusions for several organisms.
FusionCancer (Wang et al.; 2015)	http://donglab.ecnu.edu.cn/databases/FusionCancer/	591 samples, both single-end and paired-end RNA-seq, published on SRA (http://www.ncbi.nlm.nih.gov/sra) database between 2008 and 2014 covering 15 kinds of human cancers .

didates, starting from the output files obtained running Tophat with the "-fusion-search" option (Trapnell et al.; 2009)." The following commands were run subsequently:

```
tophat -o $Sample.output/ -p 20 -fusion-search -keep-fasta-order -bowtie1 -no-coverage-search -r 160 -mate-std-dev 34 -max-intron-length 100000 -fusion-min-dist 100000 -fusion-anchor-length 13 $BOWTIE_INDEX/hg38 $Sample_1.fastq $Sample_2.fastq

cd $Sample.output/

tophat-fusion-post -p 20 -skip-blast $BOWTIE_INDEX/hg38
```

Tophat-2.0.12 and samtools 0.1.19 versions were used for this study. This algorithm took about 200,000 CPU core hours, 20 CPUs and 125 GB of RAM in order to complete its runs on the whole input dataset. TF produces several output files but only the file named "results.txt", representing the filtered list of predicted gene fusions, was used for subsequent analysis. The results encompassing "Chromosome M" have been manually discarded from the final results, *in primis* because TF and JF were the only ones of the four algorithms reporting them, secondly because they represented *bona-fide* false positive outcomes. Overall, TF highlighted 28,146 pGFEs involving 9,492 genes.

- **JAFFA (JA)**: JAFFA (v. 0.9) is a multi-step pipeline that takes raw RNA-Seq reads and outputs a set of candidate fusion genes along with their cDNA breakpoint sequences. It relies on trimmomatic (Bolger et al.; 2014), samtools (Li et al.; 2009), BLAT (Kent; 2002), bowtie2, bpipe (Sadedin et al.; 2012) and R softwares (R Development Core Team; 2008) as well as on gencode (v. 22) for the annotation and on Mitelman database for flagging already known gene fusions. For the purpose of this analysis, we used the "Direct" mode pipeline which is indicated for reads of 100 bp or longer. A total amount of 1,300,000 CPU core hours, 125 GB of RAM and 20 CPUs were required to successfully complete the analysis. The results encompassing "Chromosome M" have been manually discarded from the final results. Furthermore, only pGFEs supported by at least 3 spanning reads or flagged as "known", have been retained. Overall, after the filtering process, JA detected 53,400 pGFEs involving 12,256 genes.
- **EricScript (ES)**: ES is developed in R, perl and bash scripts. It uses the BWA aligner (Li and Durbin; 2009) to perform the mapping on the transcriptome reference and samtools v. 0.1.19 to handle with SAM/BAM files. Recalibration of the exon-junction reference is performed by using BLAT. For the purposes of this project, BLAT v.36 was downloaded at http://genome-test.cse.ucsc.edu/~kent/exe/linux/. Moreover, it was necessary to download R v.3.3.1 and a bedtools version greater than 2.20 (here we used v. 2.24). For this study, ES version 0.5.5 was obtained at https://sourceforge.net/projects/ericscript/files/. The Ensembl Database v. 84 was downloaded from https://docs.google.com/uc?id=0B9s__vuJPvIiUGt1SnFMZFg4T1E&export=download and built locally using BWA software with the command:

```
bwa index -a bwtsw allseq.fa
```

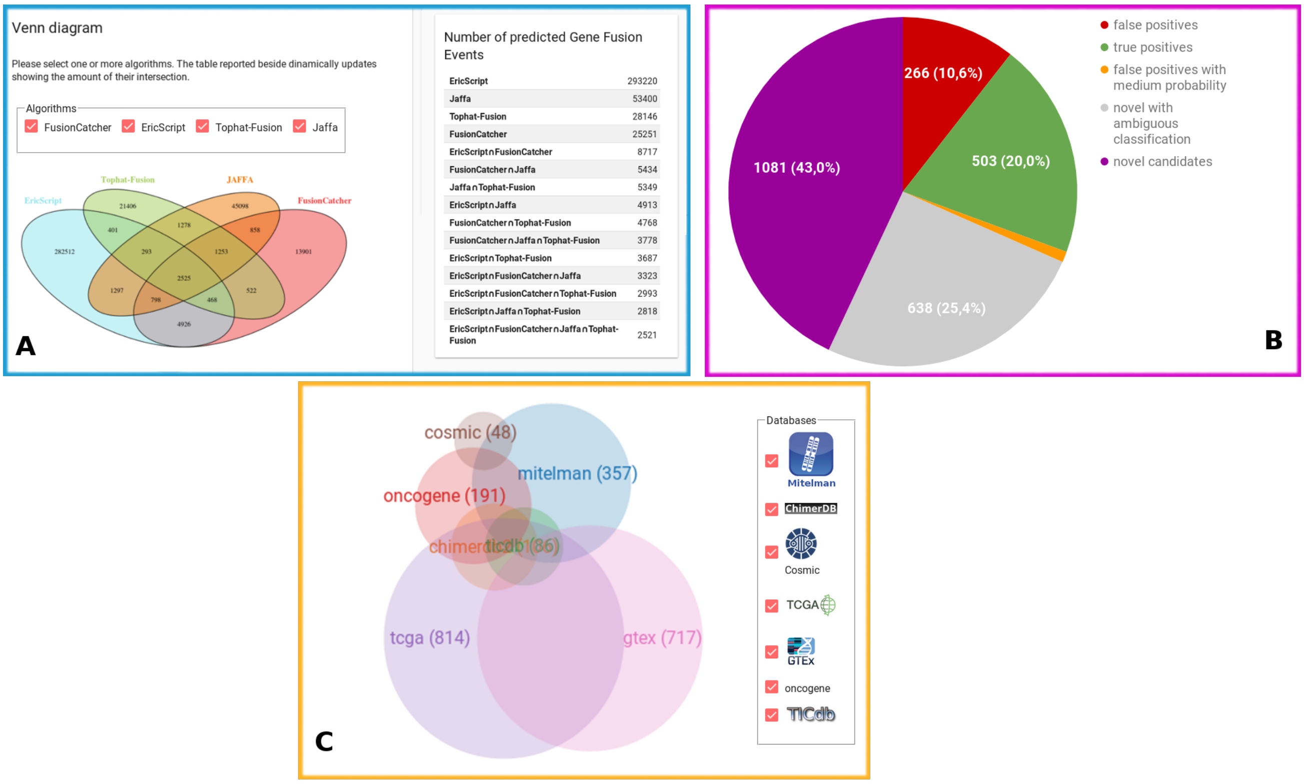

Figure 1. a) Venn diagram showing the intersection of the pGFEs identified by the four algorithms. b) Distribution of pGFEs in the Consensus Call-set: 43% (purple) of the CCS has not been previously described in any other database or scientific publication; 10% (red) and 20% (green) of the CCS have been reported in databases from healthy/tumoral samples thus representing the false/true positive subset of our analysis; 1% of the CCS (orange) reports tags which classify the pGFE as a false positive couple with medium probability; 25% (grey) of the results represent novel pGFEs tagged with values which classify them as both false and true positives. c) Venn diagram showing the intersection between the LiGeA CCS and other databases.

A total amount of 130,900 CPU core hours, 125 GB of RAM and 20 CPUs were required to successfully complete the analysis. We further filtered out ES final results by removing all the predictions for which the software was not able to predict an exact breakpoint position because such pGFEs could not even be experimentally validated. Secondly, as also applied to FC, TF and JF's results, we retained the pGFEs exhibiting at least 3 spanning reads over the gene fusion junction. Furthermore, we filtered out all the pGFEs with EricScore value less than 0.85. EricScore is a ranking parameter ranging from 0.5 to 1: greater values correspond to better predictions. Interestingly, by applying these filters, we filtered out almost 2/3 of the initial predictions from EricScript but, at the same time, the CCS did not reduce substantially, thus indicating that the choice of a consensus of predictions is a good strategy to remove false positives and obtain a reliable set of gene fusion candidates to be experimentally validated. Overall, after the filtering process, ES detected 293,220 pGFEs involving 14,740 genes.

Data Statistics and Validation

Overall, our extensive analysis results in a CCS of 2,521 pGFEs (Fig. 1A) and respectively 2,828/9,258 pGFEs supported by exactly three/two methods. As a first validation of our analysis, 661 out of the 719 (92%) genes known to be functionally implicated in cancer and collected under COSMIC gene census, are present in our final dataset. As a further validation of our results, about 1/5 of our CCS has already been published or is present in the following databases: chimerdb3; ONGene; COSMIC; tcga; ticdb; Mitelman (Fig. 1C). Finally, only a small subset of the pGFEs (~10% of data) present in the CCS have been recognized as false positive predictions, thus supporting the idea that a combination of algorithms can be of great utility in order to increase the sensitivity and the specificity of the tests. It is worth mentioning that, not only our analysis confirmed a large number of known gene fusion events, but it also highlighted 1,719 novel putative pGFEs in the CCS which could undergo further downstream analysis (Fig. 1B). Therefore, a further step of analysis was run with Oncofuse v.1.1.1 (Shugay et al.; 2013) in order to distinguish driver mutations (genomic abnormalities responsible for cancer) from passenger ones (inert somatic mutations not implicated in carcinogenesis). Oncofuse is considered an *in silico* validation post-processing step which prioritizes the results obtained from each of the three algorithms. It assigns a functional prediction score to each putative fusion sequence breakpoint identified by the four softwares thus hinting which pGFEs are worthy of being experimentally validated and studied. Oncofuse supports multiple input formats such as the output from TF and FC. In order to run it also on the outputs from ES and JF, a short pre-processing step was executed on these data. As suggested on Oncofuse manual, the accepted default input format is a tab-delimited file with lines containing 5' and 3' breakpoint positions. Therefore, these columns were extracted from ES and JF output files and redirected into Oncofuse accepted input format. Oncofuse was run with default parameters using hg38 as the reference genome.

Availability of supporting data and materials

The datasets obtained and described within this article are freely downloadable at the LiGeA repository available at http:

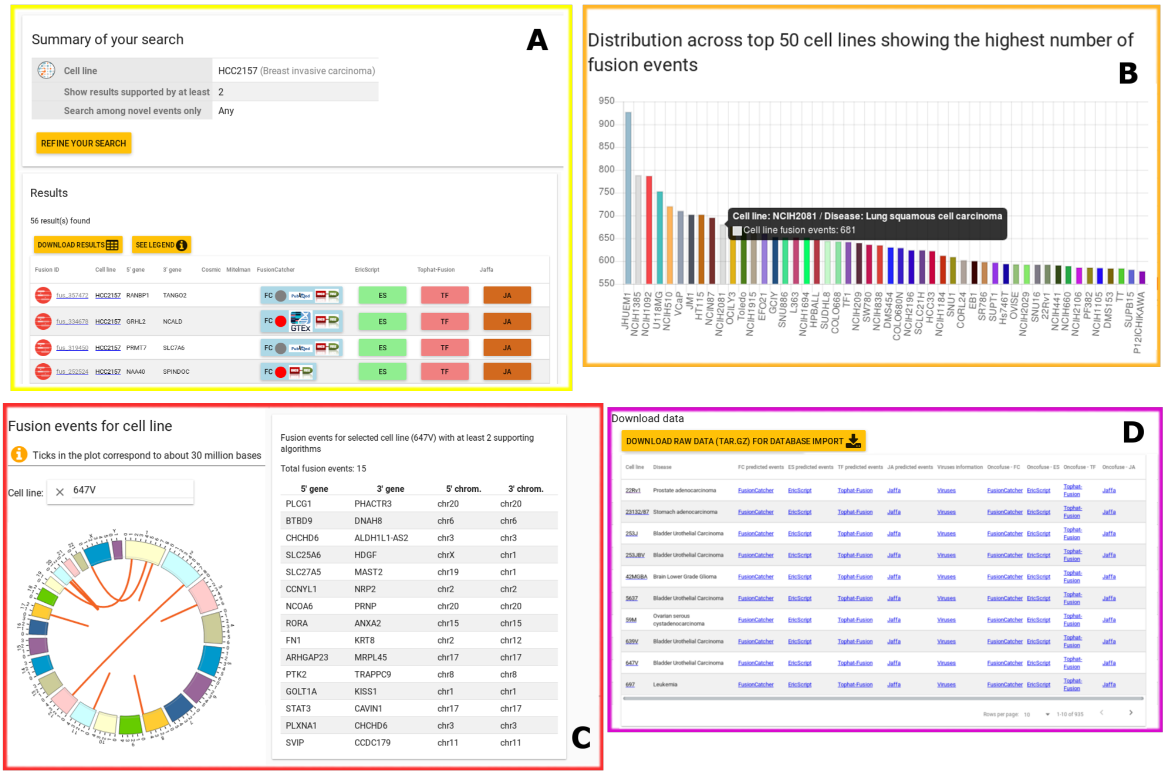

Figure 2. An overview of LiGeA portal. a) A 'Search by Cell line' example and the corresponding output; b) An overview of the input dataset; c) A circos diagram showing the graphical outcome of a 'Query by cell line' and the corresponding related table; d) An extract from the 'Download' web page.

//hpc-bioinformatics.cineca.it/fusion/downloads.

Database Description

LiGeA is a database server based on graph-db technology (Neo4j). The portal stores all of the results obtained from each fusion gene predicting algorithm and the prioritization analysis outcome. Anyway, this database contains not only a mere collection of *in silico* predictions. Indeed, it has been integrated with other useful external resources in order to offer a carefully-curated web compendium. Here is a short list of the added features:

- Whenever the gene fusion couple has already been experimentally validated and published, an extra column with COSMIC icon is added to the results. By clicking on it, the user will be redirected to an external link containing a manually-curated catalog of 212 literature-derived somatic mutations in cancer (COSMIC; 2017a);
- Cancer Gene Census is a manually curated catalog of 719 genes for which mutations have been causally implicated in oncogenesis (Futreal et al.; 2004). Whenever one of the two genes involved in the pGFE has been already described to be implicated in cancer, the gene is tagged with an icon. By clicking on it, an external link to the Cancer Gene Census is provided showing a table of genes included within this category (COSMIC; 2017b).
- A legend based on a colorful signature has been added to tag the FC predictions as 'validated truly positive couples' (green circle), 'validated false positive couples' (red circle), 'false positive couples with medium probability' (orange circle) and 'ambiguous signature' because tagged with both positive and negative values (grey circle) ;
- A functional prediction score obtained by extensively running the Oncofuse software, is reported as additional tag to the outputs from each algorithm.

LiGeA portal is divided into several sections which allow a user-friendly navigation.

- **Home:** In the homepage, the user is provided with a quick overview of the database. A global summary table reports a numeric recapitulation (e.g. the number of genes/transcripts/exons collected into the portal; the number of predicted proteins and so on). Moreover, a histogram shows an abstract of the top 50 involved cell lines. By moving the cursor on the bars, a pop-up opens showing the cell line name and the corresponding number of the unique fusion events predicted by all the algorithms. Information about the algorithm predictions hosted into the portal are supplied with an interactive Venn Diagram linked to a dynamical table. Upon user selection of the algorithm/s of interest, both the diagram and the table refresh thus showing the resulting number of intersections.

- **Search:** This utility allows several searching options to browse and mine genomic-fusion events stored in LiGeA portal (see table 2 for an overview). All the resulting outputs are sorted by the number of algorithms supporting the fusion events, thus showing on the top of the table the most robust set of results. As additional feature, when specifying the features of interest, it is also possible to choose the minimum number of predicting algorithms. Search results are presented in the form of a paginated table containing those fusion events which satisfy the query parameters and data can also be downloaded in tabular format. Furthermore, by clicking on a given fusion ID, it is possible to access the event-specific page in which relevant information is presented in greater detail (e.g., involved cell line, dis-

ease, genes as well as links to external databases and resources). Two out of nine of the query forms ('search by fusion information' and 'search by virus') are specific annotations derived FC algorithm. Here is a short description of the provided searching utilities.

- 'Search by Disease': In this section, all the cell lines derived from the same disease have been grouped together. In this way, it is possible to navigate the gene fusions putatively causing specific malignancies. The number of the cell lines constituting the queried subset is shown besides the pathology name.
- 'Search by Cell Line': This module allows to navigate the database by indicating a specific cell line name. It is possible to tune the results by showing only the novel predictions not yet described in any other database or publication (Fig.2A).
- 'Search by Chromosome': This query can be performed by inserting one or two chromosomes involved in the fusion event. The cell line name can be either indicated or not.
- 'Search by Gene': the user can select up to two gene names (Gene Symbol or ENSEMBL ID) and the 'cell line' form can be either selected or not. The genes reported in the query form are black if they are involved in pGFE and gray if they are not.
- 'Search by Transcript': Since the same gene can give rise to different transcripts, it could be reasonable to query which of the transcripts produced by a specific gene are affected by a fusion event. This kind of query can be satisfied by inserting the Ensembl Transcript (ENST) IDs in the specific form.
- 'Search by Exon': Some of the queries allow to go much more into molecular detail. This search can be done by inserting one or two exon IDs involved in the fusion event. The cell line name can be either indicated or not. In this way it is possible to highlight the specific exons which turn out to be fused in the final result.
- 'Search by Fusion information': The pGFEs may have different predicted effects. Indeed, depending on the location of the chromosomal break points, the resulting protein may be in-frame, out-of frame, truncated and so on. Since the selectable values present in the fusion information form are specific of FC algorithm, the result of this query returns a table without ES,JA and TF data. We suggest to view this section of FC manual https://github.com/ndaniel/fusioncatcher/blob/master/doc/manual.md#62---output-data-output-data in order to obtain a full description of all of the tags.
- 'Search by Algorithm': this type of query is suitable for users who wish to navigate the outputs from specific softwares, choosing them individually or in combination. Indeed, it is known that some kind of fusions, such as those involving immunoglobulins, can be detected by specific softwares (Reshmi et al.; 2017).
- 'Search by Viruses': Another useful information retrievable from the database regards virus sequence integration into the host genome. This search utility is virus-centered since it is possible to indicate or not the host cell line name. It is possible to select the virus name of interest (whether using GI ID or NC ID). Furthermore, a clickable link redirecting to the virus genome is also shown on the right of the table.

- **Statistics:** this section allows a visual inspection of the results. The four sub-menus are organized as follows:
  - 'Cell Line Statistics': by choosing the Cell Line of interest, the resulting circular diagram shows all the chromosome couples involved in GFE predicted by at least two algorithms. The table on the right summarizes the resulting couples of the genes and chromosomes (Fig. 2C).
  - 'Chromosome Statistics': this page reports a dynamical pie-chart showing the number of fusion events per human chromosome; by clicking on each slice of the pie, the related table automatically updates showing a chromosome summary statistics. Furthermore, information about the number of inter- and intra-chromosomal rearrangements detected by each algorithm is also reported.
  - 'Disease Statistics': The 'Fusion Statistics' pie-chart was produced by grouping together the cell lines derived from the same human pathology thus showing the total number of fusion events normalized by the number of cell lines composing a specific disease. The 'Virus statistics panel' shows the frequency of exogenous virus integration per human malignancy.
  - 'Gene Statistics': A word cloud diagram showing the most recurring pGFEs supported by three methods.
  - 'Database Statistics': This sub-section is composed by four panels, the first regarding data in the CCS (Fig. 1B), the others relating only to FC and JA results. In this page it is possible to get information about the number of pGFEs found in known databases (visualized as interactive Venn diagrams and tabular fashion) and the distribution of predicted effects (histogram view).

- **Dataset:** This page is a description of the input dataset used for the analysis. Among the above 1000 samples available at Broad institute portal CCLE repository, we downloaded 935 PE RNA-seq in fastq format. The SE samples have been discarded since the used softwares required it. The histogram in this section shows the number of the different cell lines derived from the same diseases (Fig. 2B). Furthermore, starting from this section, it is possible to access to web pages resuming cell-line specific details (e.g. COSMIC ID, drug resistance, human disease among others) .

- **Downloads:** From this panel it is possible to download all the processed data described within this article (Fig. 2D). Some of the files ('Summary information' and 'Viruses information') are specific products of FusionCatcher algorithm.

Availability and Requirements

- **Project name**: LiGeA: a comprehensive database of human gene fusion events
- **RRID**: SCR_015940
- **Project home page**: http://hpc-bioinformatics.cineca.it/fusion (GitHub project: https://github.com/tflati/fusion)
- **Operating system(s)**: Any
- **Programming language**: Python, JavaScript+HTML+CSS
- **Other requirements**: Django 1.10.5, Python 2.7.12, AngularJS 1.5.11
- **License**: GNU GPLv3

Declarations

List of abbreviations

LiGeA: cancer cell LInes GEne-fusions portAl; pGFE: predicted Gene Fusion Event; NGS: Next Generation Sequencing; TCGA: Tumor Cancer Genome Atlas; SRA : Sequence Read Archive; APL: acute promielocytic leukemia; CCS: Consensus Call-Set; FC: FusionCatcher; ES: EricScript; TF: Tophat-Fusion; JA: JAFFA.

Table 2. Example of possible queries on LiGeA portal

Search by	Question	Query
Disease	’what are the fusion events present in stomach adenocarcinoma cell lines?’	Select ’stomach adenocarcinoma’ under ’disease’ menu
Cell Line	’what are the novel putative pGFEs affecting RH30(Sarcoma) cell line?’	Select ’RH30’ under the cell line menu and check the box ’show only novel results’
Chromosome	’what are the most suitable fusion partners for chromosome 8?’	Select ’Chr8’ either under the ’5’ Chromosome’ or under the ’3’ Chromosome’ tab and leave blank the other forms
Gene	’how many human cell lines show the PML-RARA fusion event?’	Select ’PML’ under the ’5’ gene menu’; Select ’RARA’ from the ’3’ gene menu’; leave blank the ’Cell Line’ query form;
Fusion information	’what are all the in-frame pGFEs in Jurkat cell line?’	select ’Jurkat’ under ’Cell line’ menu; Select ’in-frame’ under ’predicted effect menu
Fusion information	’what are the known GFEs predicted to be in-frame in Jurkat cell line?’	Select ’Jurkat’ under ’Cell line’ menu; Select ’in-frame’ under ’predicted effect menu; select ’known’ under ’Fusion description’ menu
Algorithm	’show only those GFEs supported by FC and TF in RH30 cell line’	Select ’RH30’ under ’Cell Line’ query form and check the boxes relative to FC and TF
Viruses	’which cell lines are most affected by Hepatitis C virus genome integration?’	Select ’Hepatitis C virus’ under ’Virus’ query form and let blank the ’Cell line’ query form

Consent for publication

‘Not applicable’

Competing Interests

The authors declare that they have no competing interests.

Funding

This work was supported by ELIXIR-IIB, CINECA and Regione Lombardia.

Author’s Contributions

TC and MF conceived and designed the work. All authors analyzed, interpreted data, wrote the manuscript and approved the final manuscript.

Acknowledgements

We acknowledge Andrea Micco for his useful tests on the first prototype of the system. We acknowledge the CINECA and the Regione Lombardia award under the LISA initiative 2016-2018, for the availability of high performance computing resources and support.

References

Benelli, M., Pescucci, C., Marseglia, G., Severgnini, M., Torricelli, F. and Magi, A. (2012). Discovering chimeric transcripts in paired-end rna-seq data by using ericscript, *Bioinformatics* **28**(24): 3232–3239.
URL: *http://dx.doi.org/10.1093/bioinformatics/bts617*

Bolger, A. M., Lohse, M. and Usadel, B. (2014). Trimmomatic: a flexible trimmer for illumina sequence data, *Bioinformatics* **30**(15): 2114–2120.
URL: *+ http://dx.doi.org/10.1093/bioinformatics/btu170*

Borrow, J., Goddard, A., Sheer, D. and Solomon, E. (1990). Molecular analysis of acute promyelocytic leukemia breakpoint cluster region on chromosome 17, *Science* **249**(4976): 1577–1580.
URL: *http://science.sciencemag.org/content/249/4976/1577*

COSMIC (2017a). Cosmic database-wellcome trust sanger institute @ONLINE.
URL: *http://cancer.sanger.ac.uk/cosmic*

COSMIC (2017b). Cosmic gene census - wellcome trust sanger institute @ONLINE.
URL: *http://cancer.sanger.ac.uk/census*

Daehwan, K. and Salzberg, S. (2011). Tophat-fusion: An algorithm for discovery of novel fusion transcripts, *Genome Biology* **12**(8).

Davidson, N. M., Majewski, I. J. and Oshlack, A. (2015). Jaffa: High sensitivity transcriptome-focused fusion gene detection, *Genome Medicine* **7**(1): 43.
URL: *https://doi.org/10.1186/s13073-015-0167-x*

Dobin, A., Davis, C. A., Schlesinger, F., Drenkow, J., Zaleski, C., Jha, S., Batut, P., Chaisson, M. and Gingeras, T. R. (2013). Star: ultrafast universal rna-seq aligner, *Bioinformatics* **29**(1): 15–21.
URL: *http://dx.doi.org/10.1093/bioinformatics/bts635*

Futreal, P. A., Coin, L., Marshall, M., Down, T., Hubbard, T., Wooster, R., Rahman, N. and Stratton, M. R. (2004). A census of human cancer genes., *Nature reviews Cancer* **4**(3): 177–183.

Gorohovski, A., Tagore, S., Palande, V., Malka, A., Raviv-Shay, D. and Frenkel-Morgenstern, M. (2017). Chitars-3.1—the enhanced chimeric transcripts and rna-seq database matched with protein–protein interactions, *Nucleic Acids Research* **45**(D1): D790–D795.
URL: *+ http://dx.doi.org/10.1093/nar/gkw1127*

Kent, W. (2002). Blat—the blast-like alignment tool., *Genome Research* **12**(4): 656–664.

Kumar, S., Vo, A. D., Qin, F. and Li, H. (2016). Comparative assessment of methods for the fusion transcripts detection from rna-seq data, *Nature Scientific Reports* **6**.

Langmead, B. and Salzberg, S. (2012). Fast gapped-read alignment with bowtie 2., *Nature methods* **9**(4): 357–359.

Langmead, B., Trapnell, C., Pop, M. and Salzberg, S. L. (2009). Ultrafast and memory-efficient alignment of short dna sequences to the human genome, *Genome Biology* **10**(3): R25.
URL: *https://doi.org/10.1186/gb-2009-10-3-r25*

Lee, M., Lee, K., Yu, N., Jang, I., Choi, I., Kim, P., Jang, Y. E., Kim, B., Kim, S., Lee, B., Kang, J. and Lee, S. (2017). Chimerdb 3.0: an enhanced database for fusion genes from cancer transcriptome and literature data mining, *Nucleic Acids Research* **45**(D1): D784–D789.
URL: *+ http://dx.doi.org/10.1093/nar/gkw1083*

Li, H. and Durbin, R. (2009). Fast and accurate short read alignment with burrows–wheeler transform, *Bioinformatics* **25**(14): 1754–1760.
URL: *+ http://dx.doi.org/10.1093/bioinformatics/btp324*

Li, H., Handsaker, B., Wysoker, A., Fennell, T., Ruan, J., Homer, N., Marth, G., Abecasis, G. and Durbin, R. (2009). The sequence alignment/map format and samtools, *Bioinformatics* **25**(16): 2078–2079.
URL: *http://dx.doi.org/10.1093/bioinformatics/btp352*

Liu, Y., Sun, J. and Zhao, M. (2017). Ongene: A literature-based database for human oncogenes, *Journal of Genetics and Genomics* **44**(2): 119 – 121.
URL: *http://www.sciencedirect.com/science/article/pii/S1673852716302053*

Lou, D. I., Hussmann, J. A., McBee, R. M., Acevedo, A., Andino, R., Press, W. H. and Sawyer, S. L. (2009). High-throughput dna sequencing errors are reduced by orders of magnitude using circle sequencing, *Proceedings of the National Academy of Sciences of the United States of America* **110**(49): 19872–19877.

Mertens, F., Johansson, B., Fioretos, T. and Mitelman, F. (2015). The emerging complexity of gene fusions in cancer, *Nat Rev Cancer* **15**(6).

Mitelman, F., Johansson, B. and Mertens, F. (2007). "the impact of translocations and gene fusions on cancer causation", *Nat Rav Cancer* **7**(4): 233 – 245.

Nervi, C., Ferrara, F. F., Fanelli, M., Rippo, M. R., Tomassini, B., Ferrucci, P. F., Ruthardt, M., Gelmetti, V., Gambacorti-Passerini, C., Diverio, D., Grignani, F., Pelicci, P. G. and Testi, R. (1998). Caspases mediate retinoic acid-induced degradation of the acute promyelocytic leukemia pml/rarα fusion protein, *Blood* **92**(7): 2244–2251.
URL: *http://www.bloodjournal.org/content/92/7/2244*

Nicorici, D., Satalan, M., Edgren, H., Kangaspeska, S., Murumagi, A., Kallioniemi, O., Virtanen, S. and Kilkku, O. (2014). Fusioncatcher - a tool for finding somatic fusion genes in paired-end rna-sequencing data, *bioRxiv* .
URL: *http://www.biorxiv.org/content/early/2014/11/19/011650*

Novo, F., de Mendíbil, I. and Vizmanos, J. (2007). Ticdb: a collection of gene-mapped translocation breakpoints in cancer., *BMC Genomics* **8**(33).

R Development Core Team (2008). *R: A Language and Environment for Statistical Computing*, R Foundation for Statistical Computing, Vienna, Austria. ISBN 3-900051-07-0.
URL: *http://www.R-project.org*

Reshmi, S. C., Harvey, R. C., Roberts, K. G., Stonerock, E., Smith, A., Jenkins, H., Chen, I.-M., Valentine, M., Liu, Y., Li, Y., Shao, Y., Easton, J., Payne-Turner, D., Gu, Z., Tran, T. H., Nguyen, J. V., Devidas, M., Dai, Y., Heerema, N. A., Carroll, A. J., Raetz, E. A., Borowitz, M. J., Wood, B. L., Angiolillo, A. L., Burke, M. J., Salzer, W. L., Zweidler-McKay, P. A., Rabin, K. R., Carroll, W. L., Zhang, J., Loh, M. L., Mullighan, C. G., Willman, C. L., Gastier-Foster, J. M. and Hunger, S. P. (2017). Targetable kinase gene fusions in high-risk b-all: a study from the children's oncology group, *Blood* **129**(25): 3352–3361.
URL: *http://www.bloodjournal.org/content/129/25/3352*

Sadedin, S. P., Pope, B. and Oshlack, A. (2012). Bpipe: a tool for running and managing bioinformatics pipelines, *Bioinformatics* **28**(11): 1525–1526.
URL: *+ http://dx.doi.org/10.1093/bioinformatics/bts167*

Serratì, S., De Summa, S., Pilato, B., Petriella, D., Lacalamita, R., Tommasi, S. and Pinto, R. (2016). Next-generation sequencing: advances and applications in cancer diagnosis., *OncoTargets and Therapy* **9**: 7355–7365.

Shugay, M., Ortiz de Mendíbil, I., Vizmanos, J. L. and Novo, F. J. (2013). Oncofuse: a computational framework for the prediction of the oncogenic potential of gene fusions, *Bioinformatics* **29**(20): 2539–2546.
URL: *+ http://dx.doi.org/10.1093/bioinformatics/btt445*

Trapnell, C., Pachter, L. and Salzberg, S. L. (2009). Tophat: discovering splice junctions with rna-seq, *Bioinformatics* **25**(9): 1105–1111.
URL: *+ http://dx.doi.org/10.1093/bioinformatics/btp120*

Wang, Y., Wu, N., Liu, J., Wu, Z. and Dong, D. (2015). Fusioncancer: A database of cancer fusion genes derived from rna-seq data, **10**: 131.

Figure

Click here to download Figure fig1.png

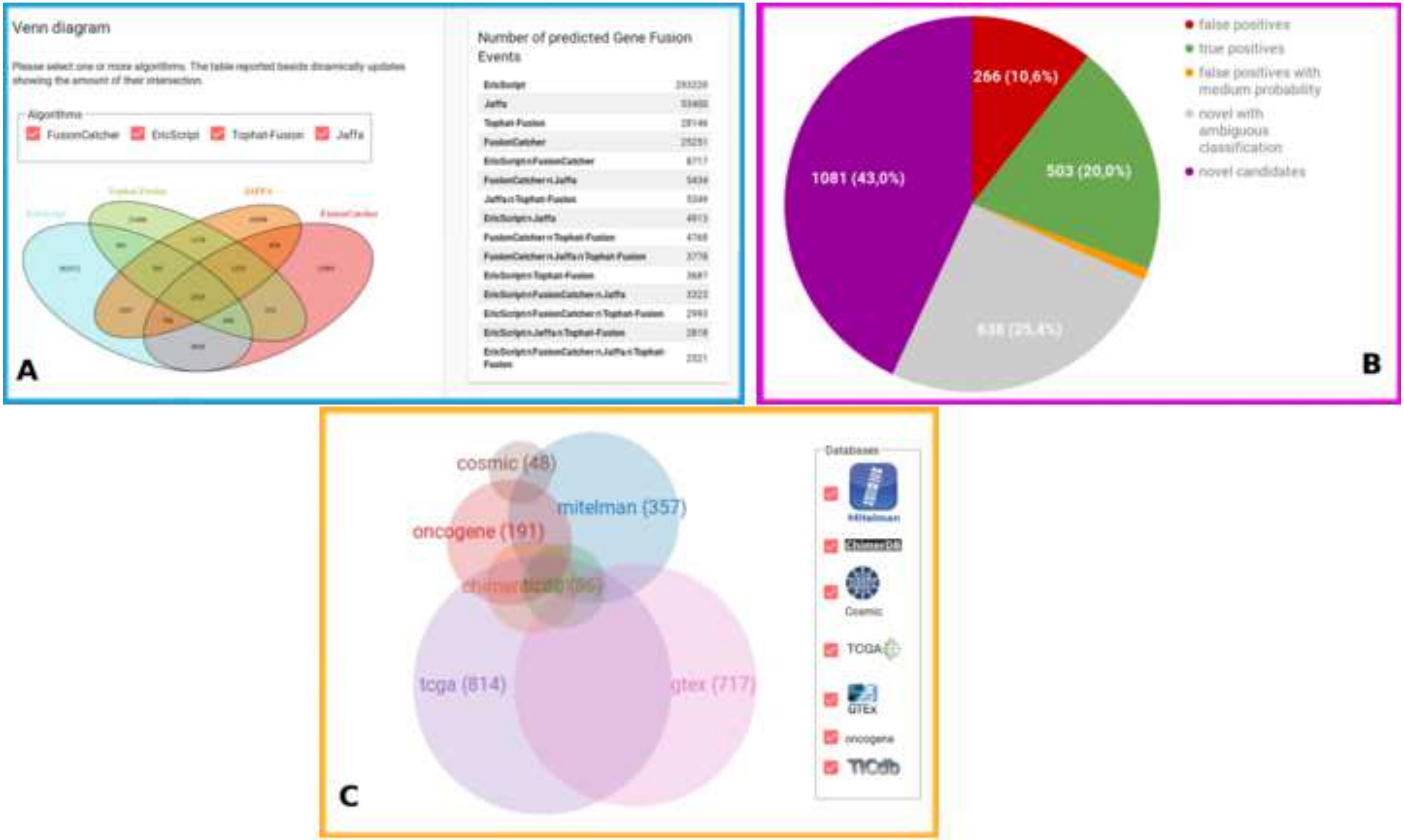

Figure

Click here to download Figure fig2.png

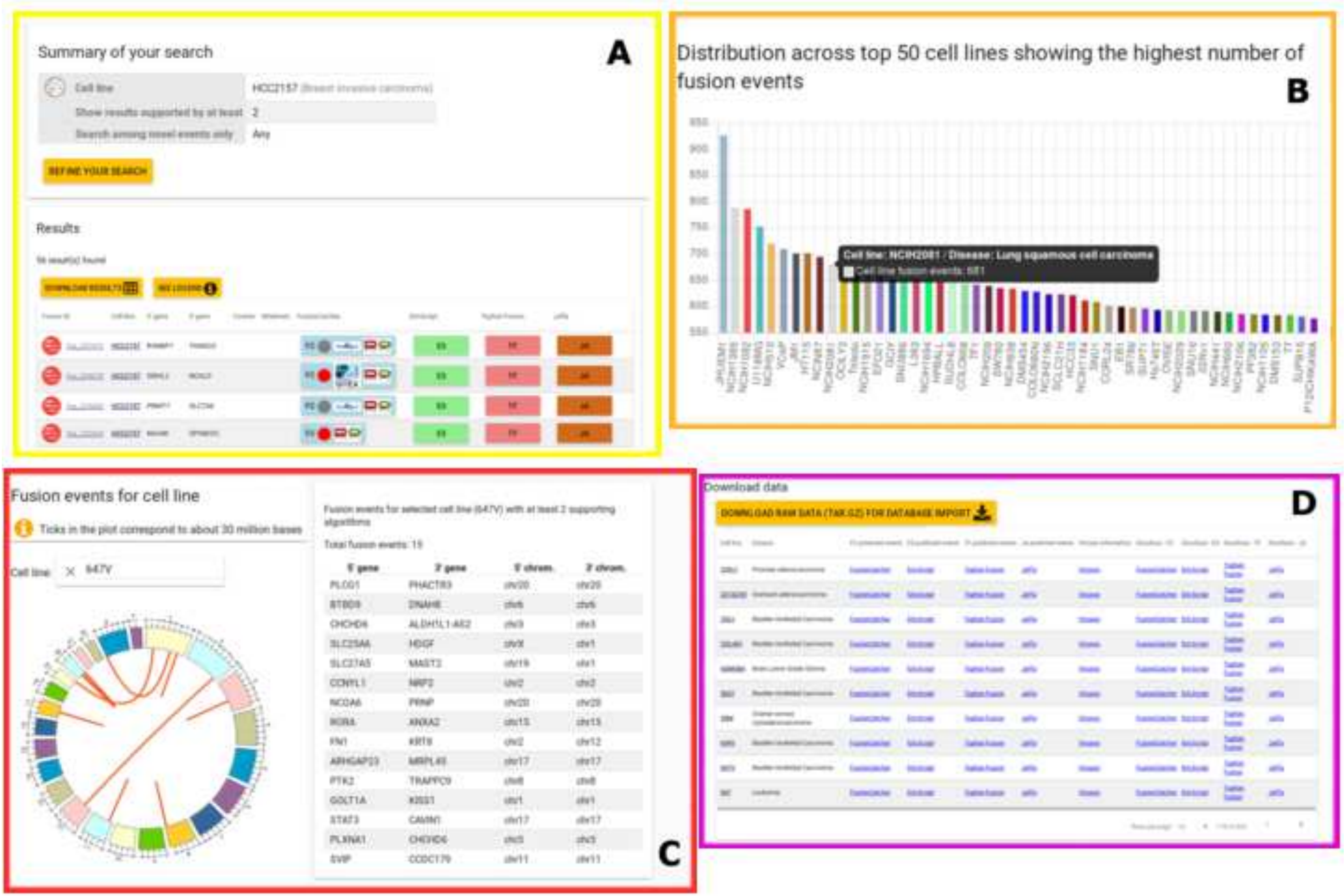

Supplement: GIGA-D-17-00241_Revision_2.pdf [file giy062_giga-d-17-00241_revision_2.pdf]
