# Supplementary material for: Massive NGS data analysis reveals hundreds of potential novel gene fusions in human cell lines
Source: Gigascience. 2018 Jun 1;7(10):giy062. doi: 10.1093/gigascience/giy062 (PMC6207142; doi:10.1093/gigascience/giy062)
Supplement: Response_to_Reviewer_Comments_Original_Submission.pdf [file giy062_response_to_reviewer_comments_original_submission.pdf]

Author's Response To Reviewer Comments

Close

Response to Editor:
Q.1.: " the reviewers point out that the web tool is extremely slow - can this be improved? This is an important point from the user's perspective. Please note the performance metrics kindly provided by reviewer 1 (attached)."
A.1: We thank the editor and the reviewers for kindly providing this feedback about the web tool performance. We do also believe that this point is very crucial from user's perspective, therefore we greatly improved the speed of the web site by leveraging both the tools and metrics suggested by the reviewer as well as including new metrics, such as PageSpeed and gtmetrix. We included all the metrics results as attachments or direct hyperlinks. We would kindly invite you to clear the browser's cache before navigating the website.
Q.2: "reviewer 1 has some useful suggestions for additional data that should be linked, e.g. genomic coordinates. I feel this would go some way in also addressing the concerns of reviewer 2 that the tool did not present a sufficient advance in its present form."
A.2: Thanks to the suggestions of the reviewer, we have now included in our portal a great variety of links, both to external resources (e.g., Gene Cards, Cancerxgene, among others) as well as to internal brand-new pages, such as those dedicated to the description of cell lines (e.g., http://hpc-bioinformatics.cineca.it/fusion/cell_line/Detroit562/) and of fusion events (e.g., http://hpc-bioinformatics.cineca.it/fusion/fusion_event/fus_49998/). Not only do these additional web pages meet the requests from reviewer 1 but, at the same time, they also foster the integrability and the interlinking across other topic-related resources.
Q.3: "reviewer 2 points out that one of the tools, EricScript, finds many more fusion genes than the other two. Why? This needs to be thoroughly addressed and explained in the manuscript."
A3: We have described in great detail the filtering process of the outputs from EricScript both in the manuscript and in the response to reviewer 2. After the filtering process, the dataset size from ES results more balanced compared to the other two softwares. Currently, LiGeA reports a set of 293,244 ES predictions which is greatly reduced from the initial size.
Q.4: "Please add licence information about any new code to your manuscript, under the "Availability and Requirements" section: (see https://academic.oup.com/gigascience/pages/instructions_to_authors#Preparing Main Manuscript Text). We usually also host an archival copy ("snapshot") of new code in our GigaDB repository. In addition, please register any new software application / database in the SciCrunch.org database to receive a RRID (Research Resource Identification Initiative ID) number, and include this in your manuscript."
A.: As requested, we have added licence information an RRID number under the "Availability and Requirements" section.

Point by point response to reviewer 1:
Major issues:
Q.1: "I've tried the database using both Chrome and Safari browsers (on several high-end laptops) and found that it is extremely slow/laggy. I mean the overall interface responsiveness. E.g. Chrome audit metrics rate the performance as 18/100 (see attachment). The 'Gene pairs statistics' shows a loading screen for around 30 seconds and then fails showing generic Chrome crash tab. It seems that the situation improves a bit after browsing the web page for a while because of caching. The web portal performance should be definitely optimized. In my humble opinion (I'm not a professional web developer), it can be improved by switching from normal Angular bindings to one-time bindings for variables that will not be updated (https://docs.angularjs.org/guide/expression, One-time

binding section)."
A.1: We have greatly improved the web portal performance. Now Chrome audit metrics rates the performance as 30/100 (instead of 18; inspect results by loading the files attached on https://googlechrome.github.io/lighthouse/viewer/). As an approximate comparison, take into account, for example, that well-known sites (such as www.cnn.com) score a performance equal to 1 (inspect the corresponding attached file). Switching to Angular one-time bindings was not a feasible option for us, since the website is built on top of a powerful, general-purpose framework we have developed in-house which is able to display arbitrary content on-the-fly (i.e., it is not possible to predict which parts will change and which will not).
In order to improve the performance of the Fusion database, we profiled the website pages and focused on improving the speed of the search pages as well as of the overall content (e.g., Download page). In order to accomplish this, we have speeded up all the queries (i.e., reducing the server-side response times) by improving and simplifying the structure of the underlying database. Also, all dropdown menus which are associated with thousands of entries (e.g., those associated with cell lines, genes or transcripts) have been converted into autocomplete fields which show only the first n matching items, thus reducing loading time significantly.
We have also fixed the problems regarding the "Gene pair statistics" page and now the page loads instantly (http://hpc-bioinformatics.cineca.it/fusion/gene_statistics).
Also the Download page has been made much faster by means of pagination: in fact, we realized that most of the times the website looked laggy because too many items were displayed (e.g., 935 rows for the Download page).
Finally, now the portal loads in half of the time (5 seconds instead of 10, according to gtmetrix - see URLs at the bottom of the answer) and appears much more responsive when browsing through the pages (total page size around 1MB).
Also, if using PageSpeed as speed metrics, the quality of the new portal has increased from 43 to 65, demonstrating the neat improvement in terms of speed and response time (please, see attachments).
We believe that everything is now fixed and we invite the reviewer to clear the browser's cache before navigating the website in order to appreciate the added changes. Still, should some page require further curation, we will make sure to fix it and/or improve it.
Q.2:"LiGeA database can benefit from providing users with a table containing a generic LiGeA fusion id and fusion genomic coordinates. These ids should be linked to other tables containing additional information on fusions: 5' and 3' genes, cell line identifier, COSMIC ids, etc. The http://hpc-bioinformatics.cineca.it/fusion/downloads/ link can be fetched via wget, yet it contains lots of intermediate processing files and no README descriptions in subfolders. This will make the life easier for bioinformaticians by allowing them to download the plain-text database version and use it for downstream analysis and annotation of RNA-Seq results without spending significant time on parsing/assembling database files."
A.2: We thank reviewer 1 for the very useful suggestions. We have assigned a generic and linkable LiGeA fusion ID to any fusion event. By clicking on it, another web page opens and the user can view additional information e.g. involved cell line and human disease, supporting algorithm(s), involved genes and genomic coordinates among the others. For example, by clicking on this link (http://hpc-bioinformatics.cineca.it/fusion/fusion_event/fus_16084/), the user can have a look at specific information regarding the gene couple PML-RARA in NB4 cell line.
Moreover, we have integrated the "Dataset" section with dedicated web pages to each Cell line identifier (e.g. http://hpc-bioinformatics.cineca.it/fusion/cell_line/CCLE_019), resuming cell line specific details, such as linkable COSMIC id, original tissue, most affected chromosomes and integrated genomes from host viruses.
Finally, as suggested by reviewer 1, we have now organized the Download page by creating a whole tar.gz file containing all the final files, thus allowing the user to download the plain-text database version. As a plus, thanks to the pagination discussed above, we give the opportunity to download

single files as well, without affecting the speed of this web page.

Minor issues:
Q.3:"The authors should compare the list of fusion events in LiGeA and previously described fusions from other datasets (those listed in Table#1, e.g. Mitelman database). Although there is some information in the Data Statistics and Validation section of the manuscript, an additional figure or table comparing LiGeA with existing databases should be added to the manuscript."
A.3: "There is an interactive panel on LiGeA portal, under the Database Statistics menu, named "Intersection of our database with existing databases". The interactive Venn Diagram shows how many pGFEs (predicted Gene Fusion Events) in the CCS (Consensus call set) shown under LiGeA portal, are already present in other databases listed in Table#1 (e.g. chimerdb2, Cosmic, TCGA and so on). As suggested, we have added this figure to the manuscript (Fig. 1C).

Q.4: "The authors should comment on their specific choice of the fusion calling algorithms. Perhaps including additional fusion detection software such as STAR can yield more fusions/increase the confidence of existing fusion calls?"
The choice of the algorithms was driven by the paper from Kumar S. et al., (Nature, 2016), which compared twelve methods for the fusion transcripts detection from RNA-Seq data. In this paper, for each tool TOPSIS (Technique for Order of Preference by Similarity to Ideal Solution) scores were calculated by weighting four criteria i.e. sensitivity, time consumption (minutes), computational memory (RAM), and PPV (Positive Predictive Value). EricScript and FusionCatcher gained the best TOPSIS scores (0.93 and 0.87, respectively), followed by Bellerophontes and FusionMap (0.84 for both). Unfortunately, the latter two softwares presented a bug when trying to annotate gene fusions on hg38 human genome version. Therefore, we chose the Tophat-Fusion algorithm which was ranked immediately after (0.74). We have now added a more detailed explanation of the reasons leading to the specific choices of these tools, under the "Methods" section of the manuscript. As suggested by reviewer 1, we had a look at STAR but, to our knowledge, STAR is a read aligner and, moreover, FusionCatcher already relies on Bowtie, Blat, and STAR aligners to predict gene fusions. On the other hand, STAR-Fusion is a novel algorithm for fusion detection for which only a preprint abstract version of the paper is available at the moment (https://www.biorxiv.org/content/early/2017/03/24/120295). We would rather prefer to take advantage of peer-reviewed algorithms but we do not exclude to integrate results from other softwares in the future versions of the database.

Point by point response to reviewer2:
Q.1: "First, one of software is giving *too many* fusion events compared with the other two. It means either one of software is giving incorrect results. Whatever the results are, if they are giving more than 10 times bigger results than other software, it means it is not acceptable. There are a tons of software for this purpose - finding fusion genes. Authors need to be very careful when choosing some of them because detecting fusion events from RNA-Seq require very sophisticated optimization and filtering process as well as long calculation time. Authors need to do additional filtering steps for results from EricScript. Otherwise users will suspect something wrong with the final dataset."
A.1: We thank reviewer 2 for pointing out this very important issue.
It is true that tons of softwares exist for the purpose of finding fusion genes, but the choice of EricScript was driven by this very useful assessment (Kumar S. et al., Nature, 2015), which compared twelve methods for the fusion transcripts detection from RNA-Seq data indicating EricScript as the most performing one both in terms of sensibility and Positive Predictive Value.
Anyway, we agree with reviewer 2 that EricScript final results were too many compared to the other two. Therefore, as suggested, we did additional filtering steps for results from EricScript (initial dataset size: 929,638 predicted Gene Fusion Events - pGFEs).

First of all, we removed all the predictions for which the software was not able to predict an exact breakpoint position because such pGFEs could not even be experimentally validated (# of events passing the filter: 748,066).
Secondly, as already applied to FusionCatcher and Tophat Fusion results, we retained the pGFEs exhibiting at least 3 spanning reads over the gene fusion junction (# of events passing the filter: 486,174).
Furthermore, we filtered out all the pGFEs with Ericscore value less than 0.85. EricScore is a ranking parameter ranging from 0.5 to 1: greater values correspond to better predictions (# of events passing the filter: 293,244). Interestingly, by applying these filters, we filtered out almost 2/3 of the predictions from EricScript but, at the same time, the Consensus CallSet did not reduce substantially (from 3,294 to 2,926), thus indicating that the choice of a Consensus of predictions is a good strategy to obtain a reliable set of gene fusion candidates to be experimentally validated (please, see attached figure - also provided as supplementary material to the article). Currently, LiGeA reports a set of 293,244 ES predictions which is greatly reduced from the initial size. We are aware that this is still a high number as compared to the results of the other two algorithms and is probably bound to contain a higher number of false positives. However, the purpose of the present database is to provide scientists with a broad overview of the possible gene-fusion events in cancer cell lines. Depending on its interests, each user will be able to decide whether to look for high-confidence, well established, already described fusions, or for low-confidence but potentially interesting and novel events. In the latter case, a further experimental validation will be obviously needed.

Q.2:"Second, p.4 line 18. Data Statistics and Validation section. Instead of overall statistics, 95% overlap with previously known cancer gene, please give how exactly it can detect experimentally validated fusion events from individual cell lines."
A.2: We thank reviewer 2 for this suggestion and we believe that this sentence was misunderstood and needed to be reformulated in the manuscript as follows: "As a validation of our analysis, 644 out of the 699 (92%) genes known to be functionally implicated in cancer and collected under COSMIC gene census (http://cancer.sanger.ac.uk/census), are present in our final dataset."
Furthermore, we give information about how exactly we can detect cell line-specific experimentally validated fusion events thanks to the colorful signature shown in LiGeA portal under the "Search for" tabs. Indeed, whenever a gene fusion couple has been already described as true positive whether in the literature or in other databases, a green circle is added to the gene fusion event. In this way, the user can choose events not tagged with the green circle and be addressed to further study the novel predictions only.

Q.3: "Finally, web-server is just showing calculation results from three software. If one browses that database, he/she can only get information which software is giving this results. But no novel intuitions or dataming from each content.
Thank you for our huge work, but readers need at least one scientific intuition or improvement from them. And, the database is very slow due to heavy use of javascript (I don't know exactly what WWW techniques are used). I think the database itself is not that big, and it could be improved. And, the database is very slow due to heavy use of javascript (I don't know exactly what WWW techniques are used). I think the database itself is not that big, and it could be improved."
A.3: Indeed, we feel that the LiGeA database Portal is not only a mere collection of calculation results. Its primary aim is indicating which putative fusion gene events could be experimentally validated and studied. About half of the Consensus Call Set is represented by fusion genes not yet described neither in the literature nor in other dedicated databases, therefore we believe that LiGeA could become a handy resource for many wet lab biologists who take advantage of cell lines in order to study human malignancies and oncogenic gene fusions. In addition, LiGeA is integrated with other useful external resources, thus allowing the extraction of further biologically meaningful information. For example:

Whenever the gene fusion couple has already been experimentally validated, an external link to COSMIC database (http://cancer.sanger.ac.uk/cosmic, a catalog of somatic mutations in cancer) is shown;
Whether one of the two genes involved in the fusion event has been already described to be causally implicated in cancer, an external link to the Cancer Gene Census is provided (http://cancer.sanger.ac.uk/census);
A colourful signature has been added to tag the FusionCatcher predictions as 'validated truly positive couple' (green circle), 'validated false positive couple' (red circle) and 'false positive couple with medium probability' (orange circles).
A functional prediction score (oncogenic potential, i.e. the probability of being 'driver' events in carcinogenesis) obtained by extensively running the Oncofuse software (http://www.unav.es/genetica/oncofuse.html), is reported as additional tag to each of the three kinds of results.
We agree that downstream analysis on this huge amount of data could hint further biological intuitions and it is for this reason that we have increased the data accessibility and fostered the easiness of data download by providing access also to the plain-text database version under the Download page and thus encouraging users to re-use our data.
Moreover, we would like to underline that the main focus of Giga Science is promoting reproducibility of analyses and big data dissemination, organization, understanding, and use. In particular, Data Notes "highlights and helps to contextualize exceptional datasets to encourage reuse… Data Notes focus on a particular dataset, and provide detailed methodology on data production, validation, and potential reuse. Supporting the FAIR Principles for scientific data management and stewardship that state that research data should be Findable, Accessible, Interoperable and Reusable."
As regards the overall website speed, we have dramatically improved the loading and response time of the portal. Now the site loads very quickly (around 1 second) and, after improving the structure of the database and applying small fixes (e.g., pagination in the Download page, autocomplete fields in the search pages), browsing turned out to be much easier. We invite the reviewer to clear the browser's cache before navigating the website in order to appreciate the added changes.

Point by point response to reviewer 3:
Q.1:"On page 1, line 12, the "gene fusion events result from chromosomal rearrangements" should be changed to "oncogenic gene fusion events result from chromosomal rearrangements" because fusion genes occur also in healthy organisms. Fusion of genes is also one of the evolutionary mechanism for creating a new gene in a healthy organism. Not all fusion genes are oncogenic. For example, there are plenty of fusion genes known to exist in healthy people, like for example TTTY15-USP9Y, SLC45A3-ELK4, MSMB-NCOA4."
A.: We changed the sentence as suggested.

Q.2: "On page 1, lines 37-40, the text "Moreover, each gene fusion predictions differes...chromosomal rearrangements (Mertens et al.; 2015)" should be removed from the article because it is not correct. This is not correct because some fusion gene finder can call very well a certain type of fusion genes whilst all the other fusion caller will miss the and therefore the consensus here is not the best. For example, FusionCatcher is the only fusion finder which is able to call IGH fusions (see: https://doi.org/10.1182/blood-2016-12-758979)."
A.: We thank reviewer 3 for this suggestion and we agree that FusionCatcher is the only fusion finder able to call IGH fusions (named IGH@ by FusionCatcher annotation). Indeed, since we also believe that the consensus method is not necessarily the best choice, we give the opportunity to navigate the results from individual algorithms and by means of the dedicated "search by algorithm" function. Therefore we removed the sentence on page 1, lines 37-40 and we have added the reference cited by the reviewer to the article (https://doi.org/10.1182/blood-2016-12-758979).

Nevertheless, many other known gene fusion couples reported in literature (e.g. FGFR3-TACC3, PML-RARA, BCR-ABL1 just to cite some) have been correctly identified by all the softwares we used. Since the aim of LiGeA portal is addressing researchers to study and validate potential novel cell line-specific gene fusion events, we believe that researchers could benefit as well from choosing fusion candidates predicted from more than one algorithm. Choosing more "reliable" targets might help them in saving time and resources, speeding up the process of experimental validation and this is why we have built the Consensus Call Set. Anyway, we don't want to claim that one method is better than the other, since we believe that it is up to the wet lab biologists to choose the way they prefer to select the targets to validate and to study.

Q.3: "When searching for a gene using the http://hpc-bioinformatics.cineca.it/fusion/search_for_gene is very slow. This should be fixed. Also it is very important to list and show the fusion genes which are supported only by one fusion finder. For example the fusion DUX4-IGH is known to exist in NALM6 cell line which is one of the 935 cell lines but when looking for it in LiGeA database, it does not show up because the TOPHAT-fusion and EricScript are not able to find fusions which involve DUX4 gene or IGH gene."
A.3: We thank reviewer 3 for suggesting to improve the "search for gene" function and now it is definitely faster. We invite the reviewer to clear the browser's cache before navigating the website in order to appreciate the added changes.
Furthermore, we would like to point out that it is already possible to list and show the fusion genes supported only by one fusion finder. Whenever the user submits a query, it is possible to select the outputs from one, two or three algorithms by checking the "show results supported by at least n algorithms" menu. Even if the user does not use this facility, the results are simply paginated and sorted by those supported by more algorithms followed by those supported only by one.
Eventually, it is also possible to query the Database by the "Search by algorithm" function in order to choose one's own algorithm of election.
