# Supplementary material for: Massive NGS data analysis reveals hundreds of potential novel gene fusions in human cell lines
Source: Gigascience. 2018 Jun 1;7(10):giy062. doi: 10.1093/gigascience/giy062 (PMC6207142; doi:10.1093/gigascience/giy062)
Supplement: Response_to_Reviewer_Comments_Revision_1.pdf [file giy062_response_to_reviewer_comments_revision_1.pdf]

Author's Response To Reviewer Comments

Close

Dear Editor,
we have taken in great consideration the points raised during the process of revision and we have added a fourth algorithm and satisfied the requests from the reviewers. We would kindly invite you and the reviewers to clear the browser's cache before navigating the website in order to appreciate the changes.

#####################Point by point answers to Reviewer#1:

RE A.1: I've checked new version of the web portal and confirm that the performance has improved substantially.
Answer RE A.1: Thank you.

RE A.2: Most recently published databases provide an option to download the full database as a single table. This is what was originally meant. Right now the authors provide the option to download raw results of Tophat, EricScript and FusionCatcher software, each in its own format, as a single gzipped archive with subfolders. While its helpful, it is not directly related to the database (otherwise it means that the database is just a collection of computational results without any systematization). It is also not that convenient for bioinformaticians, as stressed in my original question. So the authors should provide the users the ability to download the compiled database as a single plain-text table for the downstream analysis.
Answer RE A.2: The whole portal leverages on graph-based technologies (i.e., Neo4j, in our case) for resolving and executing all the queries raised by the portal's user. The database is not just a collection of computational results: the original raw data (given by the four algorithms along with a substantial amount of annotations and enrichment data) has been parsed, extracted and converted into a format suitable for graph-based data storage and representation. On the one hand, the most technical one probably, the systematization has consisted in the extraction of information and the subsequent formalization into a set of textual files representing the nodes and the relationships of the graph database, but on the other hand it implied parsing, filtering and analyzing the data in order to give it a sense and a value from a biological point of view (e.g., deciding to discard chromosome M or establishing interlinking with external resources, etc.).
In our previous release, the reason why we decided to make all the raw-data available for download was to allow the user to re-shape the same data into a different form, thus enabling a potential reuse of data (e.g., storage into SQL-like database, simple command-line post-processing or even further filtering and checking).
As regards the possibility to have a single plain-text file to import (as it was possible with SQL-like database), this is not possible anymore, to our knowledge. Despite this, Neo4j provides a tool for importing an existing database, starting from a set of plain-text CSV files. To this end, we now provide a tar.gz with i) a directory containing all the CSV files containing the systematized information and ii) a script for importing this data into Neo4j (this assumes you have Neo4j installed somewhere in your file system and only requires the Neo4j base directory).

RE A.4:
"> The choice of the algorithms was driven by the paper from Kumar S. et al., (Nature, 2016), which compared twelve methods for the fusion transcripts detection from RNA- Seq data. In this paper, for each tool TOPSIS (Technique for Order of Preference by Similarity to Ideal Solution) scores were calculated by weighting four criteria i.e. sensitivity, time consumption (minutes), computational

memory (RAM), and PPV (Positive Predictive Value). EricScript and FusionCatcher gained the best TOPSIS scores (0.93 and 0.87, respectively), followed by Bellerophontes and FusionMap (0.84 for both)."

I don't see how time/RAM consumption are relevant for selection of a software to pre-compute fusions for the database.

"> On the other hand, STAR-Fusion is a novel algorithm for fusion detection for which only a preprint abstract version of the paper is available at the moment (https://www.biorxiv.org/content/early/2017/03/24/120295). We would rather prefer to take advantage of peer-reviewed algorithms but we do not exclude to integrate results from other softwares in the future versions of the database."

As stated in the original paper, "STAR can discover non-canonical splices and chimeric (fusion) transcripts, and is also capable of mapping full-length RNA sequences." So the fusion detection was implemented in it from the beginning and STAR-Fusion is an extension/update of the method originally published in a peer-reviewed paper.

"> The choice of the algorithms was driven by the paper from Kumar S. et al., (Nature, 2016), which compared twelve methods for the fusion transcripts detection from RNA- Seq data. In this paper, for each tool TOPSIS (Technique for Order of Preference by Similarity to Ideal Solution) scores were calculated by weighting four criteria i.e. sensitivity, time consumption (minutes), computational memory (RAM), and PPV (Positive Predictive Value). EricScript and FusionCatcher gained the best TOPSIS scores (0.93 and 0.87, respectively), followed by Bellerophontes and FusionMap (0.84 for both). Unfortunately, the latter two softwares presented a bug when trying to annotate gene fusions on hg38 human genome version. Therefore, we chose the Tophat-Fusion algorithm which was ranked immediately after (0.74)."

Unfortunately, I find the arguments used by authors for not including results from other software tools not very convincing.

I still believe that authors should include results of additional software tools, at least for the following reasons:

1) It will demonstrate that the database can be updated in a reasonable time and will be kept up-to-date. If it is problematic to include additional software tools in the database at this stage (e.g. due to database architecture limitations), I doubt that the future database updates will be feasible.

2) Current software choice is based on just a single paper describing software comparison. Another study by Liu et al NAR 2015 (https://www.ncbi.nlm.nih.gov/pmc/articles/PMC4797269/pdf/gkv1234.pdf) gives a different ranking: SOAPfuse, FusionCatcher, JAFFA, EricScript, ... with TopHat-Fusion ranked 9th.

3) Figure 1A shows that there is little convergence/agreement between software tools. It is also not clear what choice of the software tool combination will yield optimal database search results, so the users should be provided with as many options to perform the filtering as possible.

Answer RE A.4.: We understand the reviewer's point of view when he asserts that time and RAM consumption are not strictly relevant for the selection of the softwares to use; indeed these two criteria had been previously solely considered in order to evaluate the feasibility of the tools for this big data analysis. On the other hand, the Positive Prediction Values and sensitivity had had a greater weight on the choice of the softwares.

Nevertheless, we would like to point out that Kumar et al., in the paper published on Nature in 2016, also underline that: "On the basis of Positive Prediction Values (PPV), the tools can be ordered as follows: EricScript (100%) = FusionCatcher (100%) = TopHat-Fusion (100%) > JAFFA (95.6%)". Since the main aim of LiGEA is to address researchers to validate high-confidence in-silico predicted gene fusions, we think that the Positive Predictive Value is the best fitting criterion for the choice of the algorithms to use and we are glad to see that ES, FC and TF have an excellent PPV. Therefore, we added this further argumentation to the manuscript.

We thank the reviewer for bringing to our attention the assessment performed by Liu at al. (NAR, 2015).
Since JAFFA is ranked at the 2nd position in the assessment by Kumar et al. (Nature, 2016) and, at the same time, it is also ranked 3rd in the paper by Liu et al. (NAR 2015) we have decided to integrate this fourth software to analyze, construct and systematize data into our LiGeA portal.
The detailed new statistics and Consensus CallSet have been reported in the revised version of the manuscript. Overall, the inclusion of a fourth software has brought to a very small reduction of the Consensus Call (~ 11%) passing from 2,828 to 2,521 extremely highly confident predictions. Not only this is a very good result indicating that the CCS is very robust, but it also provides a key for navigating the database, suggesting that also those predictions supported by at least three methods have a very good level of confidence.
We would like to underline that STAR is not assessed neither in Kumar's either in Liu's assessment, therefore making it difficult to justify its inclusion in our methodology. Notably, FusionCatcher relies on three aligners, including STAR, to increase the accuracy of alignment and fusion breakpoint detection. This means that the choice of STAR as the fourth software would have probably added several redundant results to our analysis, thus losing the important goal underlined both by the editor and the reviewer about increasing the accuracy of the Consensus Call Set.
As regards STAR-Fusion, we downloaded and used the pre-built indexes provided with the software and we tried to run it on a sub-sample of our dataset. Unfortunately, while STAR-fusion correctly ran and produced correct outputs when performed on the test files provided by the authors of the software, the output files were completely empty when running STAR-fusion on the cell lines' fastq files. It seems this is not the first time it happens, according to other open issues we found on forums dedicated to STAR-Fusion, probably due to a problem related to plug and play version of the genome resource lib released by the authors (https://github.com/STAR-Fusion/STAR-Fusion/issues/2).
Unfortunately, at the state of the art, STAR-Fusion does not seem to be very robust, therefore making it impossible for us to use since, also considering the importance of the topic, we want to provide highly reliable predictions.
It took less than two months, most of which spent running the algorithm, to seamlessly integrate the results from a fourth software, thus demonstrating that future updates of the database would not be problematic at all. We will thus be glad to integrate the results from STAR-Fusion as soon as it will be published and released in a more stable version.

####################Point by point answers to Reviewer#2:
Major Points:
1. First, in "Search by" tab, when I tried to input ALK gene in 3' partner, it is taking some time to load gene list and then select it. People usually get user's input and then search their database to return the results. Please consider what would be better to get user input.
A1. We have slightly modified the behaviour of our search pages. Now all the autocomplete fields accept any text which has been typed in (regardless of the fact that the gene list has been already loaded or not). Also, as before, they also keep listing the matching candidates known to the system.

2. In Statistics page, please provide with color legends on colorful pie chart. (now it gives mouse-over legends).
A2. The legend has been changed as suggested.

Minor points:
3. All the gene symbols are upper characters. for example, C9ORF66 gene, it is actually C9orf66 according to HGNC. I think authors did for their conveniences, but I think the gene symbols should follow HGNC nomenclature. Please compare and update your gene symbol according to latest

version of HGNC and their aliases. It will be better to give official gene symbol and aliases. For example, official symbol of well-known HER2 gene is ERBB2. People tends to assume that all gene symbols are upper characters, but NOT.
A3. Thank you for pointing out this important issue. Now the gene symbols follow the latest HGNC nomenclature.

4. In each link, please give "EXACT" links rather than main page. for example, I noticed that a link for ALK gene is http://cancer.sanger.ac.uk/cosmic/census but it SHOULD be pointing to http://cancer.sanger.ac.uk/cosmic/gene/analysis?ln=ALK as well as genecards http://www.genecards.org/cgi-bin/carddisp.pl?gene=ALK&keywords=ALK
Please find all links and give their EXACT url links, NOT top page.
A4.: The gene ALK points to two different pages under COSMIC because ALK is a Cosmic Gene and, at the same time, it also belongs to the subset of the 719 genes encompassed in Cosmic Gene Census list. Therefore, this link http://cancer.sanger.ac.uk/cosmic/gene/analysis?ln=ALK brings to the specific web page dedicated to ALK, while this one http://cancer.sanger.ac.uk/cosmic/census points to the page containing the table with all the Census genes (this is not a top page). Nevertheless, we noticed that this information was not described very clearly in the manuscript, therefore we rewrote some sentences in the paragraph dedicated to Cosmic Gene Census. Furthermore, we downloaded and updated the newly released list (from 699 genes during the previous round of revision to 719 genes at the current date) and computed the new statistics.

5. In fusion gene info, you used NPM1#ALK but in each info page, you used NPM1/ALK. Please use same separator.
A5. Thank you for pointing out this discrepancy. Now we use always the same separator.

6. In each search page, can you delete mouse-over message "this fusion event is supported by...." I don't think this long message is giving valuable information. It is already under "FusionCatcher", "EricScript" and "Tophat-fusion". And I noticed that each icons are linked to same page, not even pubmed icon. I thought pubmed Icon can lead me to pubmed but NOT.
A6: As suggested, we removed the mouse-over message:"this fusion event is supported by...". As regards the icons, they are a graphical representation of the systematization of the results, this is explained in the legend posed upon the table of results. On the other hand, each predicted gene fusion event has a unique ID, pointing to a dedicated webpage where these icons are linked to the respective external resources.

7. Please give thorough examination on each page again, menu, and etc. I thought that you have answered all questions but I can still found many errors.
A7: We had another round of examination over each page. Thank you.

########################Answer to Reviewer#3:
An useful article and database of fusion genes in human cancer cell lines for research community.
A: Thank you.

Close
