# Supplementary material for: Massive NGS data analysis reveals hundreds of potential novel gene fusions in human cell lines
Source: Gigascience. 2018 Jun 1;7(10):giy062. doi: 10.1093/gigascience/giy062 (PMC6207142; doi:10.1093/gigascience/giy062)
Supplement: Response_to_Reviewer_Comments_Revision_2.pdf [file giy062_response_to_reviewer_comments_revision_2.pdf]

Author's Response To Reviewer Comments

Close

Dear Editor,

as requested in point (1), a reference to our GigaDB dataset has been included in the bibliography with the number 33.

We also made the necessary corrections requested in point (2) and uploaded a revised manuscript.

Best regards,
Tiziana Castrignanò

Close
