# Supplementary material for: Massive NGS data analysis reveals hundreds of potential novel gene fusions in human cell lines
Source: Gigascience. 2018 Jun 1;7(10):giy062. doi: 10.1093/gigascience/giy062 (PMC6207142; doi:10.1093/gigascience/giy062)
Supplement: Reviewer_1_Report_(Original_Submission) -- Mikhail Shugay — 9/29/2017 Reviewed [file giy062_reviewer_1_report_(original_submission).pdf]

Reviewer Report

Title: Massive NGS Data Analysis Reveals Hundreds Of Potential Novel Gene Fusions in Human Cell Lines

Version: Original Submission **Date:** 9/29/2017

Reviewer name: Mikhail Shugay

Reviewer Comments to Author:

The authors describe a database of high-confidence gene fusions events obtained from The Cancer Cell Line Encyclopedia using three different fusion detection algorithms. The fusions are then extensively annotated and can be browsed using a dedicated web-based interface hosted at http://hpc-bioinformatics.cineca.it/fusion/. Overall, I think the LiGeA database is an extremely useful resource that provides a valuable reference point for biologists and bioinformaticians studying oncogenic gene fusions. There is still a couple of critical issues the that should be addressed:

Major issues:

I've tried the database using both Chrome and Safari browsers (on several high-end laptops) and found that it is extremely slow/laggy. I mean the overall interface responsiveness. E.g. Chrome audit metrics rate the performance as 18/100 (see attachment). The 'Gene pairs statistics' shows a loading screen for around 30 seconds and then fails showing generic Chrome crash tab. It seems that the situation improves a bit after browsing the web page for a while because of caching. The web portal performance should be definitely optimized. In my humble opinion (I'm not a professional web developer), it can be improved by switching from normal Angular bindings to one-time bindings for variables that will not be updated (https://docs.angularjs.org/guide/expression, One-time binding section).

LiGeA database can benefit from providing users with a table containing a generic LiGeA fusion id and fusion genomic coordinates. These ids should be linked to other tables containing additional information on fusions: 5' and 3' genes, cell line identifier, COSMIC ids, etc. The http://hpc-bioinformatics.cineca.it/fusion/downloads/ link can be fetched via wget, yet it contains lots of intermediate processing files and no README descriptions in subfolders. This will make the life easier for bioinformaticians by allowing them to download the plain-text database version and use it for downstream analysis and annotation of RNA-Seq results without spending significant time on parsing/assembling database files.

Minor issues:

The authors should compare the list of fusion events in LiGeA and previously described fusions from other datasets (those listed in Table#1, e.g. Mitelman database). Although there is some information in the Data Statistics and Validation section of the manuscript, an additional figure or table comparing LiGeA with existing databases should be added to the manuscript.

The authors should comment on their specific choice of the fusion calling algorithms. Perhaps including additional fusion detection software such as STAR can yield more fusions/increase the confidence of existing fusion calls?

Level of Interest

Please indicate how interesting you found the manuscript: An article of importance in its field

Quality of Written English

Please indicate the quality of language in the manuscript: Needs some language corrections before being published

Declaration of Competing Interests

Please complete a declaration of competing interests, considering the following questions:

- Have you in the past five years received reimbursements, fees, funding, or salary from an organisation that may in any way gain or lose financially from the publication of this manuscript, either now or in the future?
- Do you hold any stocks or shares in an organisation that may in any way gain or lose financially from the publication of this manuscript, either now or in the future?
- Do you hold or are you currently applying for any patents relating to the content of the manuscript?
- Have you received reimbursements, fees, funding, or salary from an organization that holds or has applied for patents relating to the content of the manuscript?
- Do you have any other financial competing interests?
- Do you have any non-financial competing interests in relation to this paper?

If you can answer no to all of the above, write 'I declare that I have no competing interests' below. If your reply is yes to any, please give details below.

I declare that I have no competing interests

I agree to the open peer review policy of the journal. I understand that my name will be included on my report to the authors and, if the manuscript is accepted for publication, my named report including any attachments I upload will be posted on the website along with the authors' responses. I agree for my report to be made available under an Open Access Creative Commons CC-BY license (http://creativecommons.org/licenses/by/4.0/). I understand that any comments which I do not wish to be included in my named report can be included as confidential comments to the editors, which will not be published.

I agree to the open peer review policy of the journal

To further support our reviewers, we have joined with Publons, where you can gain additional credit to further highlight your hard work (see: https://publons.com/journal/530/gigascience). On publication of this paper, your review will be automatically added to Publons, you can then choose whether or not to claim your Publons credit. I understand this statement.

Yes
