# Supplementary material for: Massive NGS data analysis reveals hundreds of potential novel gene fusions in human cell lines
Source: Gigascience. 2018 Jun 1;7(10):giy062. doi: 10.1093/gigascience/giy062 (PMC6207142; doi:10.1093/gigascience/giy062)
Supplement: Reviewer_1_Report_(Revision_1) -- Mikhail Shugay — 1/15/2018 Reviewed [file giy062_reviewer_1_report_(revision_1).pdf]

Reviewer Report

Title: Massive NGS Data Analysis Reveals Hundreds Of Potential Novel Gene Fusions in Human Cell Lines

Version: Revision 1 **Date:** 1/15/2018

Reviewer name: Mikhail Shugay

Reviewer Comments to Author:

I'm overall satisfied with the work the authors performed to improve the web portal look and performance. However, I think that the authors have not fully addressed some other important points, see below.RE A.1:I've checked new version of the web portal and confirm that the performance has improved substantially.RE A.2:Most recently published databases provide an option to download the full database as a single table. This is what was originally meant. Right now the authors provide the option to download raw results of Tophat, EricScript and FusionCatcher software, each in its own format, as a single gzipped archive with subfolders. While its helpful, it is not directly related to the database (otherwise it means that the database is just a collection of computational results without any systematization). It is also not that convenient for bioinformaticians, as stressed in my original question. So the authors should provide the users the ability to download the compiled database as a single plain-text table for the downstream analysis.RE A.4:> The choice of the algorithms was driven by the paper from Kumar S. et al., (Nature, 2016), which compared twelve methods for the fusion transcripts detection from RNA- Seq data. In this paper, for each tool TOPSIS (Technique for Order of Preference by Similarity to Ideal Solution) scores were calculated by weighting four criteria i.e. sensitivity, time consumption (minutes), computational memory (RAM), and PPV (Positive Predictive Value). EricScript and FusionCatcher gained the best TOPSIS scores (0.93 and 0.87, respectively), followed by Bellerophontes and FusionMap (0.84 for both).I don't see how time/RAM consumption are relevant for selection of a software to pre-compute fusions for the database.> On the other hand, STAR-Fusion is a novel algorithm for fusion detection for which only a preprint abstract version of the paper is available at the moment (https://www.biorxiv.org/content/early/2017/03/24/120295). We would rather prefer to take advantage of peer-reviewed algorithms but we do not exclude to integrate results from other softwares in the future versions of the database.As stated in the original paper, "STAR can discover non-canonical splices and chimeric (fusion) transcripts, and is also capable of mapping full-length RNA sequences." So the fusion detection was implemented in it from the beginning and STAR-Fusion is an extension/update of the method originally published in a peer-reviewed paper.> The choice of the algorithms was driven by the paper from Kumar S. et al., (Nature, 2016), which compared twelve methods for the fusion transcripts detection from RNA- Seq data. In this paper, for each tool TOPSIS (Technique for Order of Preference by Similarity to Ideal Solution) scores were calculated by weighting four criteria i.e. sensitivity, time consumption (minutes), computational memory (RAM), and PPV (Positive Predictive Value). EricScript and FusionCatcher gained the best TOPSIS scores (0.93 and 0.87, respectively), followed by Bellerophontes and FusionMap (0.84 for both). Unfortunately, the latter two softwares presented a bug when trying to annotate gene fusions on hg38 human genome version. Therefore, we chose the Tophat-Fusion algorithm which was ranked immediately after (0.74).Unfortunately, I find the arguments used by authors for not including results from other software tools not very convincing. I still believe that authors should include results of additional software tools, at least for the following reasons:1) It will demonstrate that the database can be updated in a reasonable time and will be kept up-to-date. If it is problematic to include additional software tools in the database at this stage (e.g. due to database architecture limitations), I doubt that the future database updates will be feasible.2) Current software choice is based on just a single paper describing software comparison. Another study by Liu et al NAR 2015 (https://www.ncbi.nlm.nih.gov/pmc/articles/PMC4797269/pdf/gkv1234.pdf) gives a different ranking: SOAPfuse, FusionCatcher, JAFFA, EricScript, ... with TopHat-Fusion ranked 9th.3) Figure 1A shows that there is little convergence/agreement between software tools. It is also not clear what choice of the software tool

combination will yield optimal database search results, so the users should be provided with as many options to perform the filtering as possible.

Level of Interest

Please indicate how interesting you found the manuscript: An article of importance in its field

Quality of Written English

Please indicate the quality of language in the manuscript: Acceptable

Declaration of Competing Interests

Please complete a declaration of competing interests, considering the following questions:

- Have you in the past five years received reimbursements, fees, funding, or salary from an organisation that may in any way gain or lose financially from the publication of this manuscript, either now or in the future?
- Do you hold any stocks or shares in an organisation that may in any way gain or lose financially from the publication of this manuscript, either now or in the future?
- Do you hold or are you currently applying for any patents relating to the content of the manuscript?
- Have you received reimbursements, fees, funding, or salary from an organization that holds or has applied for patents relating to the content of the manuscript?
- Do you have any other financial competing interests?
- Do you have any non-financial competing interests in relation to this paper?

If you can answer no to all of the above, write 'I declare that I have no competing interests' below. If your reply is yes to any, please give details below.

I declare that I have no competing interests

I agree to the open peer review policy of the journal. I understand that my name will be included on my report to the authors and, if the manuscript is accepted for publication, my named report including any attachments I upload will be posted on the website along with the authors' responses. I agree for my report to be made available under an Open Access Creative Commons CC-BY license (http://creativecommons.org/licenses/by/4.0/). I understand that any comments which I do not wish to be included in my named report can be included as confidential comments to the editors, which will not be published.

I agree to the open peer review policy of the journal

To further support our reviewers, we have joined with Publons, where you can gain additional credit to further highlight your hard work (see: https://publons.com/journal/530/gigascience). On publication of this paper, your review will be automatically added to Publons, you can then choose whether or not to claim your Publons credit. I understand this statement.

Yes
