# Supplementary material for: Massive NGS data analysis reveals hundreds of potential novel gene fusions in human cell lines
Source: Gigascience. 2018 Jun 1;7(10):giy062. doi: 10.1093/gigascience/giy062 (PMC6207142; doi:10.1093/gigascience/giy062)
Supplement: Reviewer_2_Report_(Original_Submission) -- Namshin Kim — 10/1/2017 Reviewed [file giy062_reviewer_2_report_(original_submission).pdf]

Reviewer Report

Title: Massive NGS Data Analysis Reveals Hundreds Of Potential Novel Gene Fusions in Human Cell Lines

Version: Original Submission **Date:** 10/1/2017

Reviewer name: Namshin Kim

Reviewer Comments to Author:

Authors presented manuscript titled "Massive NGS Data Analysis Reveals Hundreds Of Potential Novel Gene Fusions in Human Cell Lines", and they analyzed 935 RNA-Seq data from CCLE repository to find putative gene fusion events by three bionformatics software - FusionCatcher, EricScript, Tophat-Fusion.I have acessed the online database and have major question as below.First, one of software is giving *too many* fusion events compared with the other two. It means either one of software is giving incorrect results. Whatever the results are, if they are giving more than 10 times bigger reults than other software, it means it is not acceptable. There are a tons of software for this purpose - finding fusion genes. Authors need to be very careful when choosing some of them because detecting fusion events from RNA-Seq require very sophisticated optimization and filtering process as well as long calculation time. Authors need to do additional filtering steps for results from EricScript. Otherwise users will suspect something wrong with the final dataset.Second, p.4 line 18. Data Statistics and Validation section. Instead of overall statistics, 95% overlap with previously known cancer gene, please give how exactly it can detect experimentally validated fusion events from individual cell lines.Finally, web-server is just showing calculation results from three software. If one browses that database, he/she can only get information which software is giving this results. But no novel intuitions or dataming from each content.Thank you for our huge work, but readers need at least one scientific intuition or improvement from them. And, the database is very slow due to heavy use of javascript (I don't know exactly what WWW techniques are used). I think the database itself is not that big, and it could be improved.

Level of Interest

Please indicate how interesting you found the manuscript: An article of importance in its field

Quality of Written English

Please indicate the quality of language in the manuscript: Acceptable

Declaration of Competing Interests

Please complete a declaration of competing interests, considering the following questions:

- Have you in the past five years received reimbursements, fees, funding, or salary from an organisation that may in any way gain or lose financially from the publication of this manuscript, either now or in the future?
- Do you hold any stocks or shares in an organisation that may in any way gain or lose financially from the publication of this manuscript, either now or in the future?

- Do you hold or are you currently applying for any patents relating to the content of the manuscript?
- Have you received reimbursements, fees, funding, or salary from an organization that holds or has applied for patents relating to the content of the manuscript?
- Do you have any other financial competing interests?
- Do you have any non-financial competing interests in relation to this paper?

If you can answer no to all of the above, write 'I declare that I have no competing interests' below. If your reply is yes to any, please give details below.

I declare that I have no competing interests.

I agree to the open peer review policy of the journal. I understand that my name will be included on my report to the authors and, if the manuscript is accepted for publication, my named report including any attachments I upload will be posted on the website along with the authors' responses. I agree for my report to be made available under an Open Access Creative Commons CC-BY license (http://creativecommons.org/licenses/by/4.0/). I understand that any comments which I do not wish to be included in my named report can be included as confidential comments to the editors, which will not be published.

I agree to the open peer review policy of the journal

To further support our reviewers, we have joined with Publons, where you can gain additional credit to further highlight your hard work (see: https://publons.com/journal/530/gigascience). On publication of this paper, your review will be automatically added to Publons, you can then choose whether or not to claim your Publons credit. I understand this statement.

Yes
