# Supplementary material for: Massive NGS data analysis reveals hundreds of potential novel gene fusions in human cell lines
Source: Gigascience. 2018 Jun 1;7(10):giy062. doi: 10.1093/gigascience/giy062 (PMC6207142; doi:10.1093/gigascience/giy062)
Supplement: Reviewer_2_Report_(Revision_1) -- Namshin Kim — 1/16/2018 Reviewed [file giy062_reviewer_2_report_(revision_1).pdf]

**Reviewer Report**

**Title: Massive NGS Data Analysis Reveals Hundreds Of Potential Novel Gene Fusions in Human Cell Lines**

**Version: Revision 1 Date:** 1/16/2018

**Reviewer name: Namshin Kim**

**Reviewer Comments to Author:**

Authors presented revised version of manuscript "Massive NGS Data Analysis Reveals Hundreds Of Potential Novel Gene Fusions in Human Cell Lines" and their new website. It has been greatly improved over previous version.I tried to navigate using Chrome browser, but I still found some issues to be considered. First, in "Search by" tab, when I tried to input ALK gene in 3' partner, it is taking some time to load gene list and then select it. People usually get user's input and then search their database to return the results. Please consider what would be better to get user input.In Statistics page, please provide with color legends on colorful pie chart. (now it gives mouse-over legends)minor point: All the gene symbols are upper characters. for example, C9ORF66 gene, it is actually C9orf66 according to HGNC. I think authors did for their conveniences, but I think the gene symbols should follow HGNC nomenclature. Please compare and update your gene symbol according to latest version of HGNC and their aliases. It will be better to give official gene symbol and aliases. For example, official symbol of well-known HER2 gene is ERBB2. People tends to assume that all gene symbols are upper characters, but NOT.In each link, please give "EXACT" links rather than main page. for example, I noticed that a link for ALK gene is http://cancer.sanger.ac.uk/cosmic/census but it SHOULD be pointing to http://cancer.sanger.ac.uk/cosmic/gene/analysis?ln=ALK as well as genecards http://www.genecards.org/cgi-bin/carddisp.pl?gene=ALK&keywords=ALK Please find all links and give their EXACT url links, NOT top page.In fusion gene info, you used NPM1#ALK but in each info page, you used NPM1/ALK. Please use same separator.In each search page, can you delete mouse-over message "this fusion event is supported by...." I don't think this long message is giving valuable information. It is already under "FusionCatcher", "EricScript" and "Tophat-fusion". And I noticed that each icons are linked to same page, not even pubmed icon. I thought pubmed Icon can lead me to pubmed but NOT.Please give thorough examination on each page again, menu, and etc. I thought that you have answered all questions but I can still found many errors.

**Level of Interest**

Please indicate how interesting you found the manuscript: An article whose findings are important to those with closely related research interests

**Quality of Written English**

Please indicate the quality of language in the manuscript: Acceptable

**Declaration of Competing Interests**

Please complete a declaration of competing interests, considering the following questions:

- Have you in the past five years received reimbursements, fees, funding, or salary from an organisation that may in any way gain or lose financially from the publication of this manuscript, either now or in the future?
- Do you hold any stocks or shares in an organisation that may in any way gain or lose financially from the publication of this manuscript, either now or in the future?
- Do you hold or are you currently applying for any patents relating to the content of the manuscript?
- Have you received reimbursements, fees, funding, or salary from an organization that holds or has applied for patents relating to the content of the manuscript?
- Do you have any other financial competing interests?
- Do you have any non-financial competing interests in relation to this paper?

If you can answer no to all of the above, write 'I declare that I have no competing interests' below. If your reply is yes to any, please give details below.

I declare that I have no competing interests

I agree to the open peer review policy of the journal. I understand that my name will be included on my report to the authors and, if the manuscript is accepted for publication, my named report including any attachments I upload will be posted on the website along with the authors' responses. I agree for my report to be made available under an Open Access Creative Commons CC-BY license (http://creativecommons.org/licenses/by/4.0/). I understand that any comments which I do not wish to be included in my named report can be included as confidential comments to the editors, which will not be published.

I agree to the open peer review policy of the journal

To further support our reviewers, we have joined with Publons, where you can gain additional credit to further highlight your hard work (see: https://publons.com/journal/530/gigascience). On publication of this paper, your review will be automatically added to Publons, you can then choose whether or not to claim your Publons credit. I understand this statement.

Yes
