# Supplementary material for: Massive NGS data analysis reveals hundreds of potential novel gene fusions in human cell lines
Source: Gigascience. 2018 Jun 1;7(10):giy062. doi: 10.1093/gigascience/giy062 (PMC6207142; doi:10.1093/gigascience/giy062)
Supplement: Reviewer_3_Report_(Original_Submission) -- Daniel Nicorici — 10/3/2017 Reviewed [file giy062_reviewer_3_report_(original_submission).pdf]

Reviewer Report

Title: **Massive NGS Data Analysis Reveals Hundreds Of Potential Novel Gene Fusions in Human Cell Lines**

Version: **Original Submission** **Date:** 10/3/2017

Reviewer name: **Daniel Nicorici**

Reviewer Comments to Author:

The article presents that bioinformatics analysis and its results for calling candidate fusion genes in RNA-seq data from 935 cancer cell lines. The list of candidate fusion genes is available as a browseable database called LiGeA.On page 1, line 12, the "gene fusion events result from chromosomal rearrangements" should be changed to "oncogenic gene fusion events result from chromosomal rearrangements" because fusion genes occur also in healthy organisms. Fusion of genes is also one of the evolutionary mechanism for creating a new gene in a healthy organism. Not all fusion genes are oncogenic. For example, there are plenty of fusion genes known to exist in healthy people, like for example TTTY15-USP9Y, SLC45A3-ELK4, MSMB-NCOA4.On page 1, lines 37-40, the text "Moreover, each gene fusion predictions differes...chromosomal rearrangements (Mertens et al.; 2015)" should be removed from the article because it is not correct. This is not correct because some fusion gene finder can call very well a certain type of fusion genes whilst all the other fusion caller will miss the and therefore the consensus here is not the best. For example, FusionCatcher is the only fusion finder which is able to call IGH fusions (see: https://doi.org/10.1182/blood-2016-12-758979).When searching for a gene using the http://hpc-bioinformatics.cineca.it/fusion/search_for_gene is very slow. This should be fixed.Also it is very important to list and show the fusion genes which are supported only by one fusion finder. For example the fusion DUX4-IGH is known to exist in NALM6 cell line which is one of the 935 cell lines but when looking for it in LiGeA database, it does not show up because the TOPHAT-fusion and EricScript are not able to find fusions which involve DUX4 gene or IGH gene.

Level of Interest

Please indicate how interesting you found the manuscript: An article whose findings are important to those with closely related research interests

Quality of Written English

Please indicate the quality of language in the manuscript: Needs some language corrections before being published

Declaration of Competing Interests

Please complete a declaration of competing interests, considering the following questions:

- Have you in the past five years received reimbursements, fees, funding, or salary from an organisation that may in any way gain or lose financially from the publication of this manuscript, either now or in the future?

- Do you hold any stocks or shares in an organisation that may in any way gain or lose financially from the publication of this manuscript, either now or in the future?
- Do you hold or are you currently applying for any patents relating to the content of the manuscript?
- Have you received reimbursements, fees, funding, or salary from an organization that holds or has applied for patents relating to the content of the manuscript?
- Do you have any other financial competing interests?
- Do you have any non-financial competing interests in relation to this paper?

If you can answer no to all of the above, write 'I declare that I have no competing interests' below. If your reply is yes to any, please give details below.

No.

I agree to the open peer review policy of the journal. I understand that my name will be included on my report to the authors and, if the manuscript is accepted for publication, my named report including any attachments I upload will be posted on the website along with the authors' responses. I agree for my report to be made available under an Open Access Creative Commons CC-BY license (http://creativecommons.org/licenses/by/4.0/). I understand that any comments which I do not wish to be included in my named report can be included as confidential comments to the editors, which will not be published.

I agree to the open peer review policy of the journal

To further support our reviewers, we have joined with Publons, where you can gain additional credit to further highlight your hard work (see: https://publons.com/journal/530/gigascience). On publication of this paper, your review will be automatically added to Publons, you can then choose whether or not to claim your Publons credit. I understand this statement.

Yes
